# Triterpene-Based Carboxamides Act as Good Inhibitors of Butyrylcholinesterase

**DOI:** 10.3390/molecules24050948

**Published:** 2019-03-07

**Authors:** Anne Loesche, Michael Kahnt, Immo Serbian, Wolfgang Brandt, René Csuk

**Affiliations:** 1 Martin-Luther-University Halle-Wittenberg, Organic Chemistry, Kurt-Mothes-Str. 2, D-06120 Halle (Saale), Germany; anne.loesche@chemie.uni-halle.de (A.L.); michael.kahnt@chemie.uni-halle.de (M.K.); immo.serbian@chemie.uni-halle.de (I.S.); 2 Leibniz Institute of Plant Biochemistry, Bioorganic Chemistry, Weinberg 3, D-06120 Halle (Saale), Germany; wbrandt@ipb-halle.de

**Keywords:** acetylcholinesterase, butyrylcholinesterase, triterpenoids

## Abstract

A set of overall 40 carboxamides was prepared from five different natural occurring triterpenoids including oleanolic, ursolic, maslinic, betulinic, and platanic acid. All of which were derived from ethylene diamine holding an additional substituent connected to the ethylene diamine group. These derivatives were evaluated regarding their inhibitory activity of the enzymes acetylcholinesterase (AChE) and butyrylcholinesterase (BChE) employing Ellman’s assay. We further determined the type of inhibition and inhibition constants. Carboxamides derived from platanic acid have been shown to be potent and selective BChE inhibitors. Especially the mixed-type inhibitor (3β)-*N*-(2-pyrrolidin-1-ylethyl)-3-acetyloxy-20-oxo-30-norlupan-28-amide (**35**) showed a remarkably low K_i_ of 0.07 ± 0.01 µM (K_i_′ = 2.38 ± 0.48 µM) for the inhibition of BChE.

## 1. Introduction

It is now more than a century since Alois Alzheimer, a German physician, described a new disease of the brain [1], being today one of the most threatening plagues for the elderly and one of the greatest challenges for chemists and biologists. The eponymous disease is today one of the greatest scourges of humanity. Alzheimer’s disease (AD), the most common cause of dementia, is a neurodegenerative disorder, causing the progressive loss of cognitive functions and memory. As a result of demographic changes, the number of AD patients is steadily rising. This disease is characterized by an increasing decline in acetylcholine (ACh, neurotransmitter) levels in the cholinergic system [2]. A common strategy for the management of AD is to develop inhibitors that suppress the degradation of ACh caused by hydrolases acetylcholinesterase (AChE, EC 3.1.1.7) and butyrylcholinesterase (BChE, EC 3.1.1.8).

**Figure 1:**
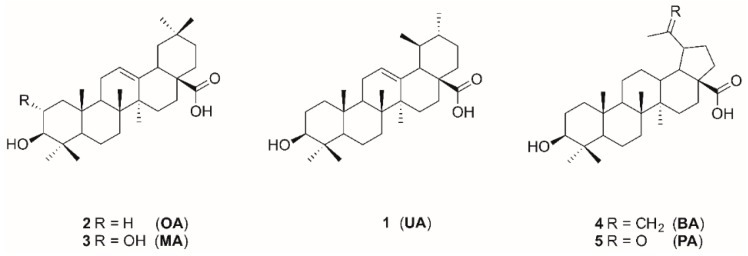
Structures of triterpenoic acids **1**–**5**.

Ursolic acid (**UA**), oleanolic acid (**OA**), maslinic acid (**MA**), betulinic acid (**BA**), and platanic acid (**PA**) are naturally occurring pentacyclic triterpenoids (Figure 1), which are widely distributed in various plants. Triterpenes and their derivatives represent a group of pharmacologically interesting substances showing a variety of biological activities such as antitumor [3], antiviral, and anti-HIV [4,5], antibacterial [6,7], and antifungal [8] properties. Pentacyclic triterpenoic acids have already been tested for cholinergic activities with moderate anti-cholinesterase activity [9,10]. Cyclic terpene derivatives, which act as inhibitors of cholinesterases, have been our research focus for a long time. Results from our group have demonstrated that structural modifications of the terpenoid backbone have a high impact onto the inhibitory potential for cholinesterases (ChEs) [11,12,13]. Exceptionally good ChE inhibition has been found for some amino derivatives of platanic acid [12], as well as for amides of dehydroabietylamine [14] and 12-hydroxydehydroabietylamine [15].

In patients, BChE activity increases with progression of AD while the level of AChE remains constant [16]. Therefore, selective BChE inhibitors represent legitimate therapeutic options to improve the deficit in the neurotransmitter ACh. Deduced from the results of our previous studies, **UA**, **OA**, **MA**, **BA** and **PA** carboxamides holding a terminal amino moiety were synthesized from the parent pentacyclic triterpenoic acids. Altogether, 40 different compounds were synthesized and screened for their ChE inhibitory activity using Ellman’s assay.

## 2. Results and Discussion

### 2.1. Chemistry

**Scheme 1:**
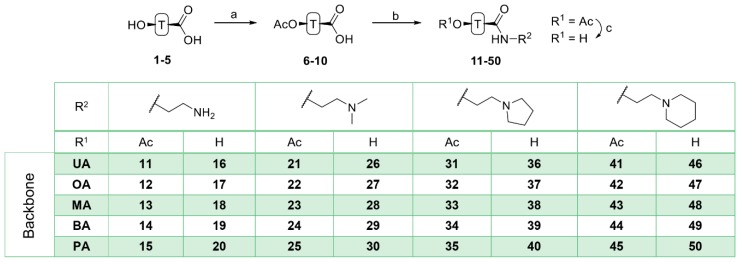
Generalized representation of **1**–**5** and preparation of triterpenoic carboxamides **11**–**50**: (a) Ac_2_O, DCM, TEA, 25 °C, 2 days, 90–96%; (b) oxalyl chloride, DCM, DMF, 0–25 °C, 1 h, then amine, 25 °C, 2 h; (**c**) MeOH/KOH, 25 °C, 2–3 days.

Acetylation of triterpenoic acids (Figure 1, **1**–**5**) furnished acetates **6**–**10** (Scheme 1). Reaction of **6**–**10** with oxalyl chloride and subsequent treatment with various amines derived from ethylene diamine gave carboxamides **11**–**15**, **21**–**25**, **31**–**35** and **41**–**45**. Their deacetylation with KOH/MeOH gave compounds **16**–**20**, **26**–**30**, **36**–**40** and **46**–**50**, respectively.

### 2.2. Biology

**Figure 2:**
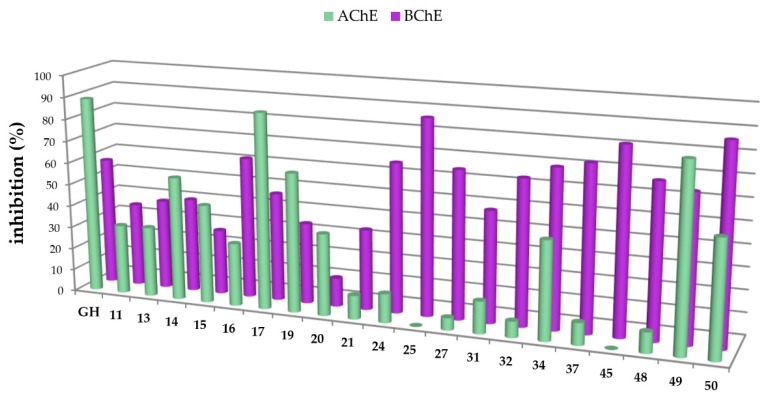
Percentage of inhibition of selected carboxamides and galantamine hydrobromide (**GH** as standard), final concentration of the inhibitor 10 µM, determined by Ellman’s assay using acetylcholinesterase (green in the front) and butyrylcholinesterase (purple in the back).

**Figure 3:**
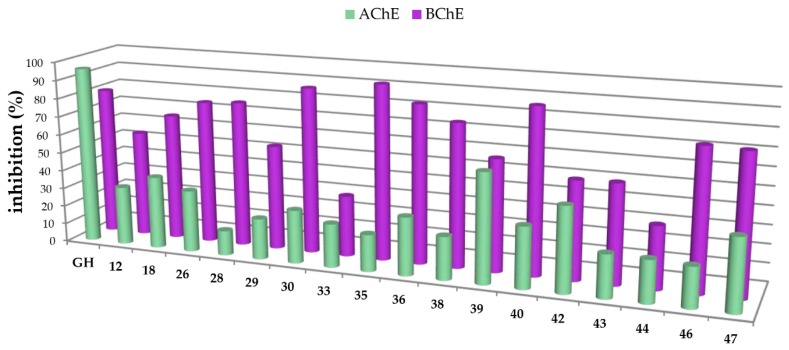
Percentage of inhibition of selected carboxamides and galantamine hydrobromide (**GH** as standard), final concentration of the inhibitor 30 µM, determined by Ellman’s assay using acetylcholinesterase (green in the front) and butyrylcholinesterase (purple in the back).

In this study, carboxamides with different triterpenoic backbones (ursolic, oleanolic, maslinic, betulinic, and platanic acids), and various amine residues (Scheme 1), were investigated. All compounds **11**–**50** (except those being not soluble under the conditions of the assay) were subjected to Ellman’s assays to determine their inhibition rates and constants (K_i_ for competitive inhibition and K_i_′ for uncompetitive inhibition) for the cholinesterases AChE and BChE. Galantamine hydrobromide (**GH**), one of the gold standard drugs for treating AD symptoms, was used for comparison. In general, pre-screening the 37 triterpenoic acid amides using AChE (electric eel) and BChE (equine serum) identified seventeen compounds as potential inhibitors with inhibition rates equal or even better than **GH**. The results are summarized in Figure 2 and Figure 3 (details are found in the Supplementary Part, Table S1).

The carboxamides showed a considerably higher inhibition for BChE than for AChE. With the exception of **17** (88.61 ± 0.22%) and **49** (82.72 ± 0.09%), none of the tested compounds showed any notable activity for AChE. Further kinetic studies (see Supplementary Part, Table S2) showed (3β)-*N*-(2-piperidin-1-ylethyl)-3-hydroxy-lup-20(29)-en-28-amide (**49**) as a good mixed-type AChE inhibitor with inhibition constants: K_i_ = 1.00 ± 0.09 µM and K_i_′ = 1.42 ± 0.08 μM. The results of the screening experiments suggest that many of the synthesized carboxylic acid amides inhibit BChE activity to a high degree. Fifteen derivatives showed promising inhibitory rates, five of which seemed to inhibit BChE almost completely (88.37–94.60% inhibition rate). The evaluation of the Dixon [17], Cornish-Bowden [18], and Lineweaver-Burk [19] plots showed that all active compounds were mixed-type inhibitors with a dominating competitive part. The results from these measurements are compiled in Table 1 and Table 2.

The first set of compounds holding an aminoethyl residue, **11**–**14** and **16**–**19**, were moderate inhibitors of BChE, with oleanolic acid derivative **17** as the only noteworthy compound of this series (Ki = 1.93 ± 0.13 µM and K_i_′ = 7.68 ± 0.61 μM). The next set consisting of triterpenoic amides with a dimethylaminoethyl substituent (**21**–**24** and **26**–**29**) showed good inhibition with K_i_ values between 1.1 and 6.5 µM, respectively. For 2-piperidin-1-ylethyl substituted derivatives (**41**–**44** and **46**–**49**) inhibition constants in the same range were obtained. The group of 2-pyrrolidin-1-ylethyl substituted compounds (**31**–**34** and **36**–**39**) delivered the best results: derivatives **34** (from betulinic acid), **36** (ursolic acid backbone) and **37** (from oleanolic acid), held excellent K_i_ values of 0.39 ± 0.04 µM, 0.47 ± 0.08 µM, and 0.55 ± 0.02 µM, respectively. Compounds showing the highest selectivity (F, expressed by percent inhibition for AChE divided by percent inhibition for BChE) towards BChE were compounds **27** (F = 0.08) and **32** (F = 0.11). 

Because of the known inhibitory effect [12], a set of eight platanic acid derivatives was investigated (Table 2). Compounds with an aminoethyl residue (**15** and **20**) were the weakest BChE inhibitors in our test. Compared to the standard **GH** (K_i_ = 2.30 ± 0.17 µM), 2-(dimethylamino)ethyl substituted derivatives showed good inhibition constants (K_i_ = 1.60 ± 0.13 µM for **25** and K_i_ = 1.12 ± 0.01 µM for **30**).

Compounds, carrying a piperidinyl group, showed inhibition even in nanomolar concentrations. For compound **45** (K_i_ = 0.71 ± 0.04 µM) and **50** (K_i_ = 0.97 ± 0.04 µM) excellent values were observed. As already described above, introducing a pyrrolidinyl moiety resulted in compounds with excellent K_i_ values. A very low inhibition constant (K_i_ = 0.45 ± 0.01 µM) was measured for compound **40**. The most active compound was (3β)-*N*-(2-pyrrolidin-1-ylethyl)-3-acetyloxy-20-oxo-30-norlupan-28-amide (**35**) showing inhibition constants of K_i_ = 0.07 ± 0.01 µM and K_i_′ = 2.38 ± 0.48 µM for BChE. This compound proved to be a selective (F ≈ 0.21) inhibitor for BChE. Except for **15** and **20**, all **PA**-derived compounds showed reasonably high BChE selectivity. 

To explain the results from the biological testing, some molecular modeling studies were performed. Table 3 summarizes the results for the most favored docking position of each ligand. From these results, nice correlations between the gold fitness values (the higher the value the better the predicted affinity between ligand enzyme), as well as the interaction energies, with the experimental K_i_ values were established.

**Figure 4:**
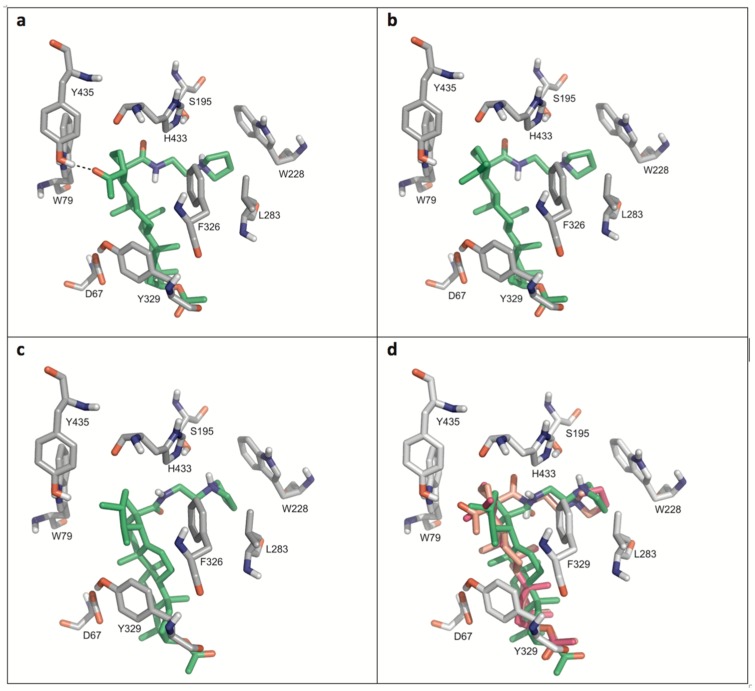
Details of the most favored docking arrangements of **35** (**a**), **34** (**b**) and **31** (**c**). (**d**) An overlay of all three ligands (orange colored: carbon atoms of **35**; magenta colored: carbon atoms of **34**; green colored: carbon atoms of **31**). Hydrophobic interactions with the side chains of W79, W228, L283, Y329, and F326 preferentially stabilize the docking poses. As for **35** (**a**) a hydrogen bond with Y435 is formed which might explain the higher affinity compared to the other compounds.

**Figure 5:**
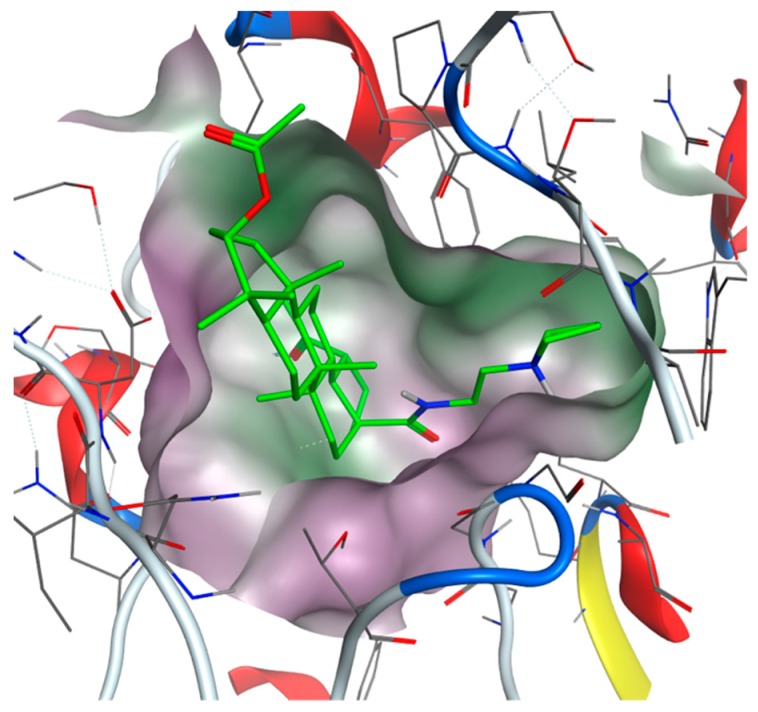
View from the top to the binding site of BChE with bound compound **35** (green colored: carbon atoms) with displayed lipophilic potential (green surface). The more hydrophilic site is colored magenta.

Figure 4 displays details of the docking arrangements of ligands with BChE. All ligands fit into the binding pocket—preferentially based on hydrophobic interactions (Figure 5) with the side chains of residues W79, W228, L283, Y329, and F326. The highest affinity was observed for **35** (Figure 4a). Thus a hydrogen bond of the acetyl group of **35** with the phenolic hydroxyl group of Y79 stabilizes the interaction with BChE. This hydrogen bond, however, is not present in compounds **31** and **34**. The modifications in ring E of **31** (six-membered ring) compared to compounds **34** and **35** (five-membered rings) slightly changed the docking arrangement (cf. Figure 4d) and caused reduced hydrophobic interactions in particular with W79.

## 3. Materials and Methods

### 3.1. General

All chemicals, reagents and technical equipment were purchased in Germany unless otherwise stated. NMR spectra were recorded using the Varian spectrometers Gemini 2000 or Unity 500 (δ given in ppm, *J* in Hz; typical experiments: H-H-COSY, HMBC, HSQC, NOESY), MS spectra were taken on a Finnigan MAT LCQ 7000 (electrospray, voltage 4.1 kV, sheath gas nitrogen) instrument. The optical rotations were measured on a Perkin-Elmer polarimeter at 20 °C; TLC was performed on silica gel (Merck 5554, detection with cerium molybdate reagent) melting points were uncorrected (*Leica* hot stage microscope or BÜCHI Melting Point M-565) and elemental analyses were performed on a Foss-Heraeus Vario EL (CHNS) unit. IR spectra were recorded on a Perkin Elmer FT-IR spectrometer Spectrum 1000 or on a Perkin-Elmer Spectrum Two (UATR Two Unit). The solvents were dried according to usual procedures. The purity of the compounds was determined by HPLC and found to be >96%. Ursolic (**1**), betulinic (**4**), and platanic acid (**5**) were obtained from Betulinines (Stříbrná Skalice, Czech Republic), oleanolic acid (**2**) was purchased from Carbone Scientific (London, UK) and maslinic acid (**3**) was synthesized as previously described [20,21].

### 3.2. Biology

A TECAN SpectraFluorPlus working in the kinetic mode and measuring the absorbance at λ = 415 nm was used for the enzymatic studies. Acetylcholinesterase (from *Electrophorus electricus*), 5,5′-dithiobis-(2-nitrobenzoic acid) (DTNB) and acetylthiocholine iodide were purchased from Sigma. Butyrylcholinesterase (from equine serum) was purchased from Fluka. Preparation of the solutions, assay procedure, as well as molecular modeling conditions can be found in the Supplementary Materials.

## 4. Experimental

### 4.1. General

The preparation of the platanic carboxamides **15**, **20**, **25**, **30**, **35**, and **40** was performed as previously described [12,22]. Betulinic and ursolic carboxamides **11**, **14**, **16**, **19**, **21**, **24**, **26**, **29**, **31**, **34**, **36**, **39**, **41**, **44**, **46**, and **49** were prepared as previously reported in the literature [23]. Experimental procedures and full analytical data of these compounds can be found in the Supplementary Materials.

### 4.2. General Procedure A for the Acetylation of Triterpenoic Acids (***6***–***10***)

To a solution of triterpenoic acid **1**–**5** (11 mmol) in dry DCM (150 mL) was added triethylamine (4.6 mL, 33 mmol), acetic anhydride (3.1 mL, 33 mmol) and DMAP (cat.). After stirring for two days at 25 °C, a saturated solution of NH_3_ in MeOH was added (3 mL) and the mixture was stirred for another 30 min. Dilution with DCM and subsequent aqueous work-up provided crude acetates. Recrystallization from EtOH yielded acetates **6**–**10** (90–96%) as colorless solids, whose spectroscopic data were in full agreement with data from the literature.

### 4.3. General Procedure B for the Synthesis of Triterpenoic Amides (***11***–***15***, ***21***–***25***, ***31***–***35***, and ***41***–***45***)

Triterpenoic acetates **6**–**10** (0.8 mmol) were each dissolved in dry DCM (15 mL), cooled to 0 °C, then oxalyl chloride (3.2 mmol) and dry DMF (3 drops) were added. After warming to 25 °C, the mixture was stirred for 1 h. The solvent was removed under reduced pressure, re-evaporated with dry THF (4 × 15 mL), and the residue was immediately resolved in dry DCM (10 mL). This solution was then added dropwise to a solution of the amino compound (2.4 mmol) in dry DCM (5 mL) and stirred at 25 °C for 2 h. After usual aqueous work-up, the solvent was removed under reduced pressure and the crude products were subjected to column chromatography (silica gel, chloroform/methanol mixtures). Compounds **11**–**15**, **21**–**25**, **31**–**35**, and **41**–**45** were each obtained as colorless solids. 

### 4.4. General Procedure C for the Deacetylation of Triterpenoic Amides (***16***–***20***, ***26***–***30***, ***36***–***40***, and ***46***–***50***)

To a solution of the acetylated amide (0.33 mmol) in methanol (10 mL) was added a solution of potassium hydroxide (1.65 mmol) in methanol (2 mL). The mixture was stirred at 25 °C for 2 or 3 days. After completion of the reaction (as indicated by TLC), aq. HCl was added until pH = 7. After usual work-up, the solvent was removed under reduced pressure and the residue was subjected to column chromatography (silica gel, chloroform/methanol mixtures) yielding compounds **16**–**20**, **26**–**30**, **36**–**40**, and **46**–**50** each as colorless solids.

*(3*β*)-3-Acetyloxy-urs-12-en-28-oic acid* (**6**), Compound **6** was prepared according to general procedure A from ursolic acid (**1**). Yield: 96%; m.p. 287–290 °C (lit.: 289–290 °C [24]).

*(3*β*)-3-Acetyloxy-olean-12-en-28-oic acid* (**7**), Compound **7** was prepared according to general procedure A from oleanolic acid (**2**). Yield: 90%; m.p. 259–261 °C (lit.: 255–257 °C [25]).

*(2*α*,3*β*)-2,3-Diacetyloxy-olean-12-en-28-oic acid* (**8**), Compound **8** was prepared according to general procedure A from maslinic acid (**3**). Yield: 91%; m.p. 172–175 °C (l.: 170–173 °C [26]).

*(3*β*)-3-Acetyloxy-lup-20(29)en-28-oic acid* (**9**), Compound **9** was prepared according to general procedure A from betulinic acid (**4**). Yield: 93%; m.p. 281–284 °C (lit.: 280–282 °C [27]).

*(3*β*)-3-Acetyloxy-20-oxo-30-norlupan-28-oic acid* (**10**), Compound **10** was prepared according to general procedure A from platanic acid (**5**). Yield: 94%; m.p. 256–259 °C (lit.: 252–255 °C [28]).

*(3*β*)-N-(2-Aminoethyl)-3-acetyloxy-olean-12-en-28-amide* (**12**), Compound **12** was prepared from **7** according to general procedure B using ethylenediamine as amino compound. Column chromatography (SiO_2_, CHCl_3_/MeOH 9:1) gave **12** (yield: 75%); m.p. 212–215 °C (decomp.); [α]_D_ = +37.8° (c 0.350, CHCl_3_); R_f_ = 0.67 (CHCl_3_/MeOH/NH_4_OH 90:10:1); IR (ATR): ν = 2944 m, 1732 m, 1628 m, 1523 m, 1364 s, 1244 s, 1027 m, 985 m, 824 m, 752 m cm^−1^; ^1^H-NMR (400 MHz, CDCl_3_): δ = 7.04 (t, J = 5.5 Hz, 1H, NH), 5.40 (t, J = 3.4 Hz, 1H, 12-H), 4.53–4.45 (m, 1H, 3-H), 3.68–3.56 (m, 1H, 31-H_a_), 3.40–3.29 (m, 1H, 31-H_b_), 3.24–3.11 (m, 2H, 32-H), 2.65 (dd, J = 12.7, 4.6 Hz, 1H, 18-H), 2.04 (s, 3H, Ac), 2.01–1.82 (m, 3H, 16-H_a_, 11-H_a_, 11-H_b_), 1.79–1.22 (m, 14H, 19-H_a_, 1-H_a_, 2-H_a_, 2-H_b_, 7-H_a_, 7-H_b_, 9-H, 16-H_b_, 6-H_a_, 15-H_a_, 22-H_a_, 6-H_b_, 21-H_a_, 22-H_b_), 1.22–1.11 (m, 2H, 19-H_b_, 21-H_b_), 1.14 (s, 3H, 27-H), 1.10–0.95 (m, 2H, 1-H_b_, 15-H_b_), 0.93 (s, 3H, 25-H), 0.90 (s, 3H, 30-H), 0.89 (s, 3H, 29-H), 0.86 (s, 3H, 23-H), 0.85 (s, 3H, 24-H), 0.84–0.79 (m, 1H, 5-H), 0.73 (s, 3H, 26-H) ppm; ^13^C-NMR (101 MHz, CDCl_3_): δ = 180.8 (C-28), 171.0 (Ac), 144.0 (C-13), 123.1 (C-12), 80.7 (C-3), 55.2 (C-5), 47.5 (C-9), 46.6 (C-19), 46.3 (C-17), 41.8 (C-14), 41.4 (C-18), 40.4 (C-32), 39.4 (C-8), 38.2 (C-1), 38.1 (C-31), 37.7 (C-4), 36.9 (C-10), 34.2 (C-21), 33.1 (C-30), 32.3 (C-22), 32.2 (C-7), 30.6 (C-20), 28.0 (C-23), 27.2 (C-15), 25.8 (C-27), 23.6 (C-2), 23.5 (C-16), 23.5 (C-11), 23.5 (C-29), 21.3 (Ac), 18.2 (C-6), 16.9 (C-26), 16.7 (C-24), 15.5 (C-25) ppm; MS (ESI, MeOH): m/z = 541.3 (100%, [M + H]^+^); analysis calcd. for C_34_H_56_N_2_O_3_ (540.83): C 75.51, H 10.44, N 5.18; found: C 75.42, H 10.57, N 5.07.

*(2*α*,3*β*)-N-(2-Aminoethyl)-2,3-diacetyloxy-olean-12-en-28-amide* (**13**), Compound **13** was prepared from **8** according to general procedure B using ethylenediamine as amino compound. Column chromatography (SiO_2_, CHCl_3_/MeOH 9:1) gave **13** (yield: 74%); m.p. 151–154 °C; [α]_D_ = +18.7° (c 0.330, CHCl_3_); R_f_ = 0.63 (CHCl_3_/MeOH/NH_4_OH 90:10:1); IR (KBr): ν = 3426 br s, 2946 s, 1742 s, 1636m, 1522m, 1458m, 1436w, 1368m, 1254s, 1044m cm^−1^; ^1^H-NMR (400 MHz, CDCl_3_): δ = 6.36 (t, J = 5.5 Hz, 1H, NH), 5.37 (t, J = 3.6 Hz, 1H, 12-H), 5.08 (ddd, J = 11.1, 10.9, 4.6 Hz, 1H, 2-H), 4.73 (d, J = 10.3 Hz, 1H, 3-H), 3.48–3.39 (m, 1H, 31-H_a_), 3.12–3.02 (m, 1H, 31-H_b_), 2.87–2.76 (m, 2H, 32-H), 2.56 (dd, J = 13.1, 4.3 Hz, 1H, 18-H), 2.04 (s, 3H, Ac), 2.08–1.83 (m, 4H, 1-H_a_, 16-H_a_, 11-H_a_, 11-H_b_), 1.97 (s, 3H, Ac), 1.80–1.24 (m, 11H, 19-H_a,_ 22-H_a_, 16-H_b_, 9-H, 22-H_b_, 6-H_a_, 15-H_a_, 7-H_a_, 6-H_b_, 21-H_a_, 7-H_b_), 1.23–1.09 (m, 2H, 21-H_b_, 19-H_b_), 1.14 (s, 3H, 27-H), 1.11–0.99 (m, 2H, 1-H_b_, 15-H_b_), 1.04 (s, 3H, 25-H), 0.99–0.92 (m, 1H, 5-H), 0.90 (s, 9H, 24-H, 29-H, 30-H), 0.89 (s, 3H, 23-H), 0.76 (s, 3H, 26-H) ppm; ^13^C-NMR (101 MHz, CDCl_3_): δ = 178.8 (C-28), 170.9 (Ac), 170.7 (Ac), 144.9 (C-13), 122.5 (C-12), 80.7 (C-3), 70.1 (C-2), 54.9 (C-5), 47.6 (C-9), 46.8 (C-19), 46.5 (C-17), 44.0 (C-1), 42.3 (C-18), 42.2 (C-14), 41.8 (C-31), 41.4 (C-32), 39.6 (C-8), 39.5 (C-4), 38.2 (C-10), 34.3 (C-21), 33.1 (C-30), 32.9 (C-22), 32.3 (C-7), 30.9 (C-20), 28.5 (C-23), 27.4 (C-15), 25.9 (C-27), 23.8 (C-16), 23.8 (C-29), 23.7 (C-11), 21.3 (Ac), 21.0 (Ac), 18.3 (C-6), 17.8 (C-24), 17.1 (C-26), 16.6 (C-25) ppm; MS (ESI, MeOH): m/z = 599 (100%, [M + H]^+^); analysis calcd. for C_36_H_58_N_2_O_5_ (598.87): C 72.20, H 9.76, N 4.68; found: C 72.01, H 9.92, N 4.44.

*(3*β*)-N-(2-Aminoethyl)-3-hydroxy-olean-12-en-28-amide* (**17**), Compound **17** was prepared from **12** according to general procedure C. Column chromatography (SiO_2_, CHCl_3_/MeOH/NH_4_OH 90:10:0.1) gave **17** (yield: 80%); m.p. 221–224 °C (decomp.); [α]_D_ = +55.3° (c 0.315, CHCl_3_); R_f_ = 0.61 (CHCl_3_/MeOH/NH_4_OH 90:10:1); IR (ATR): ν = 3374w, 2941m, 1622m, 1530m, 1456m, 1387s, 1361s, 1344s, 1322s, 1187m, 1137m, 1097m, 1023m, 996m, 825m cm^−1^; ^1^H-NMR (500 MHz, CD_3_OD): δ = 5.36 (t, J = 3.7 Hz, 1H, 12-H), 3.50–3.43 (m, 1H, 31-H_a_), 3.37–3.32 (m, 1H, 31-H_b_), 3.15 (dd, J = 11.3, 4.8 Hz, 1H, 3-H), 3.07–2.95 (m, 2H, 32-H), 2.79 (dd, J = 13.2, 4.4 Hz, 1H, 18-H), 2.10 (ddd, J = 13.8, 13.0, 4.0 Hz, 1H, 16-H_a_), 1.99–1.86 (m, 2H, 11-H_a_, 11-H_b_), 1.83–1.74 (m, 1H, 19-H_a_), 1.70–1.35 (m, 12H, 22-H_a_, 1-H_a_, 9-H, 15-H_a_, 2-H_a_, 2-H_b_, 16-H_b_, 6-H_a_, 22-H_b_, 7-H_a_, 6-H_b_, 21-H_a_), 1.33–1.27 (m, 1H, 7-H_b_), 1.25–1.14 (m, 2H, 21-H_b_, 19-H_b_), 1.18 (s, 3H, 27-H), 1.11–0.99 (m, 2H, 15-H_b_, 1-H_b_), 0.97 (s, 3H, 23-H), 0.96 (s, 3H, 29-H), 0.94 (s, 3H, 25-H), 0.92 (s, 3H, 30-H), 0.79 (s, 3H, 26-H), 0.78 (s, 3H, 24-H), 0.77–0.74 (m, 1H, 5-H) ppm; ^13^C-NMR (126 MHz, CD_3_OD): δ = 182.0 (C-28), 145.0 (C-13), 124.2 (C-12), 79.6 (C-3), 56.7 (C-5), 49.0 (C-9), 47.6 (C-17), 47.6 (C-19), 42.9 (C-14), 42.4 (C-18), 40.8 (C-32), 40.7 (C-8), 39.8 (C-4), 39.8 (C-1), 38.8 (C-31), 38.1 (C-10), 35.0 (C-21), 34.2 (C-22), 33.8 (C-7), 33.5 (C-30), 31.6 (C-20), 28.7 (C-23), 28.5 (C-15), 27.8 (C-2), 26.5 (C-27), 24.5 (C-11), 24.0 (C-29), 24.0 (C-16), 19.5 (C-6), 17.9 (C-26), 16.3 (C-24), 15.9 (C-25) ppm; MS (ESI, MeOH): m/z = 499.3 (100%, [M + H]^+^); analysis calcd. for C_32_H_54_N_2_O_2_ (498.80): C 77.06, H 10.91, N 5.62; found: C 76.90, H 11.05, N 5.41.

*(2*α*,3*β*)-N-(2-Aminoethyl)-2,3-dihydroxy-olean-12-en-28-amide* (**18**), Compound **18** was prepared from **13** according to general procedure C. Column chromatography (SiO_2_, CHCl_3_/MeOH 9:1) gave **18** (yield: 60%); m.p. 260–266 °C (decompn.); [α]_D_ = +47.6° (c 0.335, CHCl_3_); R_f_ = 0.36 (CHCl_3_/MeOH/NH_4_OH 90:10:1); IR (KBr): ν = 3424 br s, 944m, 1636m, 1528m, 1460w, 1384w, 1166w, 1050m cm^−1^; ^1^H-NMR (400 MHz, CD_3_OD): δ = 5.36 (t, J = 3.7 Hz, 1H, 12-H), 3.61 (ddd, J = 11.3, 9.5, 4.5 Hz, 1H, 2-H), 3.43–3.35 (m, 1H, 31-H_a_), 3.29–3.22 (m, 1H, 31-H_b_), 2.90 (d, J = 9.6 Hz, 1H, 3-H), 2.89 (t, J = 6.5 Hz, 2H, 32-H), 2.81 (dd, J = 13.5, 4.5 Hz, 1H, 18-H), 2.14–2.03 (m, 1H, 16-H_a_), 2.03–1.88 (m, 3H, 11-H_a_, 11-H_b_, 1-H_a_), 1.78 (t, J = 13.5 Hz, 1H, 19-H_a_), 1.70–1.26 (m, 10H, 9-H, 22-H_a_, 15-H_a_, 16-H_b_, 6-H_a_, 22-H_b_, 7-H_a_, 6-H_b_, 21-H_a_, 7-H_b_), 1.23–1.13 (m, 2H, 19-H_b_, 21-H_b_), 1.18 (s, 3H, 27-H), 1.10–1.02 (m, 1H, 15-H_b_), 1.01 (s, 3H, 23-H), 1.00 (s, 3H, 25-H), 0.95 (s, 3H, 30-H), 0.91 (s, 3H, 29-H), 0.93–0.89 (m, 1H, 1-H_b_), 0.88–0.82 (m, 1H, 5-H), 0.80 (s, 3H, 24-H), 0.78 (s, 3H, 26-H) ppm; ^13^C-NMR (101 MHz, CD_3_OD): δ = 181.5 (C-28), 145.2 (C-13), 123.9 (C-12), 84.4 (C-3), 69.4 (C-2), 56.6 (C-5), 48.9 (C-9), 48.1 (C-1), 47.6 (C-17), 47.5 (C-19), 42.9 (C-14), 42.5 (C-18), 41.2 (C-32), 40.7 (C-8), 40.5 (C-4), 40.4 (C-31), 39.2 (C-10), 35.1 (C-21), 34.3 (C-22), 33.7 (C-7), 33.5 (C-30), 31.6 (C-20), 29.3 (C-23), 28.5 (C-15), 26.5 (C-27), 24.6 (C-11), 24.0 (C-29), 23.9 (C-16), 19.5 (C-6), 17.9 (C-26), 17.4 (C-24), 17.1 (C-25) ppm; MS (ESI, MeOH): m/z = 515 (100%, [M + H]^+^); analysis calcd. for C_32_H_54_N_2_O_3_ (514.80): C 74.66, H 10.57, N 5.44; found: C 74.49, H 10.74, N 5.28.

*(3*β*)-N-[2-(Dimethylamino)-ethyl]-3-acetyloxy-olean-12-en-28-amide* (**22**), Compound **22** was prepared from **7** according to general procedure B using N,N-dimethylethylene diamine as amino compound. Column chromatography (SiO_2_, CHCl_3_/MeOH 95:5) gave **22** (yield: 95%); m.p. 227–230 °C (decomp.), Lit.: 255 °C (decomp.) [29]; [α]_D_ = +53.0° (c 0.320, CHCl_3_), Lit.: +51.8° (c 0.34, CHCl_3_)[29]; R_f_ = 0.38 (CHCl_3_/MeOH 9:1); IR (ATR): ν = 2944m, 1732m, 1640w, 1464m, 1365m, 1244s, 1027m, 986m, 753m cm^−1^; ^1^H-NMR (400 MHz, CDCl_3_): δ = 6.77 (t, J = 5.3 Hz, 1H, NH), 5.38 (t, J = 3.6 Hz, 1H, 12-H), 4.51–4.45 (m, 1H, 3-H), 3.61–3.51 (m, 1H, 31-H_a_), 3.37–3.27 (m, 1H, 31-H_b_), 2.89–2.83 (m, 2H, 32-H), 2.65–2.56 (m, 1H, 18-H), 2.61 (s, 6H, 33-H, 33′-H), 2.03 (s, 3H, Ac), 2.02–1.93 (m, 1H, 16-H_a_), 1.93–1.85 (m, 2H, 11-H_a_, 11-H_b_), 1.72 (t, J = 13.4 Hz, 1H, 19-H_a_), 1.66–1.22 (m, 13H, 22-H_a_, 1-H_a_, 2-H_a_, 2-H_b_, 16-H_b_, 9-H, 22-H_b_, 6-H_a_, 15-H_a_, 7-H_a_, 6-H_b_, 21-H_a_, 7-H_b_), 1.21–1.14 (m, 2H, 19-H_b_, 21-H_b_), 1.14 (s, 3H, 27-H), 1.10–0.97 (m, 2H, 1-H_b_, 15-H_b_), 0.92 (s, 3H, 25-H), 0.90 (s, 3H, 29-H), 0.89 (s, 3H, 30-H), 0.85 (s, 3H, 23-H), 0.84 (s, 3H, 24-H), 0.83–0.79 (m, 1H, 5-H), 0.74 (s, 3H, 26-H) ppm; ^13^C-NMR (101 MHz, CDCl_3_): δ = 179.3 (C-28), 171.1 (Ac), 144.2 (C-13), 123.1 (C-12), 81.0 (C-3), 57.8 (C-32), 55.4 (C-5), 47.6 (C-9), 46.6 (C-19), 46.5 (C-17), 44.7 (C-33, C-33′), 42.0 (C-14), 41.9 (C-18), 39.6 (C-8), 38.3 (C-1), 37.8 (C-4), 37.0 (C-10), 36.1 (C-31), 34.3 (C-21), 33.2 (C-30), 32.9 (C-22), 32.5 (C-7), 30.8 (C-20), 28.2 (C-23), 27.5 (C-15), 25.9 (C-27), 23.7 (C-29), 23.6 (C-16, C-2, C-11), 21.4 (Ac), 18.3 (C-6), 17.1 (C-24), 16.8 (C-26), 15.6 (C-25) ppm; MS (ESI, MeOH): m/z = 569.5 (100%, [M + H]^+^); analysis calcd. for C_36_H_60_N_2_O_3_ (568.89): C 76.01, H 10.63, N 4.92; found: C 75.86, H 10.83, N 4.77.

*(2*α*,3*β*)-N-[2-(Dimethylamino)ethyl]-2,3-diacetyloxy-olean-12-en-28-amide* (**23**), Compound **23** was prepared from **8** according to general procedure B using N,N-dimethylethylene diamine as amino compound. Column chromatography (SiO_2_, CHCl_3_/MeOH 9:1) gave **23** (yield: 94%); m.p. 131–136 °C; [α]_D_ = +17.2° (c 0.340, CHCl_3_); R_f_ = 0.31 (CHCl_3_/MeOH 95:5); IR (KBr): ν = 3426br s, 2946s, 2772w, 1744s, 1654m, 1512m, 1462m, 1368m, 1252s, 1156w, 1044m cm^−1^; ^1^H-NMR (400 MHz, CDCl_3_): δ = 6.51 (t, J = 4.4 Hz, 1H, NH), 5.33 (t, J = 3.6 Hz, 1H, 12-H), 5.08 (ddd, J = 11.2, 10.3, 4.6 Hz, 1H, 2-H), 4.75 (d, J = 10.3 Hz, 1H, 3-H), 3.39–3.29 (m, 1H, 31-H_a_), 3.18–3.09 (m, 1H, 31-H_b_), 2.56–2.48 (m, 1H, 18-H), 2.36 (t, J = 6.0 Hz, 2H, 32-H), 2.21 (s, 6H, 33-H, 33′-H), 2.05 (s, 3H. Ac), 2.04–1.99 (m, 1H, 1-H_a_), 1.97 (s, 3H, Ac), 1.96–1.87 (m, 3H, 16-H_a_, 11-H_a_, 11-H_b_), 1.81–1.25 (m, 11H, 19-H_a_, 22-H_a_, 16-H_b_, 9-H, 22-H_b_, 15-H_a_, 6-H_a_, 7-H_a_, 6-H_b_, 21-H_a_, 7-H_b_), 1.22–1.12 (m, 2H, 21-H_b_, 19-H_b_), 1.14 (s, 3H, 27-H), 1.05 (s, 3H, 25-H), 1.10–0.99 (m, 2H, 1-H_b_, 15-H_b_), 0.99–0.94 (m, 1H, 5-H), 0.90 (s, 9H, 24-H, 29-H, 30-H), 0.89 (s, 3H, 23-H), 0.79 (s, 3H, 26-H) ppm; ^13^C-NMR (101 MHz, CDCl_3_): δ = 178.1 (C-28), 170.9 (Ac), 170.7 (Ac), 144.7 (C-13), 122.5 (C-12), 80.7 (C-3), 70.2 (C-2), 57.7 (C-32), 55.0 (C-5), 47.6 (C-9), 46.8 (C-19), 46.5 (C-17), 45.4 (C-33, C-33′), 44.1 (C-1), 42.5 (C-18), 42.2 (C-14), 39.6 (C-8), 39.5 (C-4), 38.2 (C-10), 37.0 (C-31), 34.3 (C-21), 33.2 (C-30), 32.8 (C-22), 32.5 (C-7), 30.9 (C-20), 28.6 (C-23), 27.5 (C-15), 25.8 (C-27), 23.8 (C-16), 23.7 (C-11), 23.7 (C-29), 21.3 (Ac), 21.0 (Ac), 18.4 (C-6), 17.8 (C-24), 17.0 (C-26), 16.7 (C-25) ppm; MS (ESI, MeOH): m/z = 627 (100%, [M + H]^+^); analysis calcd. for C_38_H_62_N_2_O_5_ (626.92): C 72.80, H 9.97, N 4.47; found: C 72.63, H 10.14, N4.29.

*(3*β*)-N-[2-(Dimethylamino)-ethyl]-3-hydroxy-olean-12-en-28-amide* (**27**), Compound **27** was prepared from **22** according to general procedure C. Column chromatography (SiO_2_, CHCl_3_/MeOH 95:5) gave **27** (yield: 85%); m.p. 187–190 °C; [α]_D_ = +42.6° (c 0.320, MeOH); R_f_ = 0.29 (CHCl_3_/MeOH 9:1); IR (ATR): ν = 3383br w, 2943m; 1624m, 1532m, 1431s, 1387s, 1319s, 1186m, 1032m, 996m, 823m, 752s cm^−1^; ^1^H-NMR (500 MHz, CDCl_3_): δ = 6.73 (t, J = 5.2 Hz, 1H, NH), 5.40 (t, J = 3.6 Hz, 1H, 12-H), 3.63–3.53 (m, 1H, 31-H_a_), 3.35–3.27 (m, 1H, 31-H_b_), 3.21 (dd, J = 11.4, 4.3 Hz, 1H, 3-H), 2.87–2.81 (m, 2H, 32-H), 2.64–2.57 (m, 1H, 18-H), 2.60 (s, 6H, 33-H, 33′-H), 1.99 (ddd, J = 13.7, 13.6, 3.9 Hz, 1H, 16-H_a_), 1.93–1.89 (m, 2H, 11-H_a_, 11-H_b_), 1.73 (t, J = 13.4 Hz, 1H, 19-H_a_), 1.68–1.30 (m, 12H, 22-H_a_, 16-H_b_, 1-H_a_, 2-H_a_, 2-H_b_, 9-H, 22-H_b_, 6-H_a_, 15-H_a_, 7-H_a_, 6-H_b_, 21-H_a_), 1.26 (ddd, J = 12.3, 3.0, 2.9 Hz, 1H, 7-H_b_), 1.22–1.14 (m, 2H, 21-H_b_, 19-H_b_), 1.15 (s, 3H, 27-H), 1.05 (ddd, J = 14.1, 3.6, 3.5 Hz, 1H, 15-H_b_), 0.98 (s, 3H, 23-H), 0.98–0.93 (m, 1H, 1-H_b_), 0.92 (s, 3H, 29-H), 0.90 (s, 3H, 25-H), 0.90 (s, 3H, 30-H), 0.78 (s, 3H, 24-H), 0.75 (s, 3H, 26-H), 0.74–0.71 (m, 1H, 5-H) ppm; ^13^C-NMR (126 MHz, CDCl_3_): δ = 179.2 (C-28), 144.2 (C-13), 123.2 (C-12), 79.1 (C-3), 57.8 (C-32), 55.3 (C-5), 47.7 (C-9), 46.7 (C-19), 46.6 (C-17), 44.7 (C-33, C-33′), 42.1 (C-14), 42.0 (C-18), 39.6 (C-8), 38.9 (C-4), 38.6 (C-1), 37.1 (C-10), 36.0 (C-31), 34.3 (C-21), 33.2 (C-30), 32.9 (C-22), 32.6 (C-7), 30.8 (C-20), 28.2 (C-23), 27.5 (C-15), 27.3 (C-2), 25.9 (C-27), 23.7 (C-16), 23.7 (C-29), 23.6 (C-11), 18.5 (C-6), 17.2 (C-26), 15.7 (C-24), 15.5 (C-25) ppm; MS (ESI, MeOH): m/z = 527.4 (100%, [M + H]^+^); analysis calcd. for C_34_H_58_N_2_O_2_ (526.85): C 77.51, H 11.10, N 5.32; found: C 77.37, H 11.31, N 5.16.

*(2*α*,3*β*)-N-[2-(Dimethylamino)-ethyl]-2,3-dihydroxy-olean-12-en-28-amide* (**28**), Compound **28** was prepared from **23** according to general procedure C. Column chromatography (SiO_2_, CHCl_3_/MeOH 9:1) gave **28** (yield: 66%); m.p. 146–149 °C; [α]_D_ = +43.1° (c 0.355, CHCl_3_); R_f_ = 0.10 (CHCl_3_/MeOH 95:5); IR (KBr): ν = 3418br s, 2946s, 2862s, 2826m, 2778m, 1636s, 1522m, 1462s, 1386m, 1364m, 1266w, 1188w, 1158w, 1098w, 1050s cm^−1^; ^1^H-NMR (400 MHz, CDCl_3_): δ = 6.56 (t, J = 4.9 Hz, 1H, NH), 5.35 (t, J = 3.6 Hz, 1H, 12-H), 3.68 (ddd, J = 11.3, 9.5, 4.5 Hz, 1H, 2-H), 3.42–3.32 (m, 1H, 31-H_a_), 3.20–3.11 (m, 1H, 31-H_b_), 2.99 (d, J = 9.5 Hz, 1H, 3-H), 2.57–2.50 (m, 1H, 18-H), 2.40 (t, J = 6.1 Hz, 2H, 32-H), 2.25 (s, 6H, 33-H, 33‘-H), 2.01–1.90 (m, 4H, 1-H_a_, 16-H_a_, 11-H_a_, 11-H_b_), 1.80–1.24 (m, 11H, 19-H_a_, 22-H_a_, 16-H_b_, 9-H, 22-H_b_, 15-H_a_, 6-H_a_, 7-H_a_, 6-H_b_, 21-H_a_, 7-H_b_), 1.22–1.16 (m, 2H, 19-H_b_, 21-H_b_), 1.15 (s, 3H, 27-H), 1.06–0.99 (m, 1H, 15-H_b_), 1.02 (s, 3H, 23-H), 0.97 (s, 3H, 25-H), 0.95–0.87 (m, 1H, 1-H_b_), 0.90 (s, 6H, 29-H, 30-H), 0.86–0.82 (m, 1H, 5-H), 0.82 (s, 3H, 24-H), 0.78 (s, 3H, 26-H) ppm; ^13^C-NMR (101 MHz, CDCl_3_): δ = 178.3 (C-28), 144.6 (C-13), 122.8 (C-12), 84.0 (C-3), 67.0 (C-2), 57.8 (C-32), 55.4 (C-5), 47.7 (C-9), 46.8 (C-19), 46.6 (C-1), 46.5 (C-17), 45.3 (C-33, C-33‘), 42.4 (C-18), 42.2 (C-14), 39.6 (C-8), 39.3 (C-4), 38.3 (C-10), 36.9 (C-31), 34.3 (C-21), 33.2 (C-30), 32.8 (C-22), 32.6 (C-7), 30.9 (C-20), 28.8 (C-23), 27.5 (C-15), 25.9 (C-27), 23.8 (C-16), 23.7 (C-11), 23.7 (C-29), 18.5 (C-6), 17.1 (C-24), 16.9 (C-26), 16.8 (C-25) ppm; MS (ESI, MeOH): m/z = 543 (100%, [M + H]^+^); analysis calcd. for C_34_H_58_N_2_O_3_ (542.85): C 75.23, H 10.77, N 5.16; found: C 74.99, H 10.97, N 5.08.

*(3*β*)-N-(2-Pyrrolidin-1-ylethyl)-3-acetyloxy-olean-12-en-28-amide* (**32**), Compound **32** was prepared from **7** according to general procedure B using 1-(2-aminoethyl)-pyrrolidine as amino compound. Column chromatography (SiO_2_, CHCl_3_/MeOH 95:5) gave **32** (yield: 90%); m.p. 175–178 °C (decomp.), lit.: 258 °C (decomp.)[29]; [α]_D_ = +47.9° (c 0.315, CHCl_3_), Lit.: +49.8° (c 0.350, CHCl_3_)[29]; R_f_ = 0.39 (CHCl_3_/MeOH 9:1); IR (ATR): ν = 2944m, 1731m, 1644w, 1522w, 1365m, 1244s, 1027m, 985m, 752m cm^−1^; ^1^H-NMR (400 MHz, CDCl_3_): δ = 6.89 (t, J = 5.6 Hz, 1H, NH), 5.41 (t, J = 3.6 Hz, 1H, 12-H), 4.48 (dd, J = 10.3, 5.7 Hz, 1H, 3-H), 3.90–3.76 (m, 2H, 33-H_a_, 33′-H_a_), 3.73–3.64 (m, 1H, 31-H_a_), 3.51–3.41 (m, 1H, 31-H_b_), 3.35–3.18 (m, 2H, 32-H), 2.99–2.86 (m, 2H, 33-H_b_, 33′-H_b_), 2.64 (dd, J = 13.5, 4.6 Hz, 1H, 18-H), 2.22–1.95 (m, 5H, 34-H, 34′-H, 16-H_a_), 2.03 (s, 3H, Ac), 1.94–1.76 (m, 2H, 11-H_a_, 11-H_b_), 1.70 (t, J = 13.5 Hz, 1H, 19-H_a_), 1.65–1.28 (m, 12H, 1-H_a_, 2-H_a_, 2-H_b_, 7-H_a_, 7-H_b_, 16-H_b_, 9-H, 6-H_a_, 15-H_a_, 22-H_a_, 6-H_b_, 21-H_a_), 1.28–1.15 (m, 3H, 22-H_b_, 19-H_b_, 21-H_b_), 1.13 (s, 3H, 27-H), 1.09–0.97 (m, 2H, 1-H_b_, 15-H_b_), 0.92 (s, 3H, 25-H), 0.91 (s, 3H, 29-H), 0.89 (s, 3H, 30-H), 0.85 (s, 3H, 23-H), 0.84 (s, 3H, 24-H), 0.84–0.79 (m, 1H, 5-H), 0.71 (s, 3H, 26-H) ppm; ^13^C-NMR (101 MHz, CDCl_3_): δ = 179.9 (C-28), 171.1 (Ac), 143.9 (C-13), 123.3 (C-12), 81.0 (C-3), 55.3 (C-5), 55.1 (C-32), 54.8 (C-33, C-33′), 47.6 (C-9), 46.5 (C-19), 46.5 (C-17), 41.9 (C-14), 41.5 (C-18), 39.5 (C-8), 38.2 (C-1), 37.8 (C-4), 37.0 (C-10), 36.4 (C-31), 34.2 (C-21), 33.2 (C-30), 32.9 (C-7), 32.5 (C-22), 30.8 (C-20), 28.2 (C-23), 27.5 (C-15), 25.9 (C-27), 23.6 (C-2), 23.6 (C-29), 23.6 (C-11), 23.6 (C-16), 23.4 (C-34, C-34′), 21.4 (Ac), 18.3 (C-6), 17.2 (C-26), 16.8 (C-24), 15.5 (C-25) ppm; MS (ESI, MeOH): m/z = 595.5 (100%, [M + H]^+^); analysis calcd. for C_38_H_62_N_2_O_3_ (594.93): C 76.72, H 10.50, N 4.71; found: C 76.51, H 10.73, N 4.69.

*(2*α*,3*β*)-N-(2-Pyrrolidin-1-ylethyl)-2,3-diacetyloxy-olean-12-en-28-amide* (**33**), Compound **33** was prepared from **8** according to general procedure B using 1-(2-aminoethyl)-pyrrolidine as amino compound. Column chromatography (SiO_2_, CHCl_3_/MeOH 9:1) gave **33** (yield: 96%); m.p. 143–146 °C (decomp.); [α]_D_ = +20.0° (c 0.345, CHCl_3_); R_f_ = 0.30 (CHCl_3_/MeOH 95:5); IR (KBr): ν = 3426br s, 2948s, 2802w, 1744s, 1654m, 1508m, 1460m, 1368m, 1250s, 1152w, 1044m cm^−1^; ^1^H-NMR (400 MHz, CDCl_3_): δ = 6.58 (t, J = 4.3 Hz, 1H, NH), 5.30 (t, J = 3.6 Hz, 1H, 12-H), 5.08 (ddd, J = 11.5, 10.3, 4.7 Hz, 1H, 2-H), 4.74 (d, J = 10.3 Hz, 1H, 3-H), 3.46–3.37 (m, 1H, 31-H_a_), 3.24–3.13 (m, 1H, 31-H_b_), 2.67–2.44 (m, 7H, 32-H, 33-H, 33′-H, 18-H), 2.04 (s, 3H, Ac), 2.03–1.98 (m, 1H, 1-H_a_), 1.97 (s, 3H, Ac), 1.96–1.83 (m, 3H, 11-H_a_, 11-H_b_, 16-H_a_), 1.82–1.76 (m, 4H, 34-H, 34′-H), 1.75–1.23 (m, 11H, 19-H_a_, 22-H_a_, 16-H_b_, 9-H, 22-H_b_, 15-H_a_, 6-H_a_, 7-H_a_, 6-H_b_, 21-H_a_, 7-H_b_), 1.24–1.10 (m, 2H, 21-H_b_, 19-H_b_), 1.14 (s, 3H, 27-H), 1.05 (s, 3H, 25-H), 1.09–0.99 (m, 2H, 1-H_b_, 15-H_b_), 0.98–0.93 (m, 1H, 5-H), 0.90 (s, 9H, 24-H, 29-H, 30-H), 0.89 (s, 3H, 23-H), 0.77 (s, 3H, 26-H) ppm; ^13^C-NMR (101 MHz, CDCl_3_): δ = 177.9 (C-28), 170.8 (Ac), 170.5 (Ac), 144.6 (C-13), 122.3 (C-12), 80.5 (C-3), 70.0 (C-2), 54.8 (C-5), 54.2 (C-32), 53.9 (C-33, C-33′), 47.5 (C-9), 46.7 (C-19), 46.3 (C-17), 43.9 (C-1), 42.3 (C-18), 42.0 (C-14), 39.4 (C-8), 39.3 (C-4), 38.1 (C-10), 37.8 (C-31), 34.2 (C-21), 33.0 (C-30), 32.6 (C-22), 32.3 (C-7), 30.7 (C-20), 28.4 (C-23), 27.3 (C-15), 25.6 (C-27), 23.7 (C-11), 23.7 (C-34, C-34′), 23.6 (C-16), 23.5 (C-29), 21.1 (Ac), 20.9 (Ac), 18.2 (C-6), 17.6 (C-24), 16.8 (C-26), 16.5 (C-25) ppm; MS (ESI, MeOH): m/z = 653 (100%, [M + H]^+^); analysis calcd. for C_40_H_64_N_2_O_5_ (652.96): C 73.58, H 9.88, N 4.29; found: C 73.27, H 10.02, N 4.02.

*(3*β*)-N-(2-Pyrrolidin-1-ylethyl)-3-hydroxy-olean-12-en-28-amide* (**37**), Compound **37** was prepared from **32** according to general procedure C. Column chromatography (SiO_2_, CHCl_3_/MeOH 95:5) gave **37** (yield: 76%); m.p. 189–192 °C; [α]_D_ = +40.8° (c 0.325, MeOH); R_f_ = 0.28 (CHCl_3_/MeOH 9:1); IR (ATR): ν = 3386br w, 2941m, 1631m, 1527m, 1320s, 1031m, 996m, 823m, 752m cm^−1^; ^1^H-NMR (500 MHz, CDCl_3_): δ = 6.94 (t, J = 5.6 Hz, 1H, NH), 5.42 (t, J = 3.7 Hz, 1H, 12-H), 3.88–3.78 (m, 2H, 33-H_a_, 33′-H_a_), 3.73–3.62 (m, 1H, 31-H_a_), 3.51–3.41 (m, 1H, 31-H_b_), 3.35–3.22 (m, 2H, 33-H_b_, 33′-H_b_), 3.20 (dd, J = 11.5, 4.4 Hz, 1H, 3-H), 2.99–2.88 (m, 2H, 32-H), 2.66 (dd, J = 12.9, 3.7 Hz, 1H, 18-H), 2.19–2.05 (m, 4H, 34-H, 34′-H), 2.01 (ddd, J = 13.9, 13.8, 3.9 Hz, 1H, 16-H_a_), 1.97–1.84 (m, 2H, 11-H_a_, 11-H_b_), 1.70 (t, J = 13.4 Hz, 1H, 19-H_a_), 1.65–1.29 (m, 12H, 1-H_a_, 16-H_b_, 2-H_a_, 2-H_b_, 22-H_a_, 22-H_b_, 9-H, 6-H_a_, 15-H_a_, 7-H_a_, 6-H_b_, 21-H_a_), 1.28–1.23 (m, 1H, 7-H_b_), 1.21–1.15 (m, 2H, 19-H_b_, 21-H_b_), 1.14 (s, 3H, 27-H), 1.04 (ddd, J = 14.4, 3.5, 3.4 Hz, 1H, 15-H_b_), 0.98 (s, 3H, 23-H), 0.97–0.92 (m, 1H, 1-H_b_), 0.91 (s, 3H, 29-H), 0.90 (s, 3H, 25-H), 0.89 (s, 3H, 30-H), 0.77 (s, 3H, 24-H), 0.74–0.70 (m, 1H, 5-H), 0.71 (s, 3H, 26-H) ppm; ^13^C-NMR (126 MHz, CDCl_3_): δ = 180.1 (C-28), 143.8 (C-13), 123.4 (C-12), 79.1 (C-3), 55.3 (C-5), 55.1 (C-33, C-33′), 54.9 (C-32), 47.7 (C-9), 46.5 (C-17, C-19), 41.9 (C-14), 41.5 (C-18), 39.6 (C-8), 38.9 (C-4), 38.6 (C-1), 37.1 (C-10), 36.4 (C-31), 34.3 (C-21), 33.2 (C-30), 32.9 (C-22), 32.6 (C-7), 30.8 (C-20), 28.2 (C-23), 27.5 (C-15), 27.3 (C-2), 26.0 (C-27), 23.7 (C-29), 23.6 (C-16), 23.5 (C-11), 23.4 (C-34, C-34′), 18.5 (C-6), 17.2 (C-26), 15.7 (C-24), 15.5 (C-25) ppm; MS (ESI, MeOH): m/z = 553.5 (100%, [M + H]^+^); analysis calcd. for C_36_H_60_N_2_O_2_ (552.89): C 78.21, H 10.94, N 5.07; found: C 78.01, H 11.13, N 4.83.

*(2*α*,3*β*)-N-(2-Pyrrolidin-1-ylethyl)-2,3-dihydroxy-olean-12-en-28-amide* (**38**), Compound **38** was prepared from **33** according to general procedure C. Column chromatography (SiO_2_, CHCl_3_/MeOH 9:1) gave **38** (yield: 67%); m.p. 153–156 °C (decomp.); [α]_D_ = +44.3° (c 0.330, CHCl_3_); R_f_ = 0.10 (CHCl_3_/MeOH 95:5); IR (KBr): ν = 3406br s, 2946s, 2878s, 2808m, 1636s, 1522m, 1462m, 1386m, 1364m, 1268w, 1194w, 1150w, 1050m cm^−1^; ^1^H-NMR (400 MHz, CDCl_3_): δ = 6.72 (t, J = 5.1 Hz, 1H, NH), 5.34 (t, J = 3.6 Hz, 1H, 12-H), 3.67 (ddd, J = 11.3, 9.4, 4.4 Hz, 1H, 2-H), 3.53–3.43 (m, 1H, 31-H_a_), 3.31–3.22 (m, 1H, 31-H_b_), 2.99 (d, J = 9.5 Hz, 1H, 3-H), 2.80–2.63 (m, 6H, 32-H, 33-H, 33′-H), 2.56 (dd, J = 13.4, 4.3 Hz, 1H, 18-H), 2.02–1.89 (m, 4H, 1-H_a_, 11-H_a_, 11-H_b_, 16-H_a_), 1.89–1.83 (m, 4H, 34-H, 34′-H), 1.79–1.24 (m, 11H, 19-H_a_, 22-H_a_, 16-H_b_, 9-H, 22-H_b_, 6-H_a_, 15-H_a_, 7-H_a_, 6-H_b_, 21-H_a_, 7-H_b_), 1.23–1.11 (m, 2H, 21-H_b_, 19-H_b_), 1.14 (s, 3H, 27-H), 1.08–0.99 (m, 1H, 15-H_b_), 1.02 (s, 3H, 23-H), 0.97 (s, 3H, 25-H), 0.94–0.87 (m, 1H, 1-H_b_), 0.90 (s, 3H, 29-H), 0.89 (s, 3H, 30-H), 0.85–0.79 (m, 1H, 5-H), 0.81 (s, 3H, 24-H), 0.75 (s, 3H, 26-H) ppm; ^13^C-NMR (101 MHz, CDCl_3_): δ = 178.6 (C-28), 144.5 (C-13), 122.8 (C-12), 84.0 (C-3), 68.9 (C-2), 55.3 (C-5), 54.6 (C-32), 54.2 (C-33, C-33′), 47.7 (C-9), 46.8 (C-19), 46.5 (C-1), 46.5 (C-17), 42.2 (C-18), 42.1 (C-14), 39.6 (C-8), 39.3 (C-4), 38.3 (C-10), 37.6 (C-31), 34.3 (C-21), 33.2 (C-30), 32.8 (C-22), 32.5 (C-7), 30.8 (C-20), 28.8 (C-23), 27.5 (C-15), 25.9 (C-27), 23.8 (C-11), 23.7 (C-29), 23.7 (C-34, C-34′), 23.6 (C-16), 18.5 (C-6), 17.1 (C-26), 16.9 (C-24), 16.8 (C-25) ppm; MS (ESI, MeOH): m/z = 569 (100%, [M + H]^+^); analysis calcd. for C_36_H_60_N_2_O_3_ (568.89): C 76.01, H 10.63, N 4.92; found: C 75.86, H 10.90, N 4.77.

*(3*β*)-N-(2-Piperidin-1-ylethyl)-3-acetyloxy-olean-12-en-28-amide* (**42**), Compound **42** was prepared from **7** according to general procedure B using 1-(2-aminoethyl)-piperidine as amino compound. Column chromatography (SiO_2_, CHCl_3_/MeOH 9:1) gave **42** (yield: 76%); m.p. = 163–166 °C (decomp.); [α]_D_ = +47.5° (c 0.305, CHCl_3_); R_f_ = 0.55 (silica gel, CHCl_3_/MeOH 9:1); IR (ATR): ν = 2943m, 2864w, 1732m, 1642w, 1523w, 1432m, 1388m, 1365s, 1330m, 1244vs, 1214m, 1149w, 1097w, 1027m, 1007m, 986m, 751m; ^1^H-NMR (500 MHz, CDCl_3_): δ = 6.93 (t, J = 5.3 Hz, 1H, NH), 5.42 (dd, J = 3.3 Hz, 3.3 Hz, 1H, 12-H), 4.51–4.44 (m, 1H, 3-H), 3.71 (dq, J = 11.9, 5.9 Hz, 1H, 31-H_a_), 3.46 (dq, J = 12.2, 6.0 Hz, 1H, 31-H_b_), 3.13 (s, 2H, 32-H), 2.64 (dd, J = 12.9, 3.3 Hz, 1H, 18-H), 2.03 (s, 3H, Ac), 2.02–1.84 (m, 8H, 2-H_a_, 2-H_b_, 16-H_a_, 16-H_b_. 33-H, 33′-H), 1.70 (m, 1H, 19-H_a_), 1.66–1.40 (m, 15H, 1-H_a_, 11-H_a_, 22-H_a_, 11-H_b_, 9-H, 22-H_b_, 6-H_a_, 15-H_a_, 7-H_a_, 34-H, 34′-H, 35-H), 1.40–1.30 (m, 2H, 6-H_b_, 21-H_a_), 1.24 (m, 1H, 7-H_b_), 1.22–1.14 (m, 2H, 19-H_b_, 21-H_b_), 1.13 (s, 3H, 27-H), 1.04 (m, 2H, 1-H_b_, 15-H_b_), 0.92 (s, 3H, 29-H) 0.92 (s, 3H, 26-H), 0.89 (s, 3H, 30-H), 0.85 (s, 3H, 23-H), 0.84 (s, 3H, 25-H), 0.81 (s, 1H, 5-H), 0.71 (s, 3H, 24-H); ^13^C-NMR (126 MHz, CDCl_3_): δ = 179.9 (C-28), 171.1 (Ac), 143.9 (C-13), 123.3 (C-12), 81.0 (C-3), 56.9 (C-32), 55.3 (C-5), 54.5 (C-33 + C-33′), 47.6 (C-9), 46.5 (C-19), 46.5 (C-17), 41.9 (C-14), 41.5 (C-18), 39.5 (C-8), 38.2 (C-1), 37.8 (C-4), 37.0 (C-10), 34.9 (C-31), 34.3 (C-21), 33.2 (C-30), 32.9 (C-22), 32.5 (C-7), 30.8 (C-20), 28.2 (C-23), 27.5 (C-15), 26.0 (C-27), 23.6 (C-2), 23.6 (C-29), 23.6 (C-11), 23.6 (C-16), 23.1 (C-34 + C-34‘), 22.1 (C-35), 21.4 (Ac), 18.3 (C-6), 17.2 (C-24), 16.8 (C-25), 15.5 (C-26); MS (ESI, MeOH): m/z = 609.5 (100%, [M + H]^+^); analysis calcd. for C_39_H_64_N_2_O_3_ (608.95): C 76.92, H 10.59, N 4.60; found: C 76.78, H 10.79, N 4.44.

*(2*α*,3*β*)-N-(2-Piperidin-1-ylethyl)-2,3-diacetyloxy-olean-12-en-28-amide* (**43**), Compound **43** was prepared from **8** according to general procedure B using 1-(2-aminoethyl)-piperidine as amino compound. Column chromatography (SiO_2_, CHCl_3_/MeOH 9:1) gave **43** (yield: 71%); m.p. = 130–133 °C (decomp.); [α]_D_ = +15.7° (c 0.35, CHCl_3_); R_f_ = 0.47 (silica gel, CHCl_3_/MeOH 9:1); IR (ATR): ν = 2938m, 2863w, 1741s, 1653m, 1508m, 1503m, 1466m, 1455m, 1443w, 1434w, 1367m, 1303w, 1247vs, 1229vs, 1155w, 1127w, 1041s, 1032s; ^1^H-NMR (500 MHz, CDCl_3_) δ = 6.69 (s, 1H, NH), 5.36 (dd, J = 3.4 Hz, 1H, 12-H), 5.08 (td, J = 11.4, 4.6 Hz, 1H, 2-H), 4.74 (d, J = 10.4 Hz, 1H, 3-H), 3.40 (m, 1H, 31-H_a_), 3.20 (m, 1H, 31-H_b_), 2.55 (d, J = 11.7 Hz, 1H, 18-H), 2.43 (s, 6 H, 32-H_a_, 32-H_b_, 33-H, 33‘-H), 2.05 (s, 3H, Ac), 1.97 (s, 3H, Ac), 2.07–1.82 (m, 3H, 1-H_a_, 16-H_a_, 16-H_b_), 1.78–1.42 (m, 15H, 19-H_a_, 11-H_a_, 22-H_a_, 9-H, 11-H_b_, 34-H, 34‘-H, 6-H_a_, 15-H_a_, 22-H_b_, 35-H, 7-H_a_), 1.42–1.13 (m, 5H, 6-H_b_, 21-H_a_, 7-H_b_, 21-H_b_, 19-H_b_), 1.14 (s, 3H, 27-H), 1.04 (s, 3H, 25-H), 1.09–0.92 (m, 3H, 1-H_b_, 15-H_b_, 5-H), 0.90 (s, 3H, 29-H), 0.90 (s, 3H, 24-H), 0.90 (s, 3H, 30-H), 0.90 (s, 3H, 23-H), 0.75 (s, 3H, 26-H); ^13^C-NMR (126 MHz, CDCl_3_) δ = 178.0, (C-28),170.9 (Ac), 170.7 (Ac), 144.8 (C-13), 122.4 (C-12), 80.7 (C-3), 70.2 (C-2), 57.0 (C-32), 55.0 (C-33 + C-33′), 54.4 (C-5), 47.6 (C-9), 46.9 (C-19), 46.4 (C-17), 44.0 (C-1), 42.3 (C-18), 42.1 (C-14), 39.6 (C-8), 39.5 (C-4), 38.3 (C-10), 35.8 (C-31), 34.3 (C-21), 33.2 (C-30), 32.8 (C-22), 32.3 (C-7), 30.9 (C-20), 28.6 (C-23), 27.4 (C-15), 25.8 (C-38 + C-38′), 25.9 (C-27), 24.2 (C-35), 23.7 (C-16 + C-11), 23.7 (C-29), 21.3 (Ac), 21.0 (Ac), 18.3 (C-6), 17.8 (C-24), 17.0 (C-26), 16.6 (C-25); MS (ESI, MeOH): m/z = 667.5 ([M + H]^+^, 100%); analysis calcd. for C_41_H_66_N_2_O_5_ (666.99): C 73.83, H 9.97, N 4.20; found: C 73.69, H 10.18, N 4.01.

*(3*β*)-N-(2-Piperidin-1-ylethyl)-3-acetyloxy-20-oxo-30-norlupan-28-amide* (**45**), Compound **45** was prepared from **10** according to general procedure B using 1-(2-aminoethyl)-piperidine as amino compound. Column chromatography (SiO_2_, CHCl_3_/MeOH 9:1) gave **45** (yield: 63%); m.p. = 141–144 °C; [α]_D_ = –9.5° (c 0.34, CHCl_3_); R_f_ = 0.43 (silica gel, CHCl_3_/MeOH); IR (ATR): ν = 2939m, 2867w, 1733m, 1711m, 1657m, 1654m, 1517m, 1468m, 1451m, 1391w, 1367m, 1352m, 1316w, 1302w, 1243vs, 1196m, 1157m, 1130w, 1110w, 1090w, 1073w, 1026m, 979m; ^1^H-NMR (500 MHz, CDCl_3_) δ = 6.76 (s, 1H, NH), 4.45 (dd, J = 11.0, 5.1 Hz, 1H, 3-H), 3.40 (m, 4H, 18-H, 31-H_a_, 31-H_b_), 2.63–2.52 (m, 6H, 32-H_a_, 32-H_b_, 33-H, 33′-H), 2.21–2.14 (m, 1H, 13-H), 2.15 (s, 3H, 29-H), 2.09–2.00 (m, 3H, 9-H, 16-H_a_, 21-H_a_), 2.02 (s, 3H, Ac), 1.84–1.78 (dd, 1H, 1-H_a_), 1.71–1.53 (m, 8H, 34-H, 34′-H, 19-H_a_, 1-H_b_, 16-H_b_, 2-H_a_), 1.53–1.20 (m, 14H, 13-H_a_, 2-H_b_, 21-H_b_, 6-H_a_, 35-H, 11-H_a_, 12-H_a_, 7-H_a_, 7-H_b_, 6-H_b_, 11-H_b_, 22-H_a_, 22-H_b_), 1.20–1.13 (m, 1H, 12-H_b_), 1.10–1.00 (m, 2H, 15-H_a_, 15-H_b_), 0.98 (s, 3H, 27-H), 0.96–0.91 (m, 1H, 19-H_b_), 0.88 (s, 3H, 26-H), 0.82 (s, 3H, 23-H), 0.82 (s, 3H, 25-H), 0.81 (s, 3H, 26-H), 0.79–0.75 (m, 1H, 5-H); ^13^C-NMR (126 MHz, CDCl_3_) δ = 212.9 (C-20), 176.5 (Ac), 171.0 (C-28), 81.0 (C-3), 57.3 (C-32), 55.8 (C-5), 55.5 (C-17), 54.3 (C-33), 51.4 (C-18), 50.5 (C-9), 50.1 (C-19), 42.4 (C-14), 40.8 (C-8), 38.5 (C-1), 38.0 (C-4), 37.9 (C-10), 37.2 (C-13), 37.0 (C-22), 35.3 (C-31), 34.4 (C-7), 32.9 (C-16), 30.3 (C-29), 29.6 (C-12), 28.8 (C-21), 28.1 (C-23), 27.3 (C-15), 25.3 (C-34), 23.8 (C-35), 23.8 (C-2), 21.4 (Ac), 21.1 (C-11), 18.3 (C-6), 16.6 (C-24), 16.3 (C-25), 16.3 (C-26), 14.8 (C-27); MS (ESI, MeOH): m/z = 611.5 (100%, [M + H]^+^); analysis calcd. for C_38_H_62_N_2_O_4_ (610.92): C 74.71, H 10.23, N 4.59; found: C 74.56, H 10.51, N 4.39.

*(3*β*)-N-(2-Piperidin-1-ylethyl)-3-hydroxy-olean-12-en-28-amide* (**47**), Compound **47** was prepared from **42** according to general procedure C. Column chromatography (SiO_2_, CHCl_3_/MeOH 95:5) gave **47** (yield: 93%); m.p. = 184–186 °C (decomp.); [α]_D_ = +49.5° (c 0.365, CHCl_3_); R_f_ = 0.26 (silica gel, CHCl_3_/MeOH 9:1); IR (ATR): ν = 3372w, 2941m, 2864m, 1633m, 1527m, 1431s, 1387s, 1378s, 1357s, 1324vs, 1245m, 1212m, 1200w, 1188w, 1138w, 1093w, 1032m, 1006m, 997m, 752m; ^1^H-NMR (500 MHz, CDCl_3_) δ = 6.97 (t, J = 5.3 Hz, 1H, NH), 5.43 (dd, J = 3.4, 3.4 Hz, 1H, 12-H), 4.58 (s, 1H, OH), 3.71 (dq, J = 11.7, 5.8 Hz, 1H, 31-H_a_), 3.46 (dq, J = 12.2, 5.9 Hz, 1H, 31-H_b_), 3.22–3.17 (m, 1H, 3-H), 3.17–3.11 (m, 2H, 32-H_a_, 32-H_b_), 2.65 (dd, J = 13.4, 3.6 Hz, 2H, 18-H), 2.05–1.95 (m, 2H, 16-H_a_, 16-H_b_), 1.95–1.84 (m, 8H, 11-H_a_, 11-H_b_, 34-H, 34‘-H, 35-H), 1.74–1.67 (m, 1H, 19-H_a_), 1.65–1.45 (m, 10H, 1-H_a_, 16-H_a_, 22-H_a_, 16-H_b_, 22-H_b_, 2-H_a_, 2-H_b_, 9-H, 6-H_a_, 15-H_a_), 1.45–1.39 (m, 1H, 7-H_a_), 1.40–1.29 (m, 2H, 6-H_b_, 21-H_a_), 1.29–1.22 (m, 1H, 7-H_b_), 1.21–1.15 (m, 2H, 19-H_b_, 21-H_b_), 1.14 (s, 3H, 27-H), 1.07–1.00 (m, 1H, 15-H_b_), 0.97 (s, 3H, 23-H), 0.97–0.93 (m, 1H, 1-H_b_), 0.92 (s, 3H, 29-H), 0.89 (s, 3H, 25-H), 0.89 (s, 3H, 30-H), 0.77 (s, 3H, 24-H), 0.73 (s, 1H, 5-H), 0.71 (s, 3H, 26-H); ^13^C-NMR (126 MHz, CDCl_3_) δ = 179.9 (C-28), 143.8 (C-13), 123.4 (C-12), 79.1 (C-3), 55.3 (C-32), 56.9 (C-5), 54.5 (C-33 + C-33′), 47.7 (C-9), 46.5 (C-19), 46.5 (C-17), 41.9 (C-14), 41.5 (C-18), 39.5 (C-8), 38.9 (C-4), 38.6 (C-1), 37.1 (C-10), 34.9 (C-31), 34.3 (C-21), 33.2 (C-30), 32.9 (C-22), 32.6 (C-7), 30.8 (C-20), 28.2 (C-23), 27.5 (C-15), 27.3 (C-2), 26.0 (C-27), 23.6 (C-29), 23.6 (C-16), 23.6 (C-11), 23.0 (C-34 + C-34′), 22.1 (C-35), 18.4 (C-6), 17.2 (C-26), 15.7 (C-24), 15.5 (C-25); MS (ESI, MeOH): m/z = 567.5 (100%, [M + H]^+^); analysis calcd. for C_37_H_62_N_2_O_2_ (566.92): C 78.39, H 11.02, N 4.94; found: C 78.21,H 11.18, N 4.78.

*(2*α*,3*β*)-N-(2-Piperidin-1-ylethyl)-2,3-dihydroxy-olean-12-en-28-amide* (**48**), Compound **48** was prepared from **43** according to general procedure C. Column chromatography (SiO_2_, CHCl_3_/MeOH 9:1) gave **48** (yield: 87%); m.p. = 149–152 °C; [α]_D_ = +45.2° (c 0.31, CHCl_3_); R_f_ = 0.21 (silica gel, CHCl_3_/MeOH 9:1); IR (ATR): ν = 3378m, 2934vs, 2861m, 2853m, 1635s, 1513s, 1459s, 1454s, 1386s, 1378s, 1364m, 1348m, 1328w, 1303m, 1281m, 1266m, 1259m, 1232w, 1209w, 1194m, 1154m, 1129m, 1111w, 1096m, 1084m, 1050vs, 1016m, 992m, 958m, 660m; ^1^H-NMR (500 MHz, CDCl_3_) δ = 5.41 (s, 1H, 12-H), 3.74–3.64 (m, 1H, 2-H), 3.59–3.45 (m, 1H, 31-H_a_), 3.43–3.25 (m, 1H, 31-H_b_), 3.00 (d, J = 9.5 Hz, 1H, 3-H), 2.72–2.60 (m, 1H, 18-H), 2.64–2.36 (m, 2H, 32-H_a_, 32-H_b_), 2.02–1.92 (m, 4H, 1-H_a_, 16-H_a_, 16-H_b_, 11-H_a_), 1.77–1.49 (m, 17H, 1-H_b_, 33-H, 33‘-H, 9-H, 11-H_b_, 34-H, 34‘-H, 6-H_a_, 22-H_a_, 35-H, 7-H_a_, 15-H_a_), 1.50–1.42 (m, 1H, 7-H_b_), 1.41–1.31 (m, 2H, 6-H_b_, 21-H_a_), 1.31–1.28 (m, 1H, 21-H_b_), 1.19 (m, 3H, 22-H_b_, 19-H_a_), 1.15 (s, 3H, 27-H), 1.04 (m, 1H, 15-H_b_), 1.03 (s, 3H, 23-H), 0.97 (s, 3H, 25-H), 0.93 (s, 3H, 29-H), 0.91 (s, 3H, 30-H), 0.92-0.88 (m, 1H, 19-H_b_), 0.86-0.82 (m, 1H, 5-H), 0.82 (s, 3H, 26-H), 0.74 (s, 3H, 24-H); ^13^C-NMR (126 MHz, CDCl_3_) δ = 179.0 (C-28), 144.0 (C-13), 122.8 (C-12), 84.1 (C-3), 69.1 (C-2), 57.2 (C-32), 55.37 (C-33 + C-33‘), 54.5 (C-5), 48.2 (C-17), 47.7 (C-9), 46.5 (C-19), 46.5 (C-1), 43.2 (C-18), 42.1 (C-14), 39.6 (C-8), 39.3 (C-4), 39.1 (C-10), 38.4 (C-31), 34.3 (C-21), 33.2 (C-30), 33.0 (C-22), 32.5 (C-7), 30.9 (C-20), 28.8 (C-23), 27.5 (C-15), 25.8 (C-34 + C-34‘), 26.0 (C-27), 24.1 (C-35), 23.7 (C-16), 23.7 (C-11), 23.7 (C-29), 18.5 (C-6), 17.2 (C-24), 16.9 (C-26), 16.8 (C-25); MS (ESI, MeOH): m/z = 569.5 ([M + H]^+^, 100%); analysis calcd. for C_37_H_62_N_2_O_3_ (582.91): C 76.24, H 10.72, N 4.81; found: C 75.97, H 10.93, N 4.57.

*(3*β*)-N-(2-Piperidin-1-ylethyl)-3-hydroxy-20-oxo-30-norlupan-28-amide* (**50**), Compound **50** was prepared from **45** according to general procedure C. Column chromatography (SiO_2_, CHCl_3_/MeOH 9:1) gave **50** (yield: 86%); m.p. = 153–156 °C (decomp.); [α]_D_ = –21.2° (c 0.115, CHCl_3_); R_f_ = 0.16 (silica gel, CHCl_3_/MeOH); IR (ATR): ν = 3371w, 2934vs, 2865m, 1706m, 1643s, 1524s, 1519s, 1466s, 1450s, 1387m, 1376s, 1356s, 1328m, 1319m, 1301m, 1277m, 1245s, 1197s, 1161m, 1131m, 1109m, 1085m, 1046s, 1035s, 1003m, 987m, 973m; ^1^H-NMR (500 MHz, CDCl_3_) δ = 3.67–3.55 (m, 2H, 31-H_a_, 31-H_b_), 3.27 (td, J = 11.2, 3.9 Hz, 2H, 1-H_a_, 1-H_b_), 3.17–3.07 (m, 3H, 32-H_a_, 32-H_b_, 3-H), 2.20–2.12 (m, 1H, 13-H), 2.09 (s, 3H, 28-H), 2.10–2.02 (m, 1H, 16-H_a_), 2.00–1.94 (m, 1H, 18-H), 1.91–1.73 (m, 6H, 12-H_a_, 34-H, 34′-H, 22-H_a_), 1.62–1.06 (m, 22H, 19-H_a_, 33-H, 33′-H, 15-H_a_, 15-H_b_, 2-H_a_, 16-H_b_, 6-H_a_, 22-H_b_, 12-H_b_, 11-H_a_, 6-H_b_, 7-H_a_, 35-H, 7-H_b_, 21-H_a_, 9-H, 11-H_b_, 21-H_b_), 1.05–0.92 (m, 1H, 2-H_b_), 0.90 (s, 3H, 27-H), 0.89 (s, 3H, 23-H), 0.82 (s, 3H, 25-H), 0.85–0.78 (m, 1H, 19-H_b_), 0.74 (s, 3H, 24-H), 0.68 (s, 3H, 26-H), 0.63–0.58 (m, 1H, 5-H); ^13^C-NMR (126 MHz, CDCl_3_) δ = 212.7 (C-20), 177.8 (C-30), 78.7 (C-3), 56.9 (C-32), 55.6 (C-17), 55.3 (C-5), 54.0 (C-33), 51.1 (C-19), 50.4 (C-9), 50.0 (C-18), 42.1 (C-14), 40.6 (C-8), 38.8 (C-4), 38.6 (C-1), 37.6 (C-22), 37.1 (C-10), 36.7 (C-13), 34.3 (C-7), 34.2 (C-31), 32.1 (C-16), 30.0 (C-28), 29.4 (C-21), 28.4 (C-12), 28.0 (C-23), 27.3 (C-15), 27.2 (C-2), 22.8 (C-34), 21.8 (C-35), 20.90 (C-11), 18.2 (C-6), 16.1 (C-24), 16.1 (C-25), 15.4 (C-26), 14.6 (C-27); MS (ESI, MeOH): m/z = 568.5 (100%, [M + H]^+^); analysis calcd. for C_36_H_60_N_2_O_3_ (568.89): C 76.01, H 10.63, N 4.92; found: C 75.93, H 10.46, N 4.71.

## 5. Conclusions

In this study, 40 triterpenoic acid amides (**11**–**50**), with different triterpenoic backbones and various amine residues were synthesized and subjected to Ellman’s assay to determine their potential as inhibitors of cholinesterases. Furthermore, some enzyme kinetic studies were performed. Thus, systematic variation of the amine substituent led to analogs possessing the same or even better BChE-inhibiting properties as standard galantamine hydrobromide. Outstanding derivatives were 2-pyrrolidin-1-ylethyl substituted compounds **34** (from **BA**), **36** (from **UA**), and **37** (from **OA**), showing K_i_ values of 0.39 ± 0.04 µM, 0.47 ± 0.08 µM, and 0.55 ± 0.02 µM, respectively. Furthermore, Ellman’s assay revealed several platanic acid compounds as excellent inhibitors of BChE. Particularly, **40**, **45**, and **50** were great inhibitors, showing inhibition rates even in the nanomolar range. The most active compound in the test was a hybrid holding a platanic acid backbone and a pyrrolidinyl residue. For (3β)-*N*-(2-pyrrolidin-1-ylethyl)-3-acetyloxy-20-oxo-30- norlupan-28-amide (**35**), inhibition constants K_i_ = 0.07 ± 0.01 µM and K_i_′ = 2.38 ± 0.48 µM have been determined. The results obtained in the biological assay can be explained by appropriate molecular modeling calculations. All active compounds were mixed-type BChE inhibitors with a dominating competitive part (K_i_ < K_i_′). The best inhibitor for acetylcholinesterase was a betulinic acid derived piperidinyl derivative (49), acting as a mixed-type inhibitor showing K_i_ = 1.00 ± 0.09 µM and K_i_′ = 1.42 ± 0.08 μM, respectively.

## Figures and Tables

**Table 1:** Significant results of the BChE inhibition assay. Inhibitory constants (K_i_ and K_i_′ in µM), determined using Ellman’s assays employing butyrylcholinesterase (BChE, equine serum) with galantamine hydrobromide (**GH**) as standard.

Compound	K_i_ in µM/K_i_′ in µM (Type of Inhibition)	Compound	K_i_ in µM/K_i_′ in µM (Type of Inhibition)
**GH**	2.30 ± 0.17 (competitive)	**32**	2.02 ± 0.56/29.88 ± 1.13 (mixed-type)
**16**	5.55 ± 0.12/12.15 ± 0.45 (mixed-type)	**34**	0.39 ± 0.04/> 5 (mixed-type)
**17**	1.93 ± 0.13/7.68 ± 0.61 (mixed-type)	**36**	0.47 ± 0.08/2.23 ± 0.12 (mixed-type)
**21**	6.48 ± 0.10/143.36 ± 0.02 (mixed-type)	**37**	0.55 ± 0.02/> 50 (mixed-type)
**24**	1.93 ± 0.13/7.68 ± 0.61 (mixed-type)	**38**	2.06 ± 0.03/14.15 ± 2.42 (mixed-type)
**26**	1.11 ± 0.09/3.10 ± 0.30 (mixed-type)	**39**	3.24 ± 0.58/> 9 (mixed-type)
**27**	1.31 ± 0.01/9.07 ± 0.02 (mixed-type)	**46**	2.37 ± 0.02/7.64 ± 0.22 (mixed-type)
**28**	2.20 ± 0.25/27.26 ± 3.47 (mixed-type)	**47**	1.57 ± 0.01/10.42 ± 1.36 (mixed-type)
**29**	3.52 ± 0.07/16.11 ± 0.18 (mixed-type)	**48**	2.26 ± 0.05/6.21 ± 0.47 (mixed-type)
**31**	4.42 ± 0.34/6.00 ± 0.47 (mixed-type)	**49**	2.52 ± 0.29/> 8 (mixed-type)

**Table 2:** Results of the BChE inhibition assay for platanic acid derivatives. Inhibitory constants (K_i_ and K_i_′ in µM), determined using Ellman’s assay employing butyrylcholinesterase (BChE, equine serum) with galantamine hydrobromide (**GH**, K_i_ = 2.30 ± 0.17) as standard.

Compound	K_i_ in µM/K_i_′ in µM (Type of Inhibition)	Compound	K_i_ in µM/K_i_′ in µM (Type of Inhibition)
**15**	11.94 ± 1.17/17.14 ± 1.67 (mixed-type)	**35**	0.07 ± 0.01/2.38 ± 0.48 (mixed-type)
**20**	8.37 ± 0.67/> 110 (mixed-type)	**40**	0.45 ± 0.01/> 10 (mixed-type)
**25**	1.60 ± 0.13/1.60 ±0.11 (mixed-type)	**45**	0.71 ± 0.04/4.88 ± 0.21 (mixed-type)
**30**	1.12 ± 0.01/4.46 ± 0.01 (mixed-type)	**50**	0.97 ± 0.04/3.09 ± 0.03 (mixed-type)

**Table 3:** Results of the docking studies with gold fitness values (PLP: Piecewise Linear Potential) and interaction energies (IE).

Compound	Gold Fitness (ChemPLP)	IE (kcal/mol)	K_i_
**31**	90.0	−57.8	4.42
**34**	98.7	−60.4	0.39
**35**	99.0	−64.0	0.07

## Supplementary Materials

Supplementary data related to this article can be found online.
