# Supplementary material for: Triterpene-Based Carboxamides Act as Good Inhibitors of Butyrylcholinesterase
Source: Molecules. 2019 Mar 7;24(5):948. doi: 10.3390/molecules24050948 (PMC6429507; doi:10.3390/molecules24050948)
Supplement: Supplementary file 1 [file molecules-24-00948-s001.pdf]

Supplementary Material

Triterpene-based carboxamides act as good inhibitors of butyrylcholinesterase

Anne Loesche[a], Michael Kahnt[a], Immo Serbian[a], Wolfgang Brandt[b], René Csuk[a,*]

[a] *Martin-Luther-University Halle-Wittenberg, Organic Chemistry, Kurt-Mothes-Str. 2, D-06120 Halle (Saale), Germany*

[b] *Leibniz Insitute of Plant Biochemistry, Bioorganic Chemistry, Weinberg 3, 06120 Halle (Saale), Germany*

1 Experimental Procedures and Analytical Data

*(3β)-N-(2-Aminoethyl)-3-acetyloxy-urs-12-en-28-amide (**11**)*

Compound **11** was prepared from **6** according to general procedure B using ethylenediamine as amino compound. Column chromatography (SiO_2, $CHCl_3$/MeOH 9:1) gave **11** (yield: 80%); m.p. 202–205 °C (lit.: 140–142 °C)[1]; $[\alpha]_D$ = +39.4° (*c* 0.355, $CHCl_3$); R_f = 0.48 ($CHCl_3$/MeOH 9:1); IR (KBr): ν = 3413*br s*, 2948*s*, 1735*s*, 1633*s*, 1526*s*, 1456*s*, 1370*s*, 1247*s*, 1174*w*, 1147*w*, 1092*w*, 1028*s*, 1006*m*, 986*m*, 755*m* cm^{-1}; ^1H NMR (500 MHz, $CDCl_3$): δ = 6.88 (*t*, *J* = 5.3 Hz, 1H, NH), 5.34 (*t*, *J* = 3.3 Hz, 1H, 12-H), 4.49 (*dd*, *J* = 10.0, 5.9 Hz, 1H, 3-H), 3.62 – 3.54 (*m*, 1H, 31-H_a), 3.38 – 3.30 (*m*, 1H, 31-H_b), 3.13 – 3.01 (*m*, 2H, 32-H_a, 32-H_b), 2.09 – 2.04 (*m*, 1H, 18-H), 2.04 (*s*, 3H, Ac), 2.03 – 1.87 (*m*, 3H, 16-H_a, 11-H_a, 11-H_b), 1.82 – 1.22 (*m*, 15H, 22-H_a, 16-H_b, 1-H_a, 15-H_a, 2-H_a, 2-H_b, 9-H, 22-H_b, 6-H_a, 21-H_a, 7-H_a, 19-H, 6-H_b, 7-H_b, 21-H_b), 1.08 (*s*, 3H, 27-H), 1.07 – 0.95 (*m*, 3H, 1-H_b, 15-H_b, 20-H), 0.96 – 0.92 (*m*, 4H, 25-H, 20-H), 0.89 – 0.85 (*m*, 6H, 23-H, 29-H), 0.85 (*s*, 3H, 24-H), 0.84 – 0.80 (*m*, 1H, 5-H), 0.74 (*s*, 3H, 26-H) ppm; ^{13}C NMR (126 MHz, $CDCl_3$): δ = 180.2 (C-28), 171.1 (Ac), 139.3 (C-13), 126.0 (C-12), 81.0 (C-3), 55.4 (C-5), 53.1 (C-18), 47.9 (C-17), 47.6 (C-9), 42.4 (C-14), 40.6 (C-32), 39.8 (C-19), 39.7 (C-8), 39.0 (C-20), 38.7 (C-31), 38.5 (C-1), 37.8 (C-4), 37.4 (C-22), 37.0 (C-10), 32.8 (C-7), 31.0 (C-21), 28.2 (C-23), 28.0 (C-15), 24.8 (C-16), 23.7 (C-2), 23.5 (C-11), 23.5 (C-27), 21.4 (Ac), 21.3 (C-30), 18.3 (C-6), 17.4 (C-29), 17.2 (C-26), 16.9 (C-24), 15.7 (C-25) ppm; MS (ESI, MeOH): *m/z* = 541 (100%, $[M+H]^+$); analysis calcd for $C_{34}H_{56}N_2O_3$ (540.83): C 75.51, H 10.44, N 5.18; found: C 75.32, H 10.61, N 5.01.

*(3β)-N-(2-Aminoethyl)-3-acetyloxy-lup-20(29)-en-28-amide (**14**)*

Compound **14** was prepared from **9** according to general procedure B using ethylenediamine as amino compound. Column chromatography (SiO_2, $CHCl_3$/MeOH 9:1) gave **14** (yield: 83%); m.p. 150 – 154 °C; $[\alpha]_D$ = +8.4° (*c* 0.330, $CHCl_3$); R_f = 0.38 ($CHCl_3$/MeOH 9:1); IR (KBr): ν = 3442*br s*, 2946*s*, 1734*m*, 1638*m*, 1522*m*, 1452*m*, 1376*m*, 1248*s*, 1030*m* cm^{-1}; ^{1}H NMR (400 MHz, $CDCl_3$): δ = 6.39 (*t*, *J* = 5.5 Hz, 1H, N*H*), 4.73 – 4.70 (*m*, 1H, 29-H_a), 4.60 – 4.57 (*m*, 1H, 29-H_b), 4.45 (*dd*, *J* = 10.0, 6.2 Hz, 1H, 3-H), 3.40 – 3.33 (*m*, 2H, 31-H), 3.09 (*ddd*, *J* = 11.0, 11.0, 4.0 Hz, 1H, 19-H), 2.90 (*t*, *J* = 5.9 Hz, 2H, 32-H), 2.42 (*ddd*, *J* = 12.7, 12.7, 3.4 Hz, 1H, 13-H), 2.03 (*s*, 3H, Ac), 2.01 – 1.68 (*m*, 4H, 16-H_a, 21-H_a, 22-H_a,12-H_a), 1.67 (*s*, 3H, 30-H_a), 1.66 – 1.07 (*m*, 16H, 22-H_b, 2-H_a, 2-H_b, 18-H, 16-H_b, 15-H_a, 6-H_a, 1-H_a, 11-H_a, 6-H_b, 21-H_b, 7-H_a, 7-H_b, 9-H, 11-H_b, 15-H_b), 1.05 – 0.88 (*m*, 2H, 12-H_b, 1-H_b), 0.95 (*s*, 3H, 27-H), 0.92 (*s*, 3H, 26-H), 0.83 (*s*, 6H, 25-H, 23-H), 0.82 (*s*, 3H, 24-H), 0.80 – 0.74 (*m*, 1H, 5-H) ppm; ^{13}C NMR (101 MHz, $CDCl_3$): δ = 177.2 (C-28), 171.1 (Ac), 150.9 (C-20), 109.6 (C-29), 81.1 (C-3), 55.9 (C-17), 55.6 (C-5), 50.7 (C-9), 50.3 (C-18), 47.0 (C-19), 42.6 (C-14), 41.5 (C-32), 40.9 (C-8), 40.8 (C-31), 38.6 (C-1, C-22), 37.9 (C-4), 37.9 (C-13), 37.3 (C-10), 34.5 (C-7), 33.8 (C-16), 31.1 (C-21), 29.6 (C-15), 28.1 (C-23), 25.7 (C-12), 23.8 (C-2), 21.5 (Ac), 21.1 (C-11), 19.6 (C-30), 18.3 (C-6), 16.6 (C-24), 16.4 (C-25), 16.3 (C-26), 14.8 (C-27) ppm; MS (ESI, MeOH): *m/z* = 541 (100%, $[M+H]^+$); analysis calcd for $C_{34}H_{56}N_2O_3$ (540.83): C 75.51, H 10.44, N 5.18; found: C 75.35, H 10.67, N 5.02.

*(3β)-N-(2-Aminoethyl)-3-acetyloxy-20-oxo-30-norlupan-28-amide (**15**)*

Compound **15** was prepared from **10** according to general procedure B using ethylenediamine as amino compound. Column chromatography (SiO_2, $CHCl_3$/MeOH 9:1) gave **15** (yield: 86%); m.p. 230–234 °C; $[\alpha]_D$ = –8.5° (*c* 0.160, $CHCl_3$); R_f = 0.34 ($CHCl_3$/MeOH/NH_4OH 90:10:0.1); IR (KBr): ν = 3425*br s*, 2945*s*, 1734*m*, 1712*m*, 1639*m*, 1522*m*, 1452*m*, 1370*m*, 1249*s*, 1197*w*, 1029*m*, 979*m* cm^{-1}; ^{1}H NMR (500 MHz, $CDCl_3$): δ = 6.25 (*t*, *J* = 6.3, 1H, NH), 4.46 (*dd*, *J* = 10.9, 5.2 Hz, 1H, 3-H), 3.43 (*ddd*, *J* = 11.3, 11.3, 4.4 Hz, 1H, 19-H), 3.35–3.23 (*m*, 2H, 30-H), 2.87 (*dd*, *J* = 5.8, 5.8 Hz, 2H, 31-H), 2.25–2.18 (*m*, 1H, 13-H), 2.16 (*s*, 3H, 29-H), 2.14–2.04 (*m*, 2H, 18-H, 21-H_a), 2.03 (*s*, 3H, Ac), 1.96 (*ddd*, *J* = 13.6, 2.9, 2.9 Hz, 1H, 16-H_a), 1.79 (*dd*, *J* = 11.8, 7.7 Hz, 1H, 22-H_a), 1.68–1.55 (*m*, 4H, 1-H_a, 16-H_b, 2-H_a, 2-H_b), 1.55–1.36 (*m*, 5H, 22-H_b, 6-H_a, 15-H_a, 21-H_b, 11-H_a), 1.37–1.21 (*m*, 5H, 6-H_b, 7-H_a, 7-H_b, 9-H, 11-H_b), 1.18 (*ddd*, *J* = 13.2, 2.8, 2.8 Hz, 1H, 15-H_b), 1.12–1.01 (*m*, 2H, 12-H_a, 12-H_b), 0.99 (*s*, 3H, 27-H), 0.99–0.92 (*m*, 1H, 1-H_b), 0.91 (*s*, 3H, 26-H), 0.83 (*s*, 6H, 23-H, 25-

H), 0.82 (*s*, 3H, 24-H), 0.81–0.75 (*m*, 1H, 5-H) ppm; ^{13}C NMR (126 MHz, $CDCl_3$): δ = 213.0 (C-20), 176.7 (C-28), 171.1 (Ac), 81.0 (C-3), 55.7 (C-17), 55.6 (C-5), 51.3 (C-19), 50.6 (C-9), 50.2 (C-18), 42.4 (C-14), 41.6 (C-31), 41.4 (C-32), 40.8 (C-8), 38.5 (C-1), 38.2 (C-22), 38.0 (C-4), 37.3 (C-10), 37.0 (C-13), 34.4 (C-7), 33.1 (C-16), 30.4 (C-29), 29.7 (C-15), 28.8 (C-21), 28.1 (C-23), 27.4 (C-12), 23.8 (C-2), 21.5 (Ac), 21.1 (C-11), 18.3 (C-6), 16.6 (C-24), 16.3 (C-25), 16.3 (C-26), 14.8 (C-27) ppm; MS (ESI, MeOH): *m/z* = 543 (100%, $[M+H]^+$), 1085 (10%, $[2M+H]^+$); analysis calcd for $C_{33}H_{54}N_2O_4$ (542.81): C 73.02, H 10.03, N 5.16; found: C 72.84, H 10.19, N 5.04.

*(3β)-N-(2-Aminoethyl)-3-hydroxy-urs-12-en-28-amide (**16**)*

Compound **16** was prepared from **11** according to general procedure C. Column chromatography (SiO_2, $CHCl_3$/MeOH/NH_4OH 90:10:0.1) gave **16** (yield: 85%); m.p. 139–142 °C (lit.: 145–147 °C)[1]; $[\alpha]_D$ = +38.6° (*c* 0.300, $CHCl_3$); R_f = 0.34 ($CHCl_3$/MeOH 9:1); IR (KBr): ν = 3425*br s*, 2926*s*, 1638*m*, 1529*m*, 1454*m*, 1386*w*, 1092*w*, 1046*m*, 755*m* cm^{-1}; ^{1}H NMR (400 MHz, $CDCl_3$): δ = 6.36 (*t*, *J* = 5.4 Hz, 1H, N*H*), 5.33 (*t*, *J* = 3.4 Hz, 1H, 12-H), 3.46 – 3.36 (*m*, 1H, 31-H_a), 3.21 (*dd*, *J* = 11.1, 4.7 Hz, 1H, 3-H), 3.13 – 3.02 (*m*, 1H, 31-H_b), 2.82 (*t*, *J* = 5.9 Hz, 2H, 32-H_a, 32-H_b), 2.05 – 1.82 (*m*, 5H, 16-H_a, 11-H_a, 11-H_b, 18-H, 22-H_a), 1.77 – 1.23 (*m*, 14H, 16-H_b, 15-H_a, 1-H_a, 2-H_a, 2-H_b, 9-H, 6-H_a, 21-H_a, 7-H_a, 22-H_b, 19-H, 6-H_b, 21-H_b, 7-H_b), 1.09 (*s*, 3H, 27-H), 1.07 – 0.99 (*m*, 2H, 15-H_b, 1-H_b), 0.98 (*s*, 3H, 23-H), 0.96 – 0.93 (*m*, 4H, 20-H, 30-H), 0.91 (*s*, 3H, 25-H), 0.87 (*d*, *J* = 6.5 Hz, 3H, 29-H), 0.78 (*s*, 6H, 24-H, 26-H), 0.74 – 0.69 (*m*, 1H, 5-H) ppm; ^{13}C NMR (101 MHz, $CDCl_3$): δ = 178.8 (C-28), 139.7 (C-13), 125.9 (C-12), 79.1 (C-3), 55.3 (C-5), 53.9 (C-18), 48.0 (C-17), 47.7 (C-9), 42.6 (C-14), 41.8 (C-31), 41.3 (C-32), 39.9 (C-19), 39.7 (C-8), 39.2 (C-20), 38.9 (C-4), 38.8 (C-1), 37.5 (C-22), 37.1 (C-10), 32.9 (C-7), 31.1 (C-21), 28.3 (C-23), 28.0 (C-15), 27.4 (C-2), 25.0 (C-16), 23.6 (C-11), 23.4 (C-27), 21.4 (C-30), 18.4 (C-6), 17.4 (C-29), 17.1 (C-26), 15.8 (C-24), 15.7 (C-25) ppm; MS (ESI, MeOH): *m/z* = 499 (100%, $[M+H]^+$); analysis calcd for $C_{32}H_{54}N_2O_2$ (498.80): C 77.06, H 10.91, N 5.62; found: C 76.92, H 11.08, N 5.40.

*(3β)-N-(2-Aminoethyl)-3-hydroxy-lup-20(29)-en-28-amide (**19**)*

Compound **19** was prepared from **14** according to general procedure C. Column chromatography (SiO_2, $CHCl_3$/MeOH 9:1) gave **19** (yield: 86%); m.p. 215–220 °C; $[\alpha]_D$ = +4.5° (*c* 0.300, DMSO); R_f = 0.28 ($CHCl_3$/MeOH 9:1); IR (KBr): ν = 3424*br s*, 2941*m*, 1636*m*, 1449*m*, 1044*m*, 879*w* cm^{-1}; ^{1}H NMR (400 MHz, DMSO-*d*$_6$): δ = 7.53 (*t*, *J* = 5.5 Hz, 1H, NH), 4.67 – 4.63 (*m*, 1H, 29-H_a), 4.54 – 4.51 (*m*, 1H, 29-H_b), 3.15 – 2.92 (*m*, 4H, 32-H_a,

19-H, 32-H_b, 3-H), 2.60 – 2.51 (*m*, 3H, 13-H, 31-H_a, 31-H_b), 2.16 – 2.09 (*m*, 1H, 16-H_a), 1.82 – 1.65 (*m*, 2H, 22-H_a, 21-H_a), 1.62 (*s*, 3H, 30-H), 1.61 – 0.92 (*m*, 17H, 12-H_a, 1-H_a, 2-H_a, 2-H_b, 6-H_a, 18-H, 16-H_b, 11-H_a, 22-H_b, 15-H_a, 6-H_b, 7-H_a, 7-H_b, 21-H_b, 9-H, 11-H_b, 15-H_b), 0.92 – 0.78 (*m*, 2H, 12-H_b, 1-H_b), 0.91 (*s*, 3H, 27-H), 0.87 (*s*, 3H, 23-H), 0.84 (*s*, 3H, 26-H), 0.76 (*s*, 3H, 25-H), 0.65 (*s*, 3H, 24-H), 0.64 – 0.60 (*m*, 1H, 5-H) ppm; ^{13}C NMR (101 MHz, DMSO-d_6): δ = 175.6 (C-28), 150.9 (C-20), 109.1 (C-29), 76.8 (C-3), 54.9 (C-5), 54.9 (C-17), 50.1 (C-9), 49.7 (C-18), 46.2 (C-19), 41.9 (C-14), 41.8 (C-32), 41.4 (C-31), 40.3 (C-8), 38.5 (C-4), 38.3 (C-1), 37.7 (C-22), 36.7 (C-10), 36.6 (C-13), 34.0 (C-7), 32.4 (C-16), 30.3 (C-21), 28.9 (C-15), 28.1 (C-23), 27.1 (C-2), 25.2 (C-12), 20.6 (C-11), 19.0 (C-30), 17.9 (C-6), 15.9 (C-25), 15.8 (C-26), 15.7 (C-24), 14.3 (C-27) ppm; MS (ESI, MeOH): *m/z* = 499 (100%, $[M+H]^+$); analysis calcd for $C_{32}H_{54}N_2O_2$ (498.80): C 77.06, H 10.91, N 5.62; found: C 76.81, H 11.07, N 5.55.

*(3β)-N-(2-Aminoethyl)-3-hydroxy-20-oxo-30-norlupan-28-amide (**20**)*

Compound **20** was prepared from **15** according to general procedure C. Column chromatography (SiO_2, $CHCl_3$/MeOH/NH_4OH 90:10:0.1) gave **20** (yield: 86%); m.p. 218–221 °C; $[\alpha]_D$ = –29.8° (*c* 0.325, MeOH); R_f = 0.22 ($CHCl_3$/MeOH 9:1); IR (KBr): ν = 3441*br s*, 2942*m*, 1636*m*, 1448*m*, 1034*w* cm^{-1}; ^{1}H NMR (500 MHz, CD_3OD): δ = 3.39 (*ddd*, *J* = 11.4, 11.4, 4.4 Hz, 1H, 19-H), 3.35–3.30 (*m*, 1H, 30-H_a), 3.26–3.14 (*m*, 1H, 30-H_b), 2.76 (*ddd*, *J* = 6.5, 6.5, 2.6 Hz, 1H, 31-H), 2.42 (*ddd*, *J* = 12.7, 12.7, 3.7 Hz, 1H, 13-H), 2.21 (*s*, 3H, 29-H), 2.21–2.17 (*m*, 1H, 16-H_a), 2.12–1.99 (*m*, 2H, 18-H, 21-H_a), 1.94–1.85 (*m*, 1H, 22-H_a), 1.73 (*ddd*, *J* = 6.6, 3.0, 3.0 Hz, 1H, 1-H_a), 1.70–1.48 (*m*, 8H, 2-H_a, 2-H_b, 16-H_b, 6-H_a, 22-H_b, 11-H_a, 15-H_a, 21-H_b), 1.47–1.30 (*m*, 5H, 6-H_b, 7-H_a, 7-H_b, 9-H, 11-H_b), 1.24 (*ddd*, *J* = 12.8, 2.6, 2.6 Hz, 1H, 15-H_b), 1.20–1.04 (*m*, 2H, 12-H_a, 12-H_b), 1.05 (*s*, 3H, 27-H), 0.99–0.93 (*m*, 1H, 1-H_b), 0.99 (*s*, 6H, 23-H, 26-H), 0.90 (*s*, 3H, 25-H), 0.79 (*s*, 3H, 24-H), 0.78–0.73 (*m*, 1H, 5-H) ppm; ^{13}C NMR (126 MHz, CD_3OD): δ = 215.4 (C-20), 178.9 (C-28), 79.3 (C-3), 56.7 (C-17), 56.6 (C-5), 52.3 (C-19), 51.8 (C-9), 51.2 (C-18), 43.0 (C-14), 42.4 (C-31), 41.9 (C-30), 41.7 (C-8), 39.8(C-4), 39.7 (C-1), 38.8 (C-22), 38.1 (C-10), 37.8 (C-13), 35.3 (C-7), 33.1 (C-16), 30.4 (C-15), 29.6 (C-29), 29.3 (C-21), 28.3 (C-23), 28.2 (C-12), 27.8 (C-2), 21.9 (C-11), 19.2 (C-6), 16.5 (C-26), 16.4 (C-25), 15.8 (C-24), 14.8 (C-27) ppm; MS (ESI, MeOH): *m/z* = 501 (100%, $[M+H]^+$); analysis calcd for $C_{31}H_{52}N_2O_3$ (500.77): C 74.35, H 10.47, N 5.59; found: C 74.17, H 5.77, N 5.31.

(3β)-N-[2-(Dimethylamino)ethyl]-3-acetyloxy-urs-12-en-28-amide (**21**)

Compound **21** was prepared from **6** according to general procedure B using *N*,*N*-dimethylethylenediamine as amino compound. Column chromatography (SiO_2, $CHCl_3$/MeOH 95:5) gave **21** (yield: 88%); m.p. 121–124 °C; $[\alpha]_D$ = +44.9° (*c* 0.300, $CHCl_3$); R_f = 0.49 ($CHCl_3$/MeOH 9:1); IR (KBr): ν = 3422*br m*, 2937*m*, 1734*m*, 1636*m*, 1522*w*, 1457*m*, 1384*s*, 1247*m*, 1028*m* cm^{-1}; ^1H NMR (500 MHz, $CDCl_3$): δ = 6.68 (*t*, *J* = 5.0 Hz, 1H, NH), 5.33 (*t*, *J* = 3.4 Hz, 1H, 12-H), 4.48 (*dd*, *J* = 10.5, 5.6 Hz, 1H, 3-H), 3.62 – 3.52 (*m*, 1H, 31-H_a), 3.29 – 3.21 (*m*, 1H, 31-H_b), 2.83 (*t*, *J* = 5.3 Hz, 2H, 32-H), 2.58 (*s*, 6H, 33-H, 33'-H), 2.03 (*s*, 3H, Ac), 2.02 – 1.86 (*m*, 4H, 16-H_a, 18-H, 11-H_a, 11-H_b), 1.83 – 1.76 (*m*, 1H, 22-H_a), 1.73 – 1.67 (*m*, 1H, 16-H_b), 1.67 – 1.57 (*m*, 4H, 1-H_a, 15-H_a, 2-H_a, 2-H_b), 1.57 – 1.26 (*m*, 9H, 9-H, 6-H_a, 7-H_a, 21-H_a, 22-H_b, 19-H, 6-H_b, 21-H_b, 7-H_b), 1.07 (*s*, 3H, 27-H), 1.06 – 0.94 (*m*, 3H, 1-H_b, 15-H_b, 20-H), 0.93 (*d*, *J* = 6.1 Hz, 3H, 30-H), 0.93 (*s*, 3H, 25-H), 0.87 (*d*, *J* = 6.5 Hz, 3H, 29-H), 0.85 (*s*, 3H, 23-H), 0.84 (*s*, 3H, 24-H), 0.83 – 0.80 (*m*, 1H, 5-H), 0.75 (*s*, 3H, 26-H) ppm; ^{13}C NMR (126 MHz, $CDCl_3$): δ = 179.1 (C-28), 171.1 (Ac), 139.2 (C-13), 126.0 (C-12), 81.0 (C-3), 57.7 (C-32), 55.4 (C-5), 53.4 (C-18), 47.9 (C-17), 47.6 (C-9), 44.6 (C-33, C-33'), 42.4 (C-14), 39.8 (C-19), 39.7 (C-8), 39.0 (C-20), 38.4 (C-1), 37.8 (C-4), 37.4 (C-22), 37.0 (C-10), 35.9 (C-31), 32.8 (C-7), 31.0 (C-21), 28.2 (C-23), 28.0 (C-15), 24.8 (C-16), 23.7 (C-2), 23.5 (C-11), 23.4 (C-27), 21.4 (Ac), 21.3 (C-30), 18.3 (C-6), 17.3 (C-29), 17.1 (C-26), 16.9 (C-24), 15.7 (C-25) ppm; MS (ESI, MeOH): *m/z* = 569 (100%, $[M+H]^+$); analysis calcd for $C_{36}H_{60}N_2O_3$ (568.89): C 76.01, H 10.63, N 4.92; found: C 75.87, H 10.84, N 4.69.

(3β)-N-[2-(Dimethylamino)ethyl]-3-acetyloxy-lup-20(29)-en-28-amide (**24**)

Compound **24** was prepared from **9** according to general procedure B using *N*,*N*-dimethylethylenediamine as amino compound. Column chromatography (SiO_2, $CHCl_3$/MeOH 95:5) gave **24** (yield: 94%); m.p. 108–110 °C; $[\alpha]_D$ = +16.4° (*c* 0.320, $CHCl_3$); R_f = 0.51 ($CHCl_3$/MeOH 9:1); IR (KBr): ν = 3420*br s*, 2945*s*, 2869*m*, 1736*s*, 1641*m*, 1456*s*, 1375*m*, 1246*s*, 1195*w*, 1029*m*, 979*m* cm^{-1}; ^1H NMR (500 MHz, $CDCl_3$): δ = 6.24 (*t*, *J* = 4.7 Hz, 1H, NH), 4.74 – 4.72 (*m*, 1H, 29-H_a), 4.60 – 4.58 (*m*, 1H, 29-H_b), 4.46 (*dd*, *J* = 10.4, 5.9 Hz, 1H, 3-H), 3.40 – 3.24 (*m*, 2H, 31-H), 3.11 (*ddd*, *J* = 11.0, 11.0, 4.2 Hz, 1H, 19-H), 2.47 – 2.38 (*m*, 3H, 32-H + 13-H), 2.26 (*s*, 6H, 33-H, 33'-H), 2.03 (*s*, 3H, Ac), 2.03 – 1.89 (*m*, 2H, 16-H_a, 21-H_a), 1.80 – 1.74 (m, 1H, 22-H_a), 1.73 – 1.63 (*m*, 2H, 12-H_a, 22-H_b), 1.68 (*s*, 3H, 30-H), 1.63 – 1.11 (*m*, 15H, 2-H_a, 2-H_b, 18-H, 16-H_b, 15-H_a, 6-H_a, 11-H_a, 1-H_a, 6-H_b, 7-H_a, 7-H_b, 21-H_b, 9-H, 11-H_b, 15-H_b), 1.05 – 0.94 (*m*, 2H, 12-H_b, 1-H_b), 0.96 (*s*, 3H, 27-H), 0.94 (*s*, 3H, 26-H),

0.83 (*s*, 6H, 23-H, 25-H), 0.82 (*s*, 3H, 24-H), 0.80 – 0.76 (*m*, 1H, 5-H) ppm; ^{13}C NMR (126 MHz, $CDCl_3$): δ = 176.5 (C-28), 171.1 (Ac), 151.2 (C-20), 109.5 (C-29), 81.1 (C-3), 58.3 (C-32), 55.9 (C-17), 55.6 (C-5), 50.7 (C-9), 50.2 (C-18), 47.1 (C-19), 45.3 (C-33, C-33'), 42.7 (C-14), 40.9 (C-8), 38.6 (C-22), 38.6 (C-1), 38.0 (C-13), 38.0 (C-4), 37.3 (C-10), 36.6 (C-31), 34.5 (C-7), 33.8 (C-16), 31.1 (C-21), 29.6 (C-15), 28.1 (C-23), 25.8 (C-12), 23.9 (C-2), 21.5 (Ac), 21.1 (C-11), 19.6 (C-30), 18.4 (C-6), 16.6 (C-24), 16.4 (C-25), 16.3 (C-26), 14.8 (C-27) ppm; MS (ESI, MeOH): *m/z* = 569 (100%, $[M+H]^+$); analysis calcd for $C_{26}H_{60}N_2O_3$ (568.89): C 76.01, H 10.63, N 4.92; found: C 75.77, H 10.84, N 4.63.

*(3β)-N-[2-(Dimethylamino)ethyl]-3-acetyloxy-20-oxo-30-norlupan-28-amide (**25**)*

Compound **25** was prepared from **10** according to general procedure B using *N,N*-dimethylethylenediamine as amino compound. Column chromatography (SiO_2, $CHCl_3$/MeOH 9:1) gave **25** (yield: 93%); m.p. 143–147 °C; $[\alpha]_D$ = –6.2° (*c* 0.395, $CHCl_3$); R_f = 0.46 (silica gel, chloroform/methanol 9:1); IR (KBr): ν = 3409*br m*, 2946*s*, 2871*m*, 1733*m*, 1654*m*, 1522*m*, 1450*m*, 1384*s*, 1248s, 1196*m*, 1162*w*, 1029*m*, 979*m* cm^{-1}; ^{1}H NMR (500 MHz, $CDCl_3$): δ = 6.98 (*br* s, 1H, NH), 4.45 (*dd*, *J* = 11.0, 5.0 Hz, 1H, 3-H), 3.56–3.48 (*m*, 2H, 30-H), 3.34 (*td*, *J* = 11.5, 3.9 Hz, 1H, 19-H), 3.02–2.94 (*m*, 2H, 31-H), 2.71 (*s*, 6H, 32-H, 32'-H), 2.21 (*td*, *J* = 12.1, 4.0 Hz, 1H, 13-H), 2.14 (*s*, 3H, 29-H), 2.08 – 2.03 (*m*, 2H, 18-H, 16-H_a), 2.02 (*s*, 3H, Ac), 2.00 – 1.91 (*m*, 1H, 21-H_a), 1.84 – 1.78 (*m*, 1H, 22-H_a), 1.67 – 1.13 (*m*, 15H, 1-H_a, 2-H_a, 2-H_b, 16-H_b, 22-H_b, 6-H_a, 21-H_b, 11-H_a, 7-H_a, 7-H_b, 15-h_a, 6-H_b, 9-H, 11-H_b, 15-H_b), 1.10 – 0.91 (*m*, 3H, 12-H_a, 12-H_b, 1-H_b), 0.97 (*s*, 3H, 27-H), 0.89 (*s*, 3H, 26-H), 0.82 (*s*, 6H, 23-H, 25-H), 0.81 (*s*, 3H, 24-H), 0.80 – 0.74 (*m*, 1H, 5-H) ppm; ^{13}C NMR (126 MHz, $CDCl_3$): δ = 212.8 (C-20), 177.7 (C-28), 171.0 (Ac), 81.0 (C-3), 58.2 (C-31), 55.8 (C-17), 55.5 (C-5), 51.3 (C-19), 50.5 (C-9), 50.2 (C-18), 44.6 (C-32, C-32'), 42.4 (C-14), 40.8 (C-8), 38.5 (C-1), 37.9 (C-22), 37.9 (C-4), 37.3 (C-10), 37.0 (C-13), 35.7 (C-30), 34.4 (C-7), 32.6 (C-16), 30.2 (C-29), 29.6 (C-15), 28.6 (C-21), 28.1 (C-23), 27.3 (C-12), 23.8 (C-2), 21.4 (Ac), 21.1 (C-11), 18.3 (C-6), 16.6 (C-24), 16.3 (C-25), 16.2 (C-26), 14.8 (C-27) ppm; MS (ESI, MeOH): *m/z* = 571 (100%, $[M+H]^+$); analysis calcd for $C_{35}H_{58}N_2O_4$ (570.44): C 73.64, H 10.24, N 4.91; found: C 73.51, H 10.39, N 4.80.

*(3β)-N-[2-(Dimethylamino)ethyl]-3-hydroxy-urs-12-en-28-amide (**26**)*

Compound **26** was prepared from **21** according to general procedure C. Column chromatography (SiO_2, $CHCl_3$/MeOH 95:5) gave **26** (yield: 86%); m.p. 270–273 °C (decomp.); $[\alpha]_D$ = +38.5° (*c* 0.375, $CHCl_3$); R_f = 0.44 ($CHCl_3$/MeOH 9:1); IR (KBr): ν =

3402*br s*, 2924*s*, 2868*s*, 2684*m*, 1658*s*, 1518*m*, 1460*s*, 1386*m*, 1212*w*, 1138*w*, 1046*m*, 1028*m*, 994*m* cm^{-1}; ^{1}H NMR (500 MHz, $CDCl_3$): δ = 7.18 (*dd*, *J* = 5.6, 5.6 Hz, 1H, NH), 5.41 (*t*, *J* = 3.6 Hz, 1H, 12-H), 3.78 – 3.68 (*m*, 1H, 31-H_a), 3.59 – 3.49 (*m*, 1H, 31-H_b), 3.21 (*dd*, *J* = 11.0, 4.7 Hz, 1H, 3-H), 3.18 – 3.13 (*m*, 2H, 32-H), 2.84 (*s*, 6H, 33-H, 33'-H), 2.20 (*d*, *J* = 10.5 Hz, 1H, 18-H), 2.03 (*ddd*, *J* = 13.7, 13.7, 4.2 Hz, 1H, 16-H_a), 1.96 – 1.91 (*m*, 2H, 11-H_a, 11-H_b), 1.81 – 1.26 (*m*, 15H, 22-H_a, 16-H_b, 15-H_a, 1-H_a, 2-H_a, 2-H_b, 6-H_a, 9-H, 22-H_b, 7-H_a, 21-H_a, 19-H, 6-H_b, 7-H_b, 21-H_b), 1.08 (*s*, 3H, 27-H), 1.07 – 0.96 (*m*, 3H, 15-H_b, 20-H, 1-H_b), 0.98 (*s*, 3H, 23-H), 0.93 (*d*, *J* = 6.5 Hz, 3H, 30-H), 0.90 (*s*, 3H, 25-H), 0.88 (*d*, *J* = 6.5 Hz, 3H, 29-H), 0.77 (*s*, 3H, 24-H), 0.73 (*s*, 3H, 26-H), 0.72 – 0.69 (*m*, 1H, 5-H) ppm; ^{13}C NMR (126 MHz, $CDCl_3$): δ = 179.6 (C-28), 138.8 (C-13), 126.2 (C-12), 79.1 (C-3), 58.2 (C-32), 55.3 (C-5), 52.7 (C-18), 48.0 (C-17), 47.7 (C-9), 44.2 (C-33), 43.9 (C-33'), 42.3 (C-14), 39.7 (C-8), 39.7 (C-19), 38.9 (C-1), 38.8 (C-20), 38.7 (C-4), 37.4 (C-22), 37.1 (C-10), 35.1 (C-31), 32.9 (C-7), 31.0 (C-21), 28.3 (C-23), 28.0 (C-15), 27.4 (C-2), 24.6 (C-16), 23.6 (C-27), 23.5 (C-11), 21.3 (C-30), 18.4 (C-6), 17.2 (C-29), 17.2 (C-26), 15.8 (C-24), 15.6 (C-25) ppm; MS (ESI, MeOH): *m/z* = 527 (100%, $[M+H]^+$); analysis calcd for $C_{34}H_{58}N_2O_2$ (526.85): C 77.51, H 11.10, N 5.32; found: C 77.37, H 11.25, N 5.17.

*(3β)-N-[2-(Dimethylamino)ethyl]-3-hydroxy-lup-20(29)-en-28-amide (**29**)*

Compound **29** was prepared from **24** according to general procedure C. Column chromatography (SiO_2, $CHCl_3$/MeOH 95:5) gave **29** (yield: 89%); m.p. 116–120 °C; $[\alpha]_D$ = –4.4° (*c* 0.330, MeOH); R_f = 0.43 ($CHCl_3$/MeOH 9:1); IR (KBr): ν = 3408*br s*, 2944*s*, 2866*s*, 1638*s*, 1528*m*, 1464*s*, 1376*m*, 1246*m*, 1194*m*, 1044*m*, 880*m* cm^{-1}; ^{1}H NMR (500 MHz, $CDCl_3$): δ = 6.26 (*t*, *J* = 4.9 Hz, 1H, NH), 4.73 – 4.71 (*m*, 1H, 29-H_a), 4.58 – 4.56 (*m*, 1H, 29-H_b), 3.37 – 3.22 (*m*, 2H, 31-H), 3.16 (*dd*, *J* = 11.0, 5.2 Hz, 1H, 3-H), 3.10 (*ddd*, *J* = 11.1, 11.1, 4.2 Hz, 1H, 19-H), 2.46 – 2.37 (*m*, 3H, 13-H, 32-H), 2.22 (*s*, 6H, 33-H, 33'-H), 2.06 – 1.89 (*m*, 2H, 16-H_a, 21-H_a), 1.79 – 1.72 (*m*, 1H, 22-H_a), 1.67 (*s*, 3H, 30-H), 1.72– 1.16 (*m*, 16H, 12-H_a, 1-H_a, 2-H_a, 2-H_b, 18-H, 6-H_a, 16-H_b, 15-H_a, 11-H_a, 22-H_b, 6-H_b, 7-H_a, 7-H_b, 21-H_b, 9-H, 11-H_b), 1.15 – 1.10 (*m*, 1H, 15-H_b), 1.04 – 0.96 (*m*, 1H, 12-H_b), 0.95 (*s*, 3H, 27-H), 0.95 (*s*, 3H, 23-H), 0.93 (*s*, 3H, 26-H), 0.91 – 0.81 (*m*, 1H, 1-H_b), 0.80 (*s*, 3H, 25-H), 0.74 (*s*, 3H, 24-H), 0.69 – 0.64 (*m*, 1H, 5-H) ppm; ^{13}C NMR (126 MHz, $CDCl_3$): δ = 176.4 (C-28), 151.2 (C-20), 109.4 (C-29), 79.1 (C-3), 58.3 (C-32), 55.9 (C-17), 55.5 (C-5), 50.8 (C-9), 50.2 (C-18), 47.0 (C-19), 45.3 (C-33, C-33'), 42.6 (C-14), 40.9 (C-8), 39.0 (C-4), 38.9 (C-1), 38.6 (C-22), 38.0 (C-13), 37.4 (C-10), 36.7 (C-31), 34.6 (C-7), 33.8 (C-16), 31.1 (C-21), 29.6 (C-15), 28.1 (C-23), 27.6 (C-2), 25.8 (C-12), 21.1 (C-11), 19.6 (C-30), 18.5 (C-6), 16.3 (C-26), 16.2 (C-

25), 15.5 (C-24), 14.8 (C-27) ppm; MS (ESI, MeOH): *m/z* = 527 (100%, $[M+H]^+$); analysis calcd for $C_{34}H_{58}N_2O_2$ (526.85): C 77.51, H 11.10, N 5.32; found: C 77.40, H 11.22, N 5.18.

*(3β)-N-[2-(Dimethylamino)ethyl]-3-hydroxy-20-oxo-30-norlupan-28-amide (**30**)*

Compound **30** was prepared from **25** according to general procedure C. Column chromatography (SiO_2, $CHCl_3$/MeOH 9:1) gave **30** (yield: 89%); m.p. 173–176 °C; $[\alpha]_D$ = –23.8° (*c* 0.630, MeOH); R_f = 0.39 (silica gel, $CHCl_3$/MeOH 9:1); IR (KBr): ν = 3424*br s*, 2942*m*, 2868*m*, 1694*m*, 1642*m*, 1526*m*, 1384*s*, 1198*w*, 1170*w*, 1044*w* cm^{-1}; ^1H NMR (500 MHz, $CDCl_3$): δ = 7.20 (*t*, *J* = 5.4 Hz, 1H, NH), 3.66 – 3.60 (*m*, 2H, 30-H), 3.34 (*ddd*, *J* = 11.2, 11.2, 4.1 Hz, 1H, 19-H), 3.21 – 3.14 (*m*, 3H, 3-H, 31-H), 2.85 (*s*, 6H, 32-H, 32’-H), 2.20 (*td*, *J* = 11.4, 4.1 Hz, 1H, 13-H), 2.15 (*s*, 3H, 29-H), 2.10 – 2.02 (*m*, 2H, 18-H, 16-H_a), 2.01 – 1.90 (*m*, 1H, 21-H_a), 1.87 – 1.77 (*m*, 1H, 22-H_a), 1.68 – 0.99 (*m*, 17H, 1-H_a, 2-H_a, 2-H_b, 16-H_b, 22-H_b, 6-H_a, 21-H_b, 11-H_a, 6-H_b, 7-H_a, 7-H_b, 15-H_a, 9-H, 11-H_b, 15-H_b, 12-H_a, 12-H_b), 0.98 (*s*, 3H, 27-H), 0.95 (*s*, 3H, 23-H), 0.89 (*s*, 3H, 26-H), 0.93 – 0.82 (*m*, 1H, 1-H_b), 0.80 (*s*, 3H, 25-H), 0.74 (*s*, 3H, 24-H), 0.70 – 0.65 (*m*, 1H, 5-H) ppm; ^{13}C NMR (126 MHz, $CDCl_3$): δ = 212.7 (C-20), 177.8 (C-28), 79.0 (C-3), 58.3 (C-31), 55.9 (C-17), 55.4 (C-5), 51.3 (C-19), 50.6 (C-9), 50.2 (C-18), 44.3 (C-32, C-32’), 42.4 (C-14), 40.8 (C-8), 39.0 (C-4), 38.8 (C-1), 37.8 (C-22), 37.4 (C-10), 37.0 (C-13), 35.1 (C-30), 34.4 (C-7), 32.5 (C-16), 30.2 (C-29), 29.7 (C-15), 28.7 (C-21), 28.1 (C-23), 27.5 (C-12), 27.4 (C-2), 21.1 (C-11), 18.4 (C-6), 16.3 (C-25), 16.3 (C-26), 15.5 (C-24), 14.9 (C-27) ppm; MS (ESI, MeOH): *m/z* = ; analysis calcd for $C_{33}H_{56}N_2O_3$ (528.43): C 74.95, H 10.67, N 5.30; found: C 74.78, H 10.92, N 5.17.

*(3β)-N-(2-Pyrrolidin-1-ylethyl)-3-acetyloxy-urs-12-en-28-amide (**31**)*

Compound **31** was prepared from **6** according to general procedure B using 1-(2-aminoethyl)pyrrolidine as amino compound. Column chromatography (SiO_2, $CHCl_3$/MeOH 95:5) gave **31** (yield: 92%); m.p. 156–159 °C; $[\alpha]_D$ = +45.9° (*c* 0.355, $CHCl_3$); R_f = 0.44 ($CHCl_3$/MeOH 9:1); IR (KBr): ν = 3404*br s*, 2948*s*, 1734*s*, 1651*s*, 1526*s*, 1456*s*, 1371*s*, 1246*s*, 1146*w*, 1091*m*, 1027*s*, 1006*m*, 985*m*, 753*m* cm^{-1}; ^1H NMR (500 MHz, $CDCl_3$): δ = 6.90 (*dd*, *J* = 11.5, 5.6 Hz, 1H, NH), 5.36 (*dd*, *J* = 7.1, 3.6 Hz, 1H, 12-H), 4.47 (*dd*, *J* = 10.5, 5.3 Hz, 1H, 3-H), 3.89 – 3.82 (*m*, 1H, 31-H_a), 3.82 – 3.73 (*m*, 2H, 33-H_a, 33’-H_a) 3.41 – 3.32 (*m*, 1H, 31-H_b), 3.30 – 3.21 (*m*, 2H, 32-H), 2.93 – 2.83 (*m*, 2H, 33-H_b, 33’-H_b), 2.23 – 2.06 (*m*, 4H, 34-H, 34’-H), 2.02 (*s*, 3H, Ac), 2.01 – 1.95 (*m*, 2H, 16-H_a, 18-H), 1.95 – 1.90 (*m*, 2H, 11-H_a, 11-H_b), 1.76 – 1.56 (*m*, 6H, 22-H_a, 16-H_b, 1-H_a, 15-H_a, 2-H_a, 2-H_b), 1.55 – 1.41 (*m*, 5H, 9-H, 6-H_a, 22-H_b, 7-H_a, 21-H_a), 1.41 – 1.21 (*m*, 4H, 19-H, 6-H_b, 7-H_b, 21-H_b), 1.06 (*s*,

3H, 27-H), 1.10 – 0.94 (*m*, 3H, 1-H_b, 15-H_b, 20-H), 0.92 (*s*, 3H, 25-H), 0.91 (*d*, *J* = 6.1 Hz, 3H, 30-H), 0.86 (*d*, *J* = 6.5 Hz, 3H, 29-H), 0.84 (*s*, 3H, 23-H), 0.83 (*s*, 3H, 24-H), 0.82 – 0.78 (*m*, 1H, 5-H), 0.71 (*s*, 3H, 26-H) ppm; ^{13}C NMR (126 MHz, $CDCl_3$): δ = 179.8 (C-28), 171.1 (Ac), 138.9 (C-13), 126.0 (C-12), 80.9 (C-3), 55.3 (C-5), 55.0 (C-32), 54.8 (C-33, C-33'), 52.9 (C-18), 47.9 (C-17), 47.5 (C-9), 42.3 (C-14), 39.7 (C-19), 39.7 (C-8), 38.8 (C-20), 38.4 (C-1), 37.8 (C-4), 37.4 (C-22), 37.0 (C-10), 36.2 (C-31), 32.8 (C-7), 30.9 (C-21), 28.2 (C-23), 27.9 (C-15), 24.7 (C-16), 23.6 (C-2), 23.5 (C-27), 23.4 (C-11), 23.3 (C-34, C-34'), 21.4 (Ac), 21.3 (C-30), 18.3 (C-6), 17.2 (C-29), 17.1 (C-26), 16.8 (C-24), 15.6 (C-25) ppm; MS (ESI, MeOH): *m/z* = 595 (100%, $[M+H]^+$); analysis calcd for $C_{38}H_{62}N_2O_3$ (594.93): C 76.72, H 10.50, N 4.71; found: C 76.60, H 10.72, N 4.59.

*(3β)-N-(2-Pyrrolidin-1-ylethyl)-3-acetyloxy-lup-20(29)-en-28-amide (**34**)*

Compound **34** was prepared from **9** according to general procedure B using 1-(2-aminoethyl)pyrrolidine as amino compound. Column chromatography (SiO_2, $CHCl_3$/MeOH 95:5) gave **34** (yield: 86%); m.p. 141–145 °C; $[\alpha]_D$ = +11.7° (*c* 0.330, $CHCl_3$); R_f = 0.53 ($CHCl_3$/MeOH 9:1); IR (KBr): ν =3422*br m*, 2946*s*, 1734*m*, 1640*m*, 1451*m*, 1384*s*, 1247*s*, 1029*m*, 979*m* cm^{-1}; ^{1}H NMR (400 MHz, $CDCl_3$): δ = 7.23 (*t*, *J* = 5.5 Hz, 1H, NH), 4.72 – 4.70 (*m*, 1H, 29-H_a), 4.58 – 4.56 (*m*, 1H, 29-H_b), 4.44 (*dd*, *J* = 10.8, 5.5 Hz, 1H, 3-H), 3.68 – 3.56 (*m*, 2H, 31-H), 3.47 – 3.27 (*m*, 4H, 33-H, 33'-H), 3.24 (*t*, *J* = 6.1 Hz, 2H, 32-H), 3.05 (*ddd*, *J* = 10.9, 10.9, 4.2 Hz, 1H, 19-H), 2.39 (*ddd*, *J* = 12.8, 12.8, 3.5 Hz, 1H, 13-H), 2.13 – 2.04 (*m*, 5H, 34-H, 34'-H, 16-H_a), 2.02 (*s*, 3H, Ac), 1.89 – 1.76 (*m*, 2H, 21-H_a, 22-H_a), 1.66 (*s*, 3H, 30-H), 1.71 – 1.11 (*m*, 17H, 12-H_a, 1-H_a, 2-H_a, 2-H_b, 18-H, 16-H_b, 6-H_a, 22-H_b, 11-H_a, 21-H_b, 7-H_a, 7-H_b, 15-H_a, 6-H_b, 9-H, 11-H_b, 15-H_b), 1.03 – 0.91 (*m*, 2H, 12-H_b, 1-H_b), 0.93 (*s*, 3H, 27-H), 0.89 (*s*, 3H, 26-H), 0.82 (*s*, 6H, 23-H, 25-H), 0.81 (*s*, 3H, 24-H), 0.78 – 0.75 (*m*, 1H, 5-H) ppm; ^{13}C NMR (101 MHz, $CDCl_3$): δ = 177.8 (C-28), 171.1 (Ac), 151.0 (C-20), 109.6 (C-29), 81.1 (C-3), 55.9 (C-17), 55.6 (C-5), 55.5 (C-32), 54.8 (C-33, C-33'), 50.6 (C-9), 50.3 (C-18), 47.0 (C-19), 42.6 (C-14), 40.9 (C-8), 38.5 (C-1), 38.2 (C-22), 37.9 (C-13), 37.9 (C-4), 37.3 (C-10), 36.3 (C-31), 34.5 (C-7), 33.2 (C-16), 31.0 (C-21), 29.6 (C-15), 28.1 (C-23), 25.7 (C-12), 23.8 (C-2), 23.4 (C-34, C-34'), 21.4 (Ac), 21.1 (C-11), 19.5 (C-30), 18.3 (C-6), 16.6 (C-24), 16.3 (C-25), 16.3 (C-26), 14.7 (C-27) ppm; MS (ESI, MeOH): *m/z* = 595 (100%, $[M+H]^+$); analysis calcd for $C_{38}H_{62}N_2O_3$ (594.93): C 76.72, H 10.50, N 4.71; found: C 76.50, H 10.74, N 4.51.

*(3β)-N-(2-Pyrrolidin-1-ylethyl)-3-acetyloxy-20-oxo-30-norlupan-28-amide (**35**)*

Compound **35** was prepared from **10** according to general procedure B using 1-(2-aminoethyl)pyrrolidine as amino compound. Column chromatography (SiO_2, $CHCl_3$/MeOH 9:1) gave **35** (yield: 89%); m.p. 162–165 °C; $[\alpha]_D$ = –7.8° (*c* 0.330, $CHCl_3$); R_f = 0.35 (silica gel, chloroform/methanol 9:1); IR (KBr): ν = 3404*br s*, 2947*s*, 1733*s*, 1652*s*, 1525*s*, 1383*s*, 1248*s*, 1196*m*, 1029*m*, 979*m*, 752*m* cm^{-1}; ^{1}H NMR (500 MHz, $CDCl_3$): *δ* = 7.39 (*t*, *J* = 5.7 Hz, 1H, NH), 4.44 (*dd*, *J* = 11.2, 4.9 Hz, 1H, 3-H), 4.02 – 3.69 (*m*, 2H, 32-H_a, 32'-H_a), 3.69 – 3.56 (*m*, 2H, 30-H), 3.36 – 3.22 (*m*, 3H, 19-H, 31-H), 3.19 – 2.76 (*m*, 2H, 32-H_b, 32'-H_b), 2.19 (*td*, *J* = 12.0, 4.1 Hz, 1H, 13-H), 2.14 (*s*, 3H, 19-H), 2.14 – 2.03 (*m*, 6H, 33-H, 33'-H, 16-H_a, 18-H), 2.02 (*s*, 3H, Ac), 2.00–1.87 (*m*, 1H, 21-H_a), 1.87 – 1.77 (*m*, 1H, 22-H_a), 1.67 – 1.11 (*m*, 15H, 1-H_a, 2-H_a, 2-H_b, 16-H_b, 22-H_b, 6-H_a, 21-H_b, 11-H_a, 6-H_b, 7-H_a, 7-H_b, 15-H_a, 9-H, 11-H_b, 15-H_b), 1.10 – 0.91 (*m*, 3H, 12-H_a, 12-H_b, 1-H_b), 0.96 (*s*, 3H, 27-H), 0.87 (*s*, 3H, 26-H), 0.82 (*s*, 6H, 23-H, 25-H), 0.81 (*s*, 3H, 24-H), 0.79 – 0.75 (*m*, 1H, 5-H) ppm; ^{13}C NMR (126 MHz, $CDCl_3$): *δ* = 212.6 (C-20), 177.9 (C-28), 171.0 (Ac), 81.0 (C-3), 55.8 (C-17), 55.6 (C-31), 55.5 (C-5), 55.0 (C-32, C-32'), 51.3 (C-19), 50.5 (C-9), 50.2 (C-18), 42.3 (C-14), 40.8 (C-8), 38.5 (C-1), 37.9 (C-4), 37.7 (C-22), 37.3 (C-10), 37.0 (C-13), 36.0 (C-30), 34.3 (C-7), 32.4 (C-16), 30.1 (C-29), 29.6 (C-15), 28.6 (C-21), 28.1 (C-23), 27.3 (C-12), 23.8 (C-2), 23.4 (C-33, C-33'), 21.4 (Ac), 21.1 (C-11), 18.3 (C-6), 16.6 (C-24), 16.3 (C-25), 16.2 (C-26), 14.8 (C-27) ppm; MS (ESI, MeOH): *m/z* = 597 (100%, $[M+H]^+$); analysis calcd for $C_{35}H_{58}N_2O_4$ (570.44): C 74.45, H 10.13, N 4.69; found: C 74.30, H 10.41, N 4.47.

*(3β)-N-(2-Pyrrolidin-1-ylethyl)-3-hydroxy-urs-12-en-28-amide (**36**)*

Compound **36** was prepared from **31** according to general procedure C. Column chromatography (SiO_2, $CHCl_3$/MeOH 95:5) gave **36** (yield: 84%); m.p. 262–266 °C (decomp.); $[\alpha]_D$ = +39.8° (*c* 0.445, MeOH); R_f = 0.40 ($CHCl_3$/MeOH 9:1); IR (KBr): ν = 3420*br s*, 2926*s*, 2670*s*, 2616*m*, 2488*m*, 2360*s*, 2342*m*, 1636*m*, 1526*m*, 1456*m*, 1386*m*, 1278*w*, 1244*w*, 1092*w*, 1046*m*, 998*m*, 668*m* cm^{-1}; ^{1}H NMR (500 MHz, $CDCl_3$): *δ* = 7.06 (*t*, *J* = 5.5 Hz, 1H, NH), 5.40 (*t*, *J* = 3.5 Hz, 1H, 12-H), 3.89 – 3.77 (*m*, 2H, 33-H_a, 33'-H_a), 3.76 – 3.66 (*m*, 1H, 31-H_a), 3.61 – 3.51 (*m*, 1H, 31-H_b), 3.27 – 3.14 (*m*, 3H, 3-H, 32-H), 2.89 – 2.77 (*m*, 2H, 33-H_b, 33'-H_b), 2.26 – 2.17 (*m*, 2H, 34-H_a, 34'-H_a), 2.14 (*d*, *J* = 11.0 Hz, 1H, 18-H), 2.12 – 1.97 (*m*, 3H, 34-H_b, 34'-H_b, 16-H_a), 1.94 (*dd*, *J* = 8.8, 3.4 Hz, 2H, 11-H_a, 11-H_b), 1.81 – 1.22 (*m*, 15H, 22-H_a, 16-H_b, 15-H_a, 1-H_a, 2-H_a, 2-H_b, 6-H_a, 9-H, 22-H_b, 7-H_a, 21-H_a, 19-H, 6-H_b, 21-H_b, 7-H_b), 1.08 (*s*, 3H, 27-H), 1.07 – 0.96 (*m*, 3H, 15-H_b, 20-H, 1-H_b), 0.98 (*s*, 3H, 23-H), 0.94 (*d*, *J* = 6.3 Hz, 3H, 30-H), 0.91 (*s*, 3H, 25-H), 0.88 (*d*, *J* = 6.4 Hz, 3H, 29-H), 0.78

(*s*, 3H, 24-H), 0.73 (*s*, 3H, 26-H), 0.73 – 0.69 (*m*, 1H, 5-H) ppm; ^{13}C NMR (126 MHz, $CDCl_3$): δ = 179.6 (C-28), 138.9 (C-13), 126.2 (C-12), 79.2 (C-3), 55.4 (C-32), 55.3 (C-5), 54.9 (C-33), 54.6 (C-33'), 52.9 (C-18), 47.9 (C-17), 47.7 (C-9), 42.4 (C-14), 39.8 (C-19), 39.7 (C-8), 38.9 (C-1), 38.9 (C-20), 38.7 (C-4), 37.4 (C-22), 37.1 (C-10), 36.1 (C-31), 32.9 (C-7), 31.0 (C-21), 28.3 (C-23), 28.0 (C-15), 27.4 (C-2), 24.7 (C-16), 23.6 (C-27), 23.5 (C-34, C-34'), 23.4 (C-11), 21.4 (C-30), 18.5 (C-6), 17.3 (C-29), 17.2 (C-26), 15.8 (C-24), 15.6 (C-25) ppm; MS (ESI, MeOH): *m/z* = 553 (100%, $[M+H]^+$); analysis calcd for $C_{36}H_{60}N_2O_2$ (552.89): C 78.21, H 10.94, N 5.07; C 78.02, H 11.09, N 4.83.

*(3β)-N-(2-Pyrrolidin-1-ylethyl)-3-hydroxy-lup-20(29)-en-28-amide (**39**)*

Compound **39** was prepared from **34** according to general procedure C. Column chromatography (SiO_2, $CHCl_3$/MeOH 95:5) gave **39** (yield: 80%); m.p. 253–256 °C (decomp.); $[\alpha]_D$ = –14.7° (*c* 0.320, MeOH); R_f = 0.40 ($CHCl_3$/MeOH 9:1); IR (KBr): ν = 3426*br s*, 2942*s*, 2866*s*, 2696*m*, 2620*m*, 2500*m*, 1638*s*, 1544*m*, 1450*m*, 1376*m*, 1246*w*, 1196*w*, 1046*m*, 880*m* cm^{-1}; ^{1}H NMR (500 MHz, $CDCl_3$): δ = 7.54 (*t*, *J* = 5.7 Hz, 1H, NH), 4.73 – 4.71 (*m*, 1H, 29-H_a), 4.59 – 4.57 (*m*, 1H, 29-H_b), 3.91 – 3.79 (*m*, 2H, 33-H_a, 33'-H_a), 3.78 – 3.61 (*m*, 2H, 31-H), 3.24 – 3.15 (*m*, 3H, 32-H, 3-H), 3.07 (*ddd*, *J* = 10.9, 10.9, 4.2 Hz, 1H, 19-H), 2.89 – 2.78 (*m*, 2H, 33-H_b, 33'-H_b), 2.42 (*ddd*, J = 12.6, 12.6, 3.6 Hz, 1H, 13-H), 2.31 – 2.18 (*m*, 3H, 16-H_a, 34-H_a, 34'-H_a), 2.15 – 2.05 (*m*, 2H, 34-H_b, 34'-H_b), 1.96 – 1.78 (*m*, 2H, 22-H_a, 21-H_a), 1.67 (*s*, 3H, 30-H), 1.73 – 1.14 (*m*, 17H, 12-H_a, 1-H_a, 2-H_a, 2-H_b, 18-H, 16-H_b, 6-H_a, 22-H_b, 11-H_a, 6-H_b, 21-H_b, 7-H_a, 7-H_b, 15-H_a, 9-H, 11-H_b, 15-H_b), 1.01 – 0.92 (*m*, 1H, 12-H_b), 0.96 (*s*, 6H, 23-H, 27-H), 0.91 (*s*, 3H, 26-H), 0.89 – 0.81 (*m*, 1H, 1-H_b), 0.81 (*s*, 3H, 25-H), 0.75 (*s*, 3H, 24-H), 0.70 – 0.65 (*m*, 1H, 5-H) ppm; ^{13}C NMR (126 MHz, $CDCl_3$): δ = 177.9 (C-28), 151.1 (C-20), 109.5 (C-29), 79.1 (C-3), 56.6 (C-32), 56.1 (C-17), 55.5 (C-5), 54.8 (C-33, C-33'), 50.8 (C-9), 50.4 (C-18), 47.0 (C-19), 42.6 (C-14), 40.9 (C-8), 39.0 (C-4), 38.9 (C-1), 38.2 (C-22), 37.9 (C-13), 37.4 (C-10), 35.7 (C-31), 34.6 (C-7), 33.2 (C-16), 31.1 (C-21), 29.7 (C-15), 28.1 (C-23), 27.6 (C-2), 25.8 (C-12), 23.5 (C34, C-34'), 21.1 (C-11), 19.6 (C-30), 18.5 (C-6), 16.4 (C-26), 16.3 (C-25), 15.5 (C-24), 14.8 (C-27) ppm; MS (ESI, MeOH): *m/z* = 553 (100%, $[M+H]^+$); analysis calcd for $C_{36}H_{60}N_2O_2$ (552.89): C 78.21, H 10.94, N 5.07; found: C 78.00, H 11-09, N 4.81.

*(3β)-N-(2-Pyrrolidin-1-ylethyl)-3-hydroxy-20-oxo-30-norlupan-28-amide (**40**)*

Compound **40** was prepared from **35** according to general procedure C. Column chromatography (SiO_2, $CHCl_3$/MeOH 9:1) gave **40** (yield: 76%); m.p. 170–173 °C; $[\alpha]_D$ = –

29.3° (*c* 0.415, MeOH); R_f = 0.27 (silica gel, $CHCl_3$/MeOH 9:1); IR (KBr): ν = 3422*br s*, 2944*s*, 2868*m*, 1704*m*, 1646*s*, 1530*m*, 1354*s*, 1246*m*, 1198*m*, 1082*w*, 1034*m*, 752*m* cm^{-1}; ^{1}H NMR (500 MHz, $CDCl_3$): δ = 7.43 (*t*, *J* = 5.5 Hz, 1H, NH), 3.95 – 3.74 (*m*, 2H, 32-H_a, 32'-H_a), 3.67 – 3.59 (*m*, 2H, 30-H), 3.35 – 3.25 (*m*, 3H, 19-H, 31-H), 3.16 (*dd*, *J* = 11.3, 4.7 Hz, 1H, 3-H), 3.05 – 2.86 (*m*, 2H, 32-H_b, 32'-H_b), 2.19 (*td*, *J* = 12.0, 4.1 Hz, 1H, 13-H), 2.13 (*s*, 3H, 29-H), 2.14 – 1.87 (*m*, 7H, 33-H, 33'-H, 16-H_a, 18-H, 21-H_a), 1.85 – 1.78 (*m*, 1H, 22-H_a), 1.68 – 1.12 (*m*, 15H, 1-H_a, 12-H_a, 12-H_b, 16-H_b, 22-H_b, 6-H_a, 21-H_b, 11-H_a, 6-H_b, 7-H_a, 7-H_b, 15-H_a, 9-H, 11-H_b, 15-H_b), 0.96 (*s*, 3H, 27-H), 1.09 – 0.82 (*m*, 3H, 2-H_a, 2-h_b, 1-H_b), 0.94 (*s*, 3H, 23-H), 0.86 (*s*, 3H, 26-H), 0.78 (*s*, 3H, 25-H), 0.72 (*s*, 3H, 24-H), 0.68–0.64 (*m*, 1H, 5-H) ppm; ^{13}C NMR (126 MHz, $CDCl_3$): δ = 212.7 (C-20), 178.0 (C-28), 78.9 (C-3), 55.8 (C-17), 55.5 (C-31), 55.4 (C-5), 55.0 (C-32, C-32'), 51.3 (C-19), 50.6 (C-9), 50.2 (C-18), 42.3 (C-14), 40.8 (C-8), 39.0 (C-4), 38.8 (C-1), 37.7 (C-22), 37.3 (C-10), 37.0 (C-13), 36.0 (C-30), 34.4 (C-7), 32.4 (C-16), 30.2 (C-29), 29.6 (C-15), 28.6 (C-21), 28.1 (C-23), 27.5 (C-12), 27.3 (C-2), 23.4 (C-33, C-33'), 21.1 (C-11), 18.4 (C-6), 16.3 (C-25), 16.2 (C-26), 15.5 (C-24), 14.8 (C-27) ppm; MS (ESI, MeOH): *m/z* = 555 (100%, $[M+H]^+$); analysis calcd for $C_{35}H_{58}N_2O_3$ (554.44): C 75.76, H 10.54, N 5.05; found: C 75.51, H 10.70, N 4.81.

*(3β)-N-(2-Piperidin-1-ylethyl)-3-acetyloxy-urs-12-en-28-amide (**41**)*

Compound **41** was prepared from **6** according to general procedure B using 1-(2-aminoethyl)piperidine as amino compound. Column chromatography (SiO_2, $CHCl_3$/MeOH 95:5) gave **41** (yield: 83%); m.p. 124–127 °C; $[\alpha]_D$ = +34.6° (*c* 0.365, $CHCl_3$); R_f = 0.26 ($CHCl_3$/MeOH 95:5); IR (KBr): ν = 3424*br s*, 2936*s*, 2872*m*, 2854*m*, 1736*s*, 1638*s*, 1508*m*, 1456*m*, 1370*m*, 1246*s*, 1154*w*, 1128*w*, 1092*w*, 1028*m* cm^{-1}; ^{1}H NMR (400 MHz, $CDCl_3$): δ = 6.56 – 6.51 (*m*, 1H, NH), 5.30 (*t*, *J* = 3.6 Hz, 1H, 12-H), 4.49 (*dd*, *J* = 10.4, 5.8 Hz, 1H, 3-H), 3.41 – 3.29 (*m*, 1H, 31-H_a), 3.24 – 3.12 (*m*, 1H, 31-H_b), 2.49 – 2.27 (*m*, 6H, 32-H, 33-H, 33'-H), 2.03 (*s*, 3H, Ac), 2.01 – 1.80 (*m*, 5H, 16-H_a, 11-H_a, 11-H_b, 18, 22-H_a), 1.78 – 1.22 (*m*, 20H, 16-H_b, 15-H_a, 1-H_a, 2-H_a, 2-H_b, 34-H, 34'-H, 9-H, 6-H_a, 21-H_a, 7-H_a, 35-H, 22-H_b, 19-H, 6-H_b, 21-H_b, 7-H_b), 1.08 (*s*, 3H, 27-H), 1.14 – 0.96 (*m*, 3H, 1-H_b, 15-H_b, 20-H), 0.95 – 0.93 (*m*, 3H, 30-H), 0.93 (*s*, 3H, 25-H), 0.88 (*d*, *J* = 6.5 Hz, 3H, 29-H), 0.86 (*s*, 3H, 23-H), 0.85 (*s*, 3H, 24-H), 0.84 – 0.79 (*m*, 1H, 5-H), 0.77 (*s*, 3H, 26-H) ppm; ^{13}C NMR (101 MHz, $CDCl_3$): δ = 178.0 (C-28), 171.1 (Ac), 139.5 (C-13), 125.6 (C-12), 81.0 (C-3), 57.2 (C-32), 55.4 (C-5), 54.5 (C-33, C-33'), 54.0 (C-18), 47.9 (C-17), 47.6 (C-9), 42.5 (C-14), 39.9 (C-19), 39.7 (C-8), 39.2 (C-20), 38.4 (C-1), 37.8 (C-4), 37.5 (C-22), 37.0 (C-10), 36.0 (C-31), 32.9 (C-7), 31.1 (C-21), 28.2 (C-23), 28.0 (C-15), 26.2 (C-34, C-34'), 24.9 (C-16), 24.5 (C-35), 23.7 (C-2),

23.5 (C-11), 23.4 (C-27), 21.4 (Ac), 21.4 (C-30), 18.3 (C-6), 17.5 (C-29), 17.1 (C-26), 16.9 (C-24), 15.7 (C-25) ppm; MS (ESI, MeOH): *m/z* = 609 (100%, $[M+H]^+$); analysis calcd for $C_{39}H_{64}N_2O_3$ (608.95): C 76.92, H 10.59, N 4.60; found: C 76.77, H 10.79, N 4.41.

*(3β)-N-(2-Piperidin-1-ylethyl)-3-acetyloxy-lup-20(29)-en-28-amide (**44**)*

Compound **44** was prepared from **9** according to general procedure B using 1-(2-aminoethyl)piperidine as amino compound. Column chromatography (SiO_2, $CHCl_3$/MeOH 95:5) gave **44** (yield: 81%); m.p. 124–127 °C; $[\alpha]_D$ = +14.1° (*c* 0.340, $CHCl_3$); R_f = 0.25 ($CHCl_3$/MeOH 95:5); IR (KBr): ν = 3424*br s*, 2942*s*, 2968*m*, 1736*s*, 1638*s*, 1508*m*, 1452*m*, 1376*m*, 1246*s*, 1154*w*, 1128*w*, 1028*m* cm^{-1}; ^1H NMR (400 MHz, $CDCl_3$): δ = 6.52 – 6.39 (*m*, 1H, NH), 4.75 – 4.70 (*m*, 1H, 29-H_a), 4.61 – 4.56 (*m*, 1H, 29-H_b), 4.46 (*dd*, *J* = 9.8, 6.5 Hz, 1H, 3-H), 3.41 – 3.25 (*m*, 2H, 31-H_a, 31-H_b), 3.08 (*ddd*, *J* = 11.1, 10.9, 3.9 Hz, 1H, 19-H), 2.53 – 2.40 (*m*, 6H, 32-H, 33-H, 33'-H), 2.35 (*ddd*, *J* = 12.4, 12.3, 3.6 Hz, 1H, 13-H), 2.03 (*s*, 3H, Ac), 2.12 – 1.89 (*m*, 2H, 16-H_a, 21-H_a), 1.83 – 1.74 (*m*, 1H, 22-H_a), 1.68 (*s*, 3H, 30-H), 1.72 – 1.54 (*m*, 9H, 12-H_a, 1-H_a, 2-H_a, 2-H_b, 18-H, 34-H, 34'-H), 1.55 – 1.15 (*m*, 13H, 16-H_b, 15-H_a, 6-H_a, 35-H, 11-H_a, 22-H_b, 21-H_b, 6-H_b, 7-H_a, 7-H_b, 9-H, 11-H_b), 1.15 – 1.09 (*m*, 1H, 15-H_b), 1.08 – 0.93 (*m*, 2H, 12-H_b, 1-H_b), 0.96 (*s*, 3H, 27-H), 0.92 (*s*, 3H, 26-H), 0.83 (*s*, 6H, 25-H, 23-H), 0.82 (*s*, 3H, 24-H), 0.80 – 0.74 (*m*, 1H, 5-H) ppm; ^{13}C NMR (101 MHz, $CDCl_3$): δ = 176.3 (C-28), 171.1 (Ac), 151.1 (C-20), 109.5 (C-29), 81.1 (C-3), 57.1 (C-32), 56.0 (C-17), 55.6 (C-5), 54.3 (C-33, C-33'), 50.6 (C-9), 50.0 (C-18), 47.2 (C-19), 42.7 (C-14), 40.9 (C-8), 38.5 (C-1), 38.5 (C-22), 38.1 (C-13), 37.9 (C-4), 37.3 (C-10), 35.7 (C-31), 34.5 (C-7), 33.8 (C-16), 31.1 (C-21), 29.6 (C-15), 28.1 (C-23), 26.1 (C-34, C-34'), 25.8 (C-12), 24.4 (C-35), 23.8 (C-2), 21.4 (Ac), 21.1 (C-11), 19.6 (C-30), 18.4 (C-6), 16.6 (C-24), 16.4 (C-25), 16.3 (C-26), 14.8 (C-27) ppm; MS (ESI, MeOH): *m/z* = 609 (100%, $[M+H]^+$); analysis calcd for $C_{39}H_{64}N_2O_3$ (608.95): C 76.92, H 10.59, N 4.60; found: C 76.77, H 10.79, N 4.41.

*(3β)-N-(2-Piperidin-1-ylethyl)-3-hydroxy-urs-12-en-28-amide (**46**)*

Compound **46** was prepared from **41** according to general procedure C. Column chromatography (SiO_2, $CHCl_3$/MeOH 95:5) gave **46** (yield: 93%); m.p. 120–124 °C; $[\alpha]_D$ = +40.5° (*c* 0.350, $CHCl_3$); R_f = 0.23 ($CHCl_3$/MeOH 95:5); IR (KBr): ν = 3416*br s*, 2934*s*, 2870*m*, 2854*m*, 1636*s*, 1512*m*, 1456*m*, 1378*m*, 1358*w*, 1304*w*, 1272*w*, 1256*w*, 1156*w*, 1130*w*, 1092*w*, 1046*m*, 998*m*, 754*m* cm^{-1}; ^1H NMR (400 MHz, $CDCl_3$): δ = 6.62 – 6.54 (*m*, 1H, NH), 5.31 (*t*, J = 3.6 Hz, 1H, 12-H), 3.42 – 3.32 (*m*, 1H, 31-H_a), 3.25 – 3.16 (*m*, 2H, 3-H, 31-H_b), 2.50 – 2.35 (*m*, 6H, 32-H, 33-H, 33'-H), 2.02 – 1.80 (*m*, 5H, 16-H_a, 11-H_a, 11-H_b, 18-H, 22-

H_a), 1.78 – 1.20 (*m*, 20H, 16-H_b, 15-H_a, 1-H_a, 34-H, 34'-H, 35-H, 9-H, 6-H_a, 21-H_a, 7-H_a, 2-H_a, 2-H_b, 22-H_b, 19-H, 6-H_b, 21-H_b, 7-H_b), 1.08 (*s*, 3H, 27-H), 1.07 – 0.97 (*m*, 3H, 15-H_b, 1-H_b, 20-H), 0.98 (*s*, 3H, 23-H), 0.96 – 0.93 (*m*, 3H, 30-H), 0.91 (*s*, 3H, 25-H), 0.87 (*d*, J = 6.4 Hz, 3H, 29-H), 0.77 (*s*, 6H, 24-H, 26-H), 0.74 – 0.69 (*m*, 1H, 5-H) ppm; ^{13}C NMR (101 MHz, $CDCl_3$): δ = 178.0 (C-28), 139.4 (C-13), 125.8 (C-12), 79.1 (C-3), 57.2 (C-32), 55.3 (C-5), 54.4 (C-33, C-33'), 53.9 (C-18), 47.7 (C-17), 47.7 (C-9), 42.5 (C-14), 39.9 (C-19), 39.7 (C-8), 39.2 (C-20), 38.9 (C-1), 38.8 (C-4), 37.5 (C-22), 37.1 (C-10), 35.9 (C-31), 32.9 (C-7), 31.1 (C-21), 28.3 (C-23), 28.0 (C-15), 27.3 (C-35), 26.0 (C-34, C-34'), 24.9 (C-16), 24.4 (C-2), 23.5 (C-11), 23.4 (C-27), 21.4 (C-30), 18.4 (C-6), 17.4 (C-29), 17.1 (C-26), 15.8 (C-24), 15.6 (C-25) ppm; MS (ESI, MeOH): *m/z* = 567 (100%, $[M+H]^+$); analysis calcd for $C_{37}H_{62}N_2O_2$ (566.92): C 78.39, H 11.02, N 4.94; found: C 78.11, H 11.19, N 4.80.

*(3β)-N-(2-Piperidin-1-ylethyl)-3-hydroxy-lup-20(29)-en-28-amide (**49**)*

Compound **49** was prepared from **44** according to general procedure C. Column chromatography (SiO_2, $CHCl_3$/MeOH 95:5) gave **49** (yield: 83%); m.p. 141–144 °C (decomp.); $[\alpha]_D$ = +4.9° (*c* 0.315, $CHCl_3$); R_f = 0.21 ($CHCl_3$/MeOH 95:5); IR (KBr): ν = 3424*br s*, 2940*s*, 2866*m*, 2364*w*, 1638*s*, 1508*m*, 1452*m*, 1376*m*, 1248*w*, 1194*w*, 1128*w*, 1046*m* cm^{-1}; ^{1}H NMR (400 MHz, $CDCl_3$): δ = 6.78 – 6.57 (*m*, 1H, NH), 4.76 – 4.69 (*m*, 1H, 29-H_a), 4.61 – 4.56 (*m*, 1H, 29-H_b), 3.48 – 3.31 (*m*, 2H, 31-H), 3.17 (*dd*, *J* = 11.1, 5.0 Hz, 1H, 3-H), 3.08 (*ddd*, *J* = 11.0, 10.8, 3.9 Hz, 1H, 19-H), 2.65 – 2.46 (*m*, 6H, 32-H, 33-H, 33'-H), 2.37 (*ddd*, *J* = 12.4, 12.3, 3.6 Hz, 1H, 13-H), 2.15 – 2.08 (*m*, 1H, 16-H_a), 2.00 – 1.88 (*m*, 1H, 21-H_a), 1.85 – 1.76 (*m*, 1H, 22-H_a), 1.68 (*s*, 3H, 30-H), 1.73 – 1.08 (*m*, 23H, 12-H_a, 35-H, 1-H_a, 18-H, 34-H, 34'-H, 6-H_a, 2-H_a, 2-H_b, 16-H_b, 15-H_a, 11-H_a, 22-H_b, 21-H_b, 6-H_b, 7-H_a, 7-H_b, 9-H, 11-H_b, 15-H_b), 1.08 – 0.95 (*m*, 1H, 12-H_b), 0.96 (*s*, 3H, 27-H), 0.95 (*s*, 3H, 23-H), 0.91 (*s*, 3H, 26-H), 0.91 – 0.82 (*m*, 1H, 1-H_b), 0.80 (*s*, 3H, 25-H), 0.74 (*s*, 3H, 24-H), 0.70 – 0.63 (*m*, 1H, 5-H) ppm; ^{13}C NMR (101 MHz, $CDCl_3$): δ = 176.6 (C-28), 151.1 (C-20), 109.5 (C-29), 79.1 (C-3), 57.3 (C-32), 56.1 (C-17), 55.5 (C-5), 54.3 (C-33, C-33'), 50.7 (C-9), 50.1 (C-18), 47.1 (C-19), 42.7 (C-14), 40.9 (C-8), 39.0 (C-4), 38.9 (C-1), 38.4 (C-22), 38.1 (C-13), 37.4 (C-10), 35.5 (C-31), 34.6 (C-7), 33.6 (C-16), 31.1 (C-21), 29.6 (C-15), 28.1 (C-23), 27.6 (C-34, C-34'), 25.8 (C-12), 25.5 (C-35), 24.0 (C-2), 21.1 (C-11), 19.6 (C-30), 18.5 (C-6), 16.3 (C-26), 16.2 (C-25), 15.5 (C-24), 14.8 (C-27) ppm; MS (ESI, MeOH): *m/z* = 567 (100%, $[M+H]^+$); analysis calcd for $C_{37}H_{62}N_2O_2$ (566.92): C 78.39, H 11.02, N 4.94; found: C 78.16, H 11.20, N 4.71.

2 Representative NMR spectra

Compound 12:

^{1}H NMR (400 MHz, $CDCl_3$):

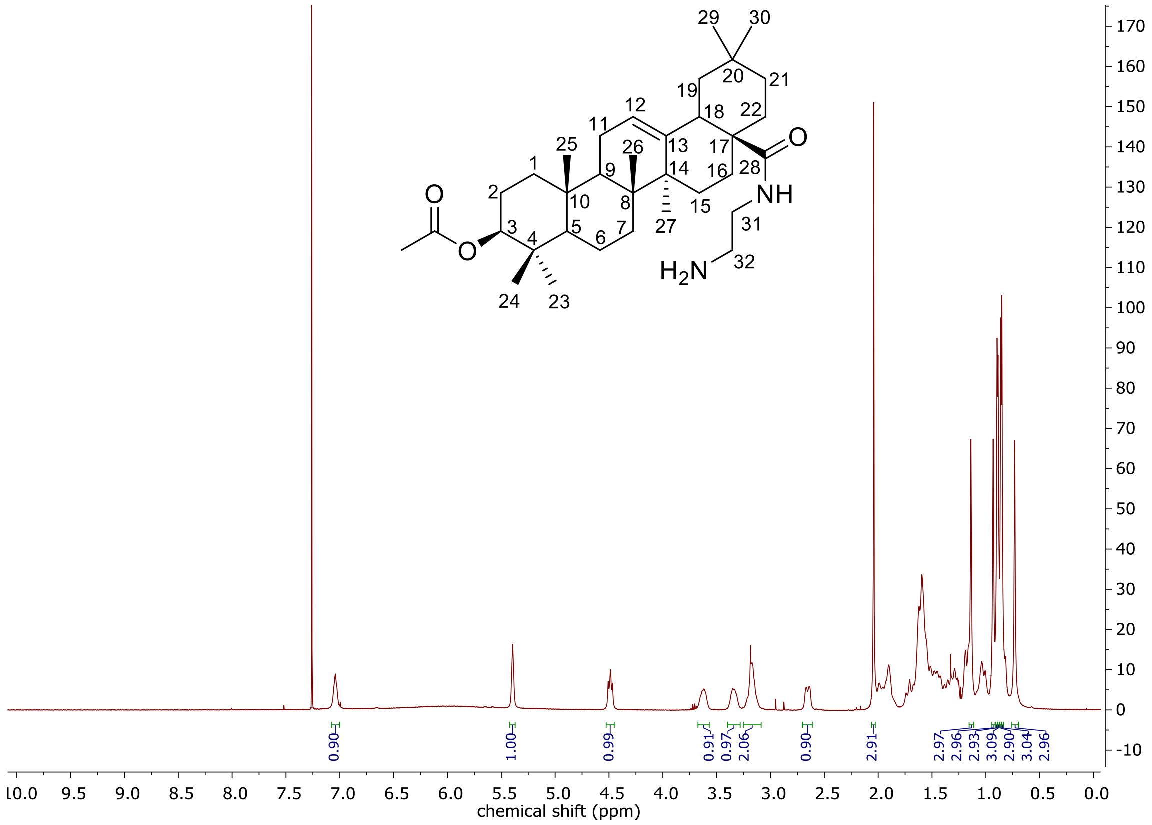

^{13}C APT NMR (101 MHz, $CDCl_3$):

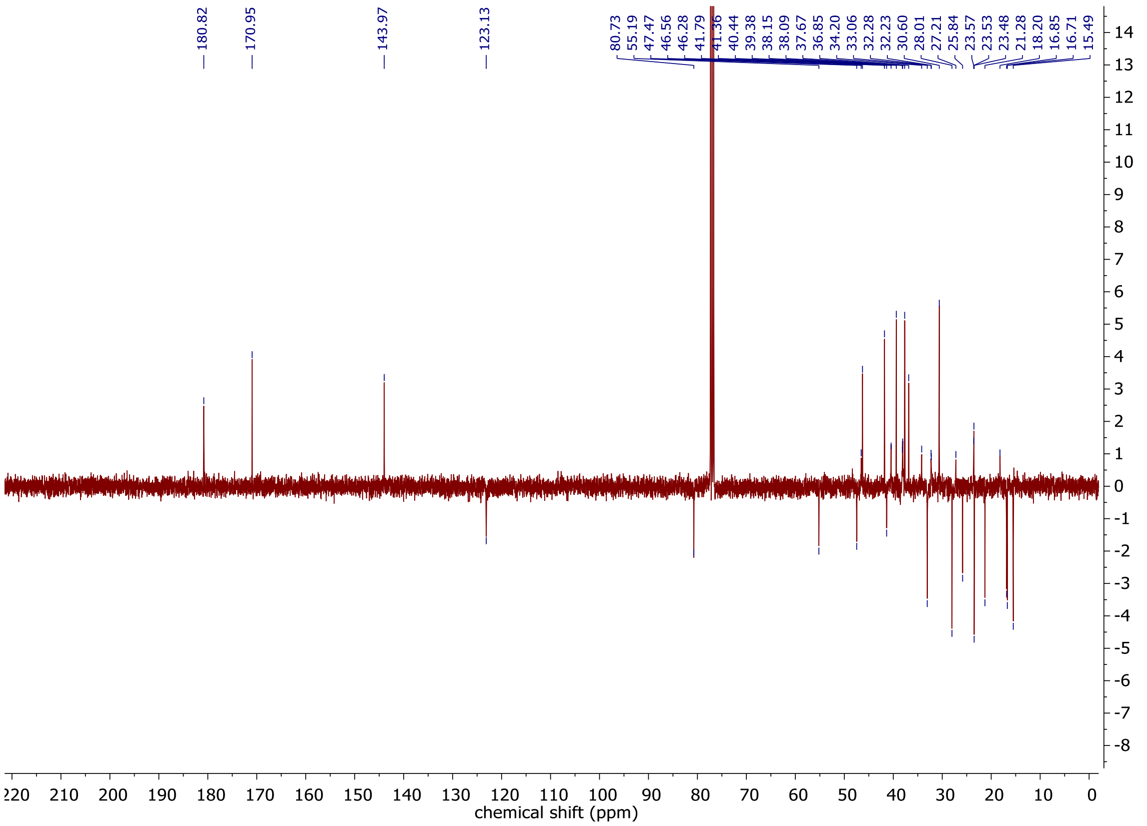

Compound 13:

^{1}H NMR (400 MHz, $CDCl_3$):

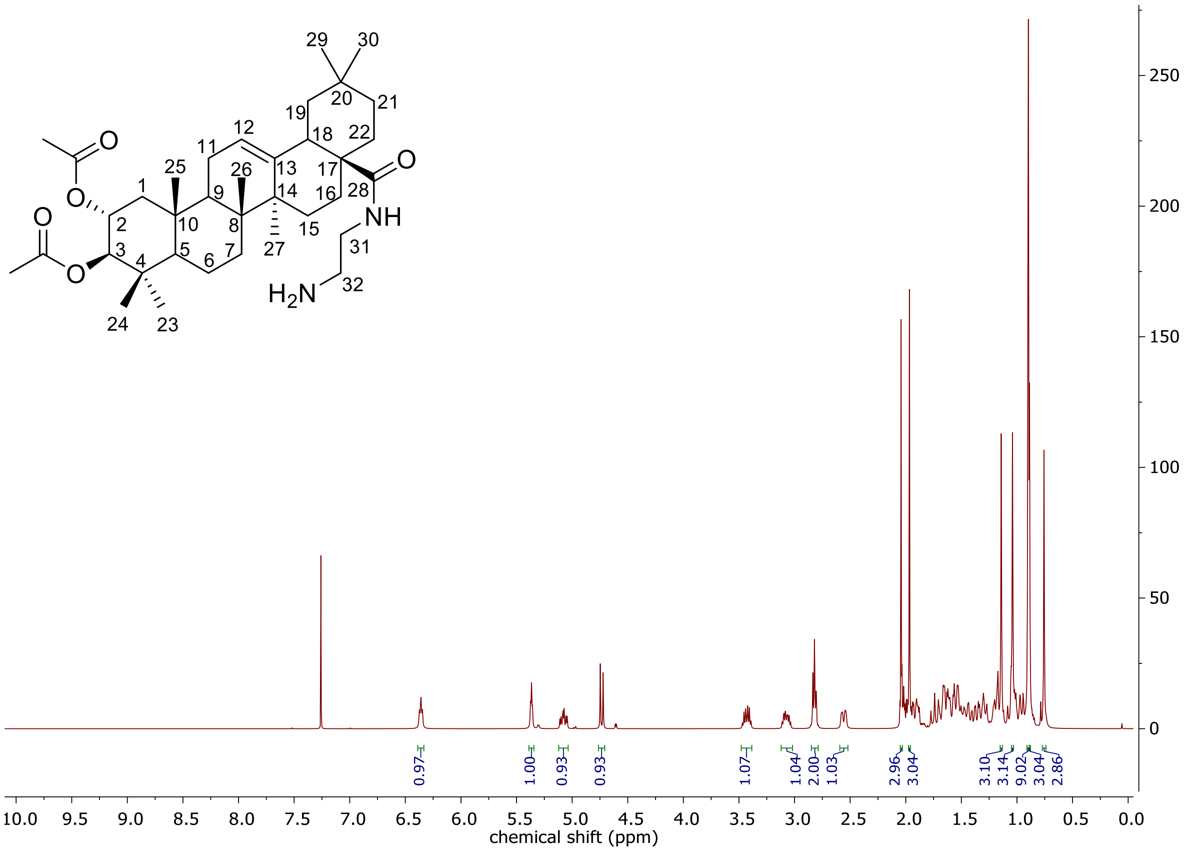

^{13}C APT NMR (101 MHz, $CDCl_3$):

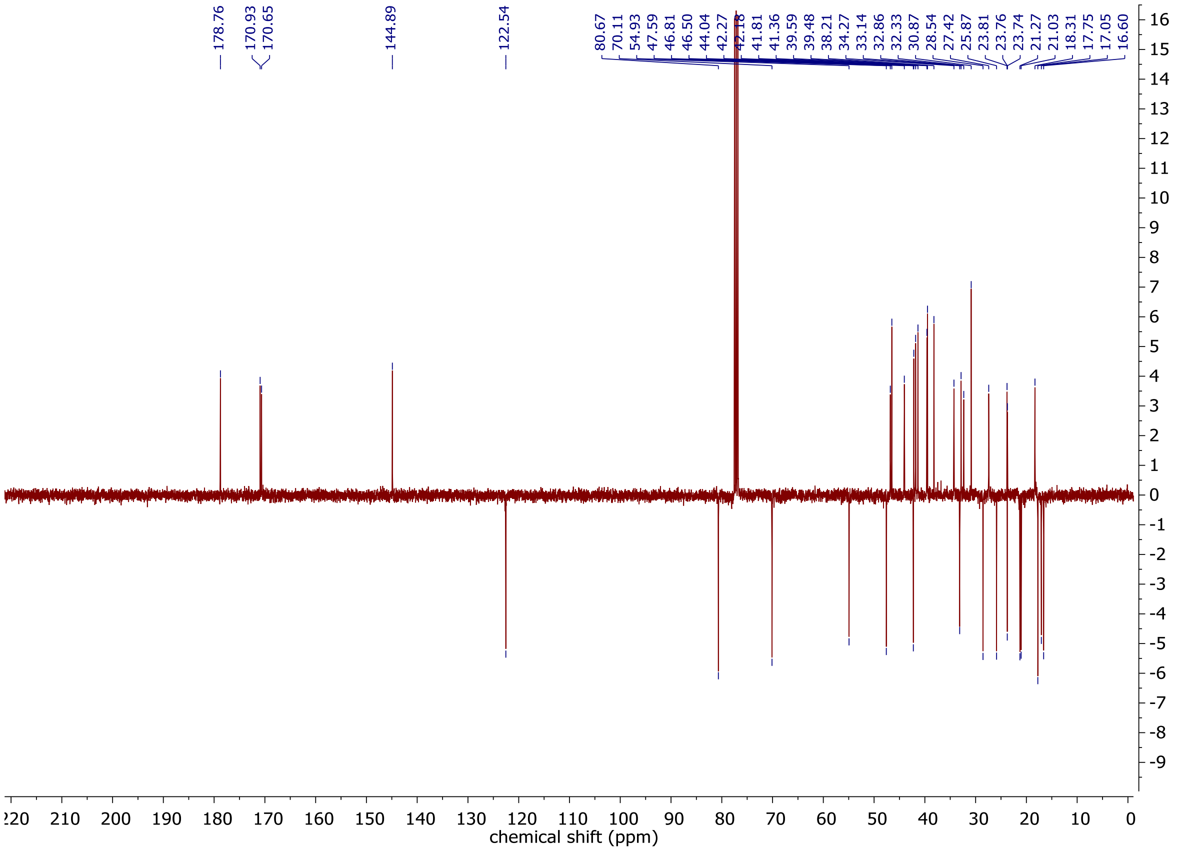

Compound 17:

^{1}H NMR (400 MHz, CD_3OD):

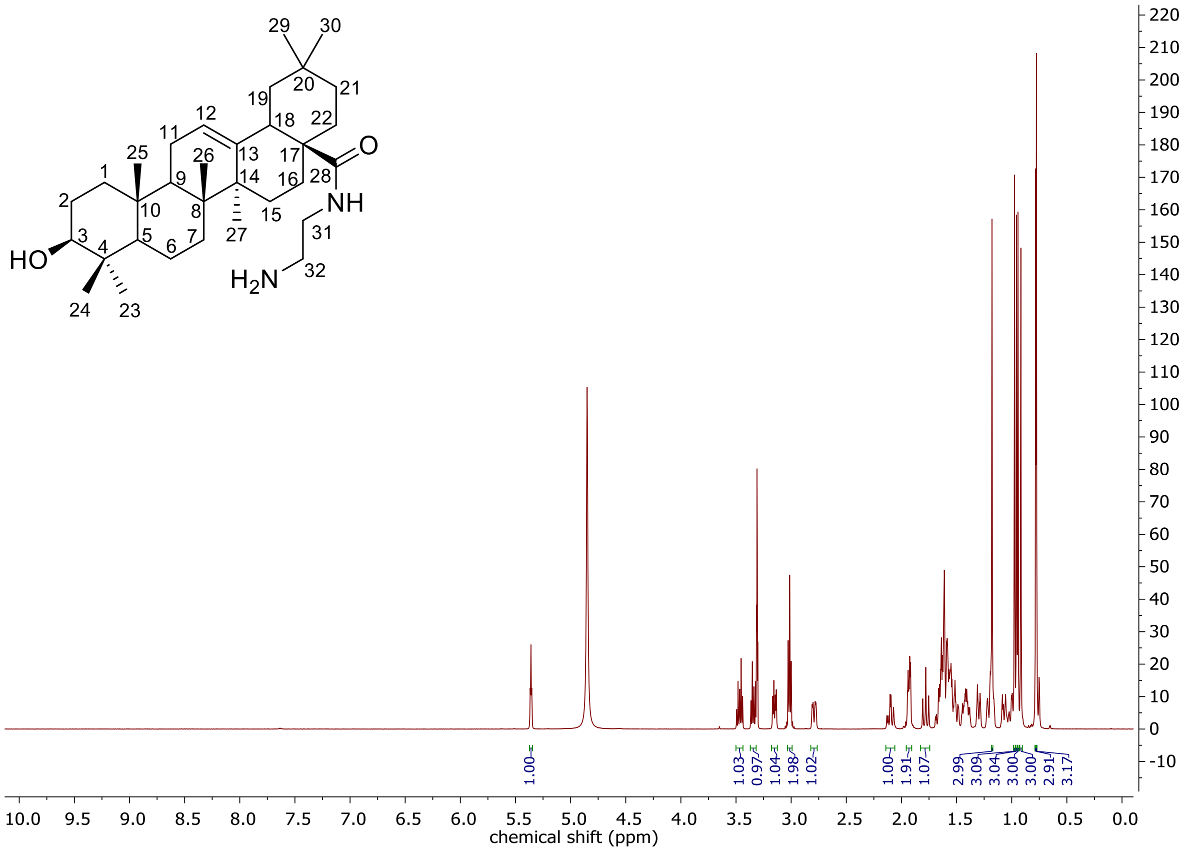

^{13}C APT NMR (101 MHz, CD_3OD):

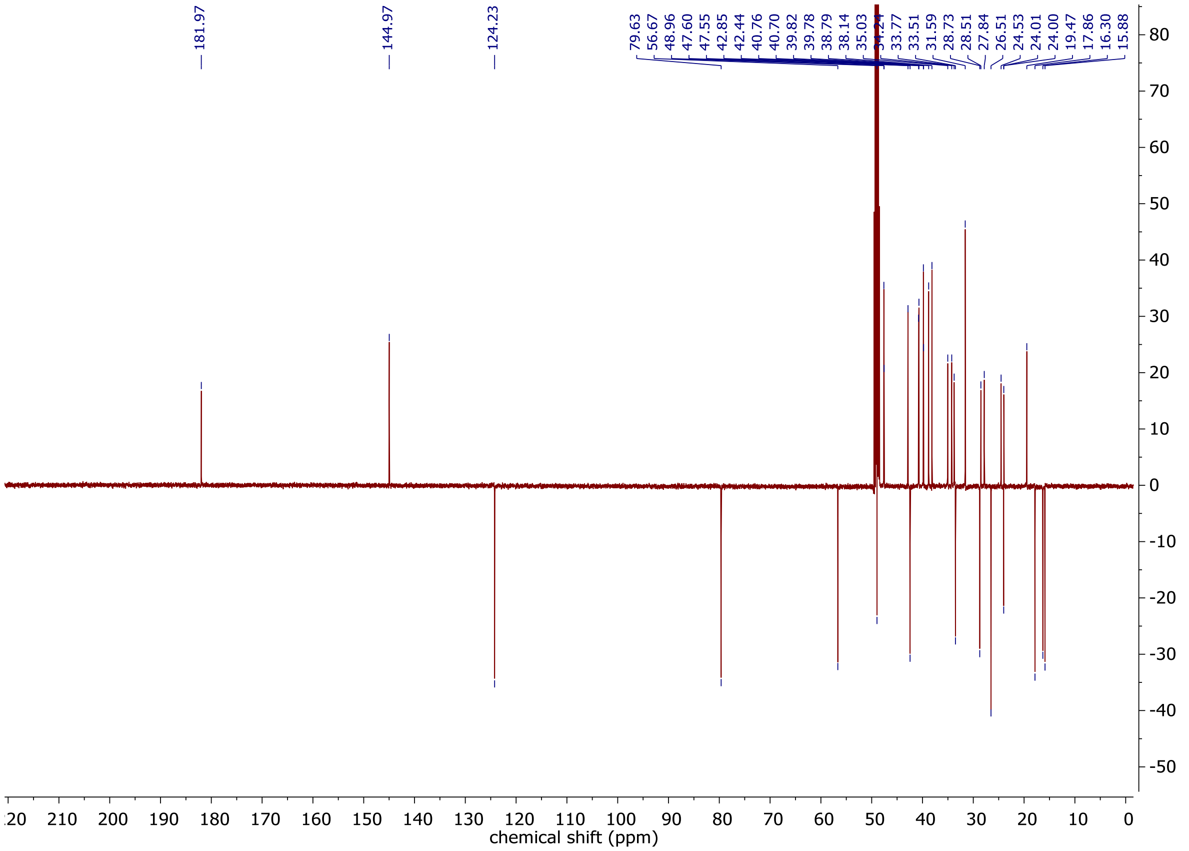

Compound 18:

^{1}H NMR (400 MHz, CD_3OD):

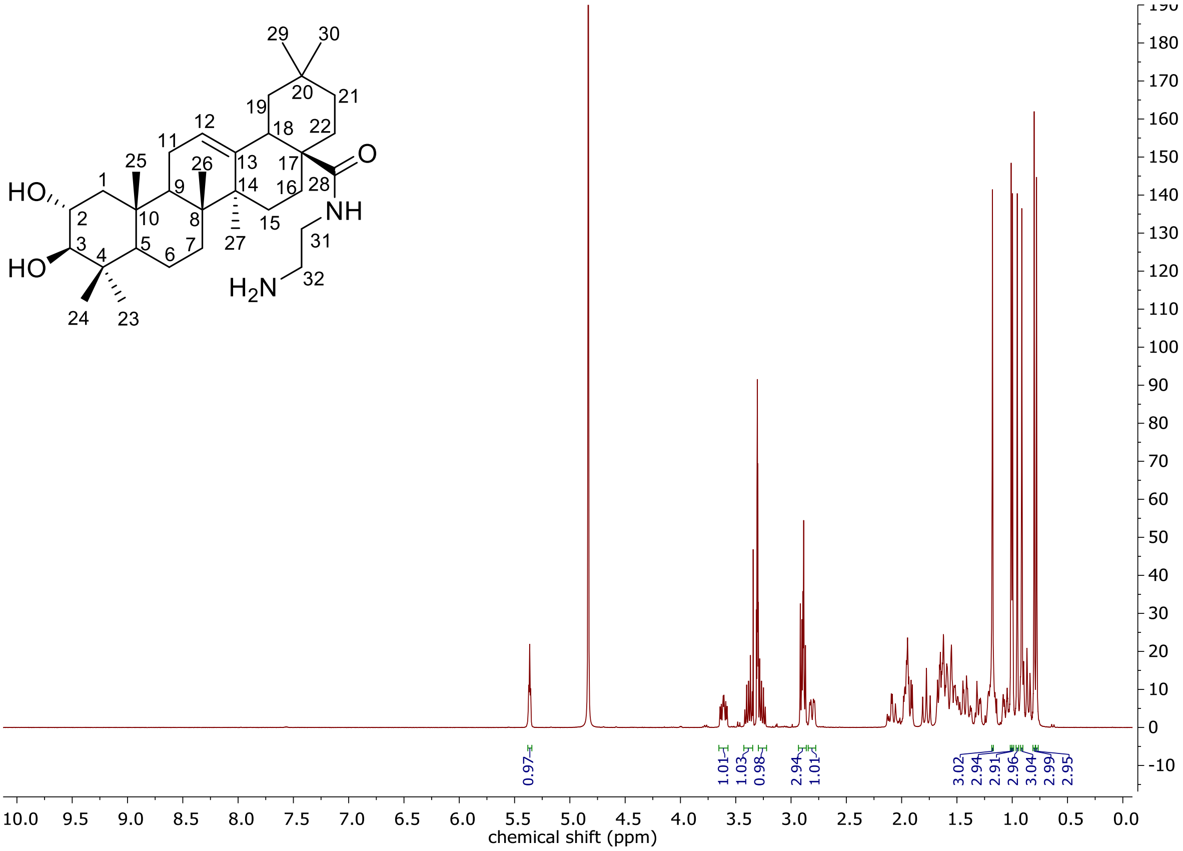

^{13}C APT NMR (101 MHz, CD_3OD):

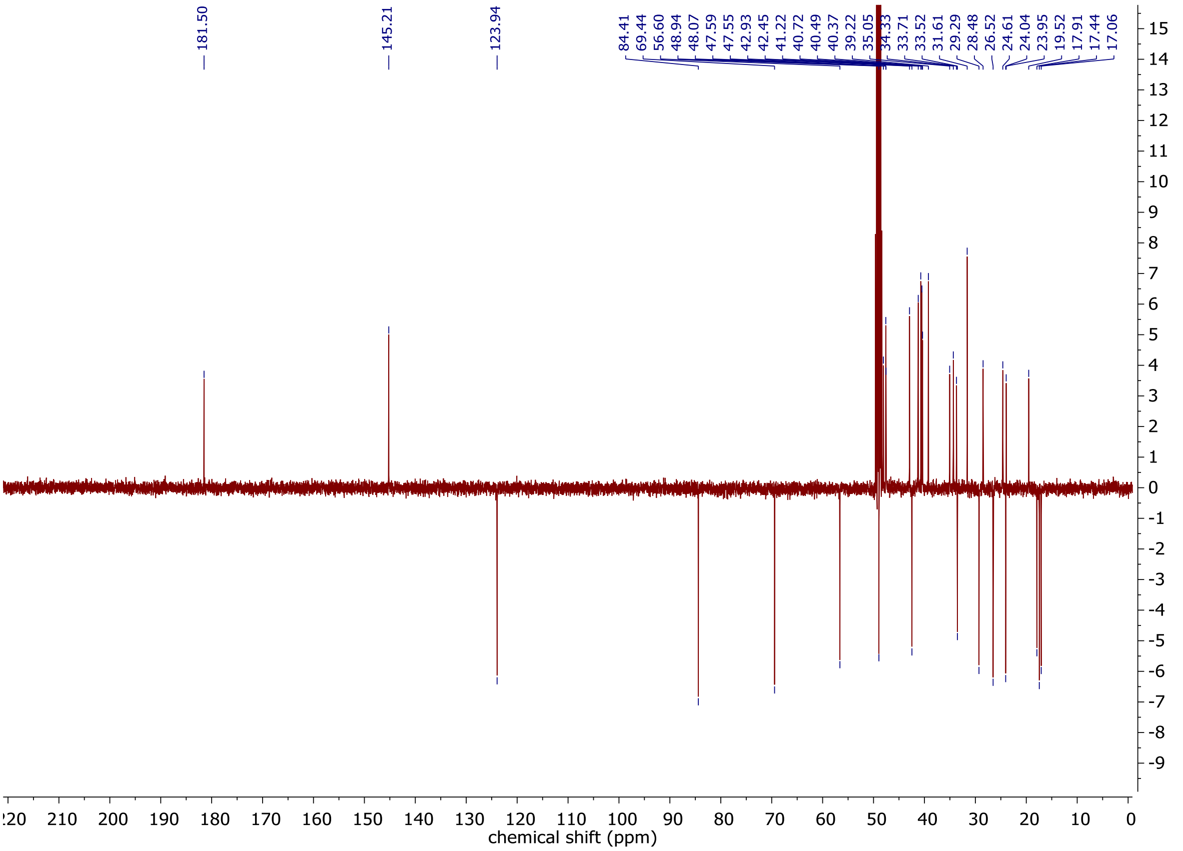

Compound 22:

^{1}H NMR (400 MHz, $CDCl_3$):

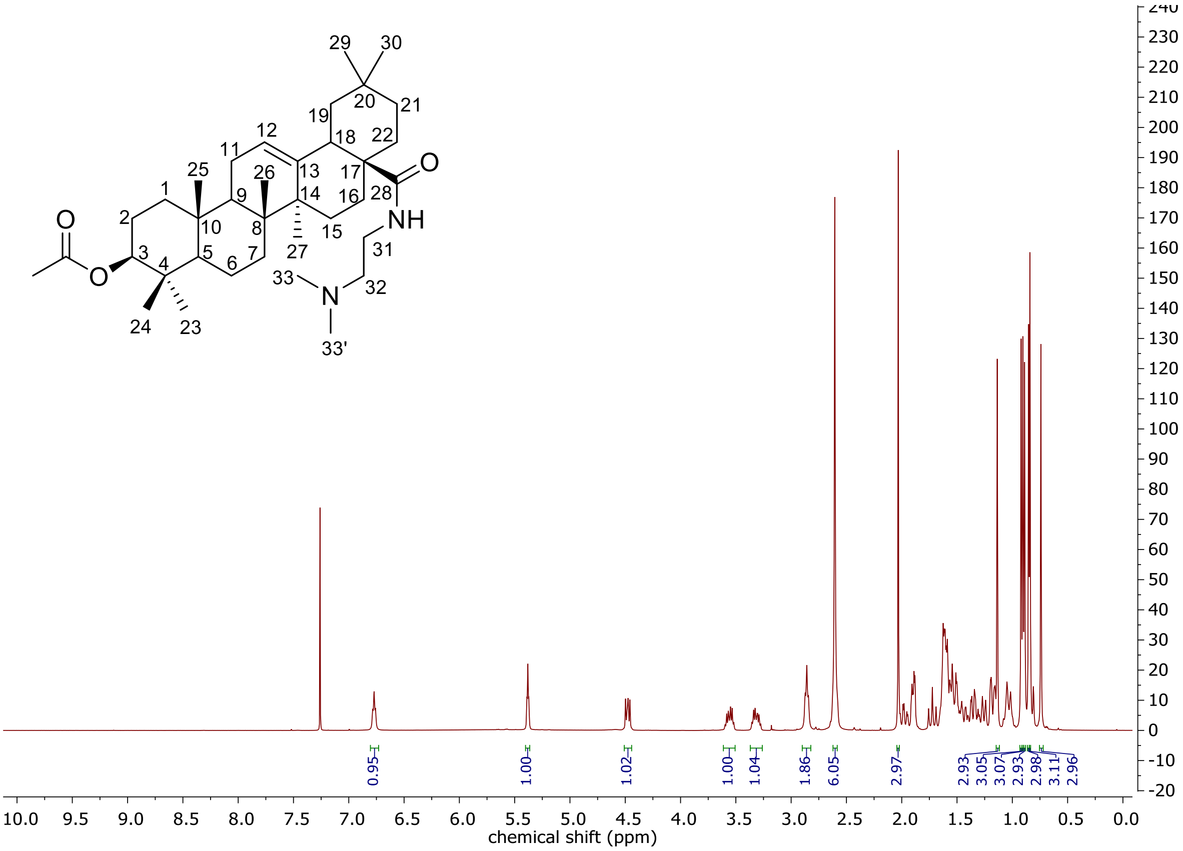

^{13}C APT NMR (101 MHz, $CDCl_3$):

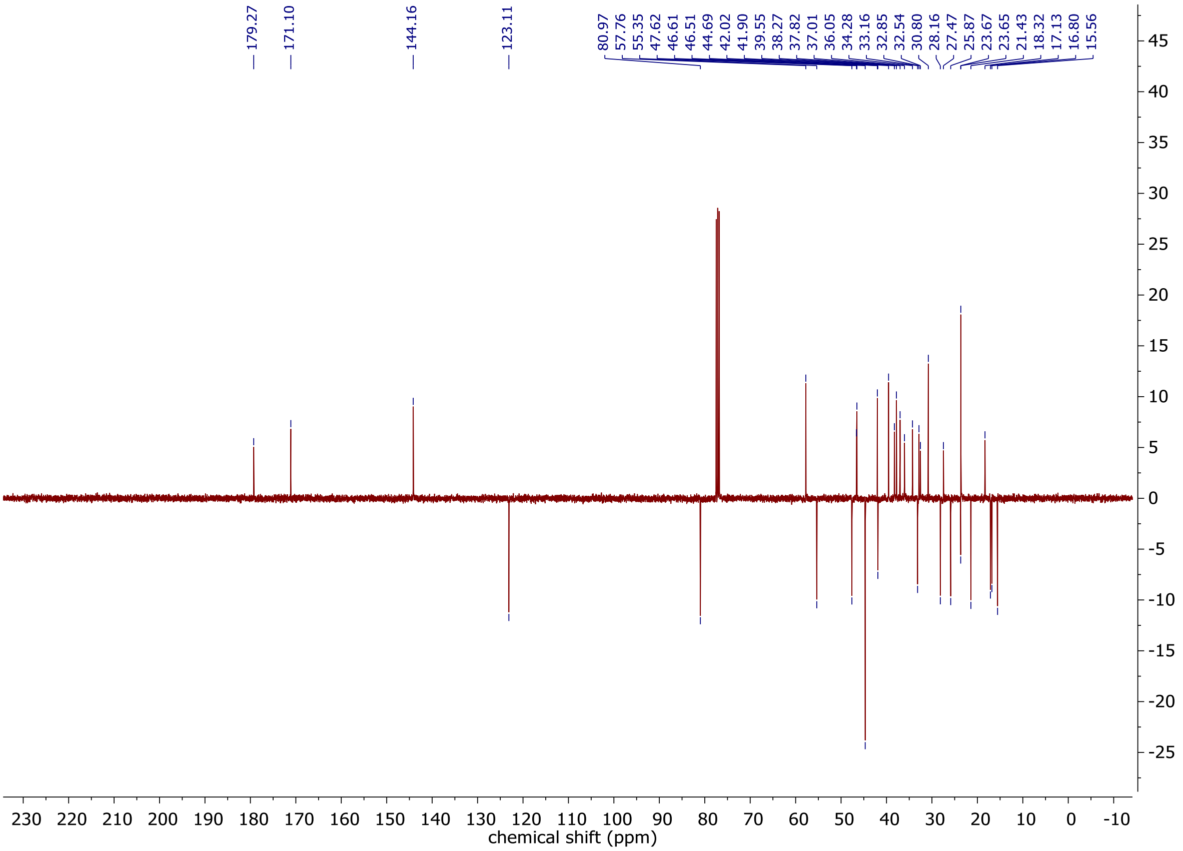

Compound 23:

^{1}H NMR (400 MHz, $CDCl_3$):

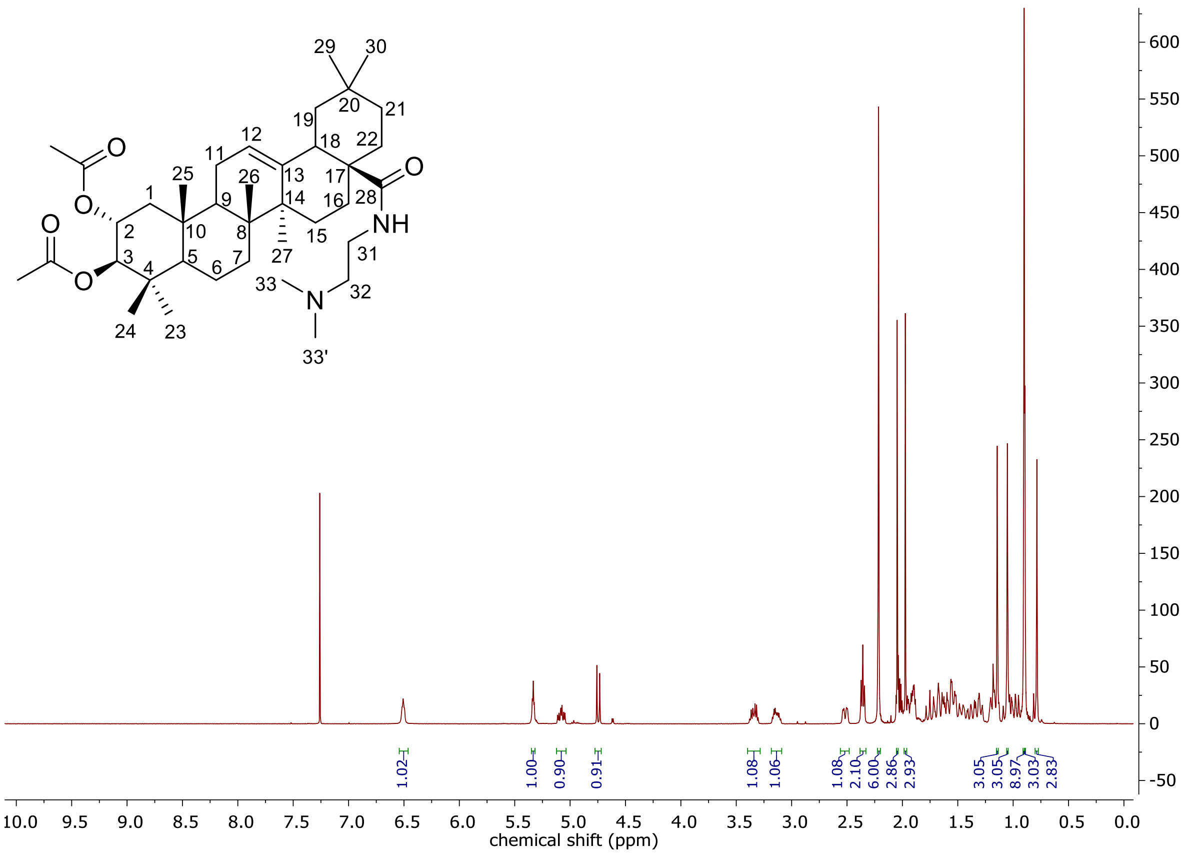

^{13}C APT NMR (101 MHz, $CDCl_3$):

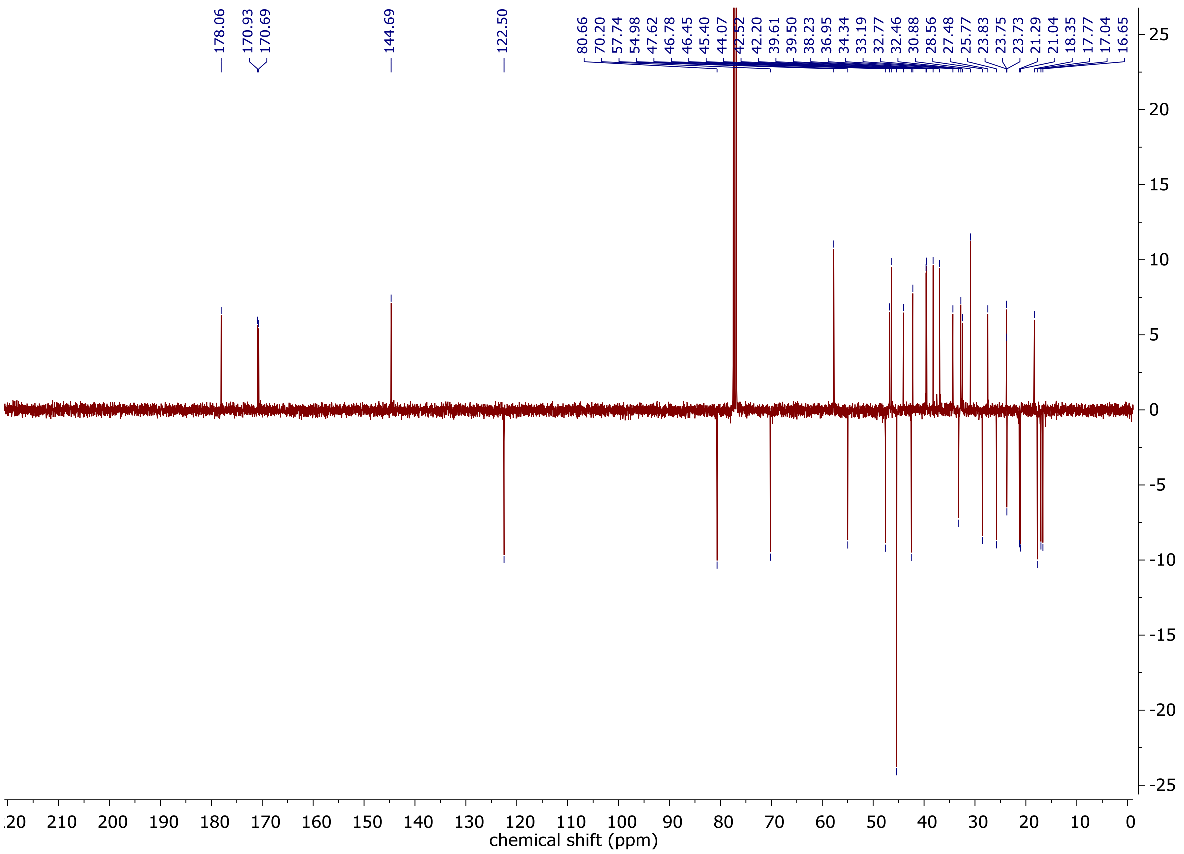

Compound 27:

^{1}H NMR (500 MHz, $CDCl_3$):

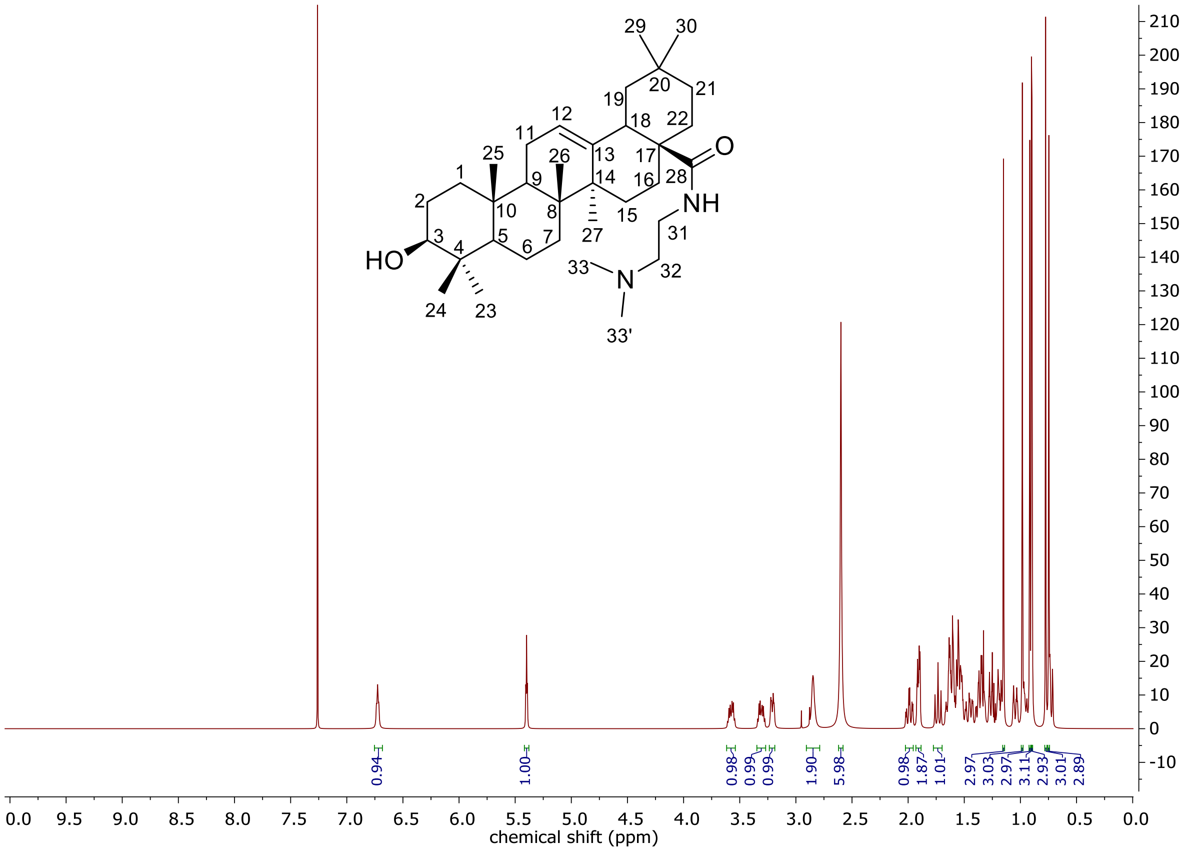

^{13}C APT NMR (126 MHz, $CDCl_3$):

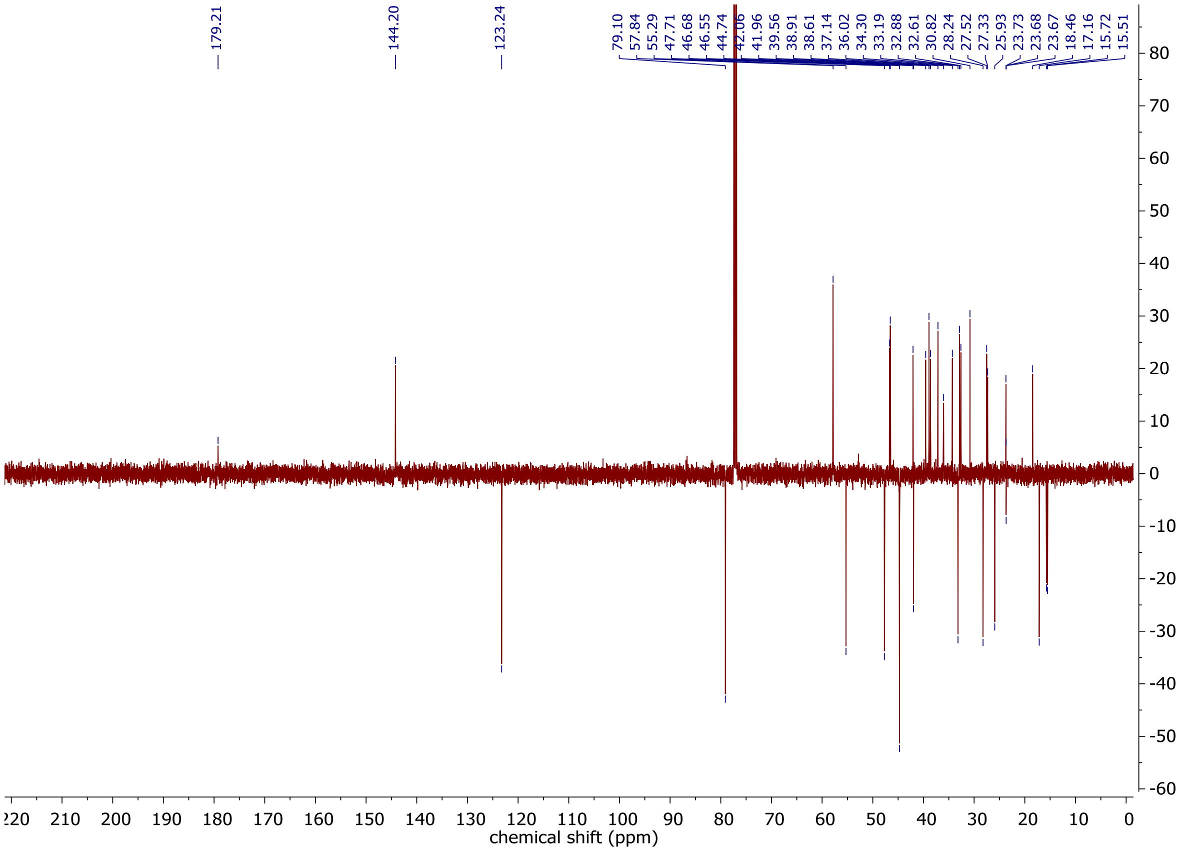

Compound 28:

^{1}H NMR (400 MHz, $CDCl_3$):

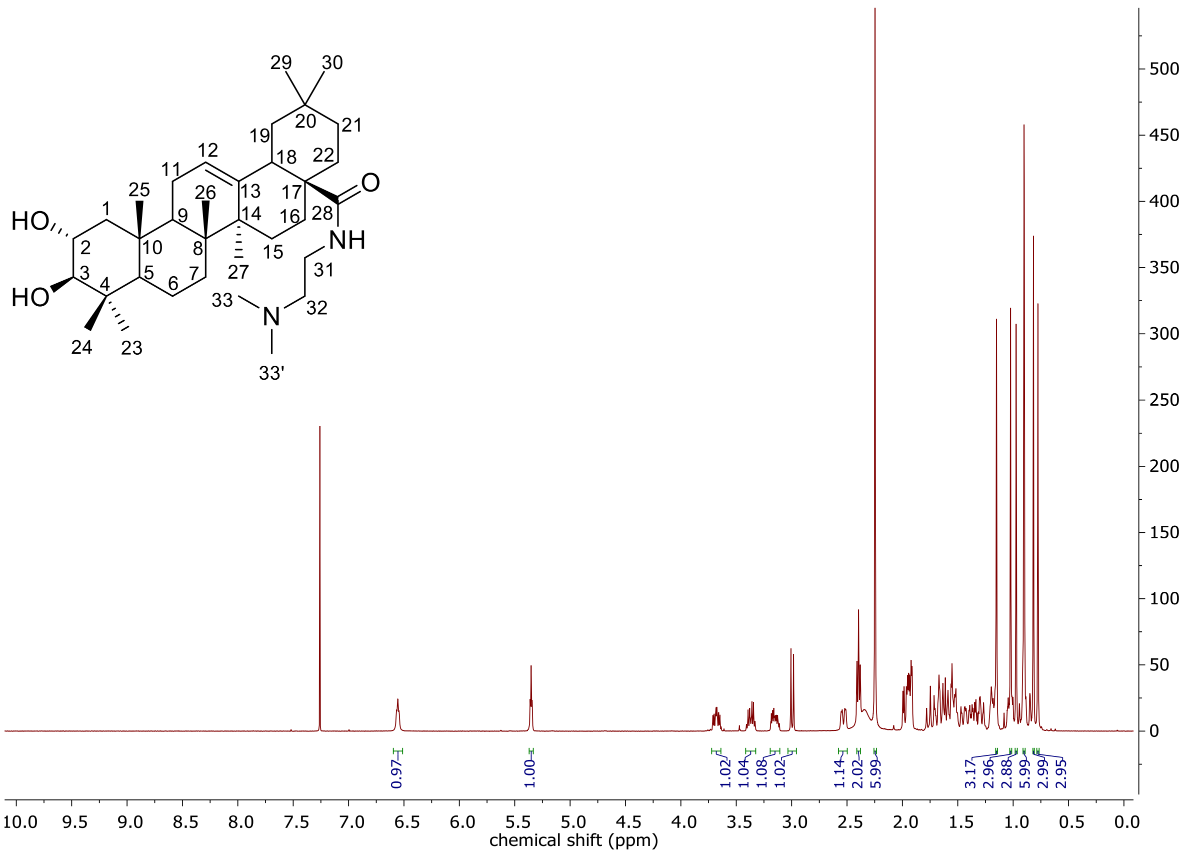

^{13}C APT NMR (101 MHz, $CDCl_3$):

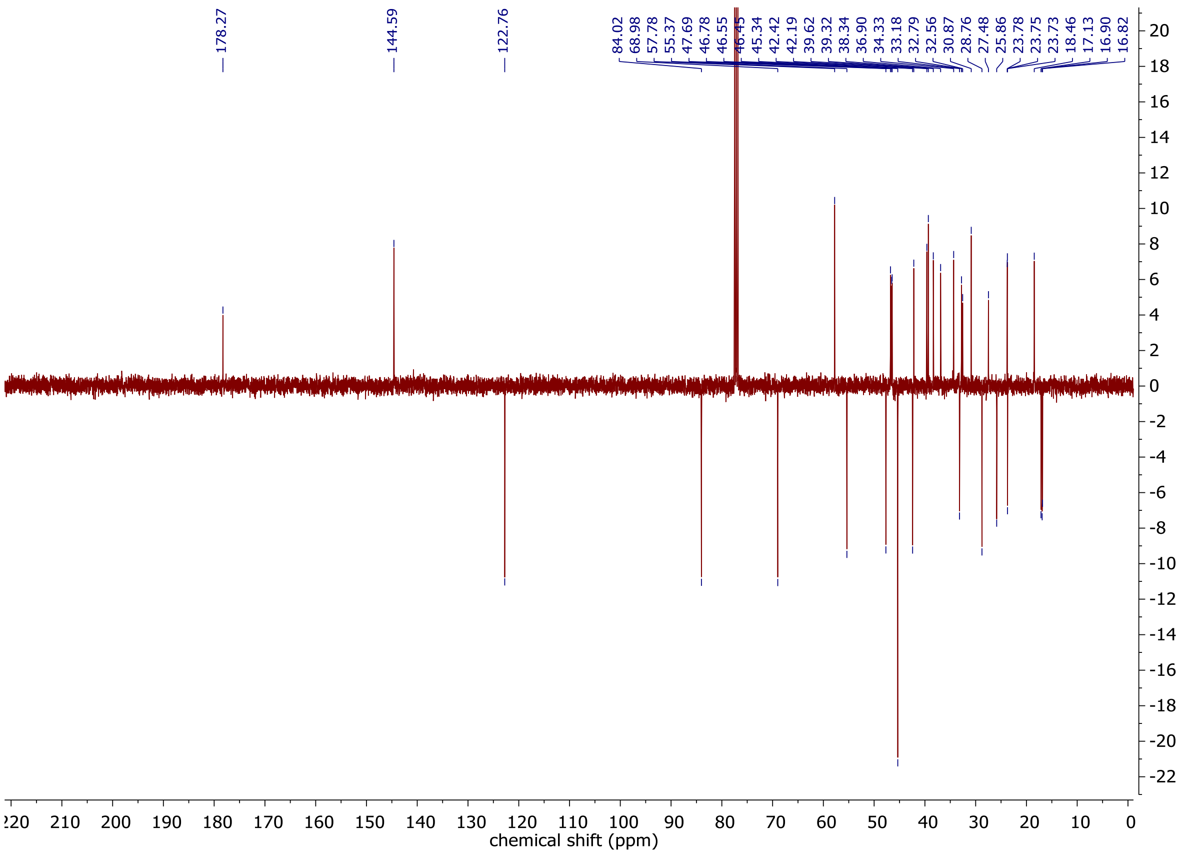

Compound 32:

^{1}H NMR (400 MHz, $CDCl_3$):

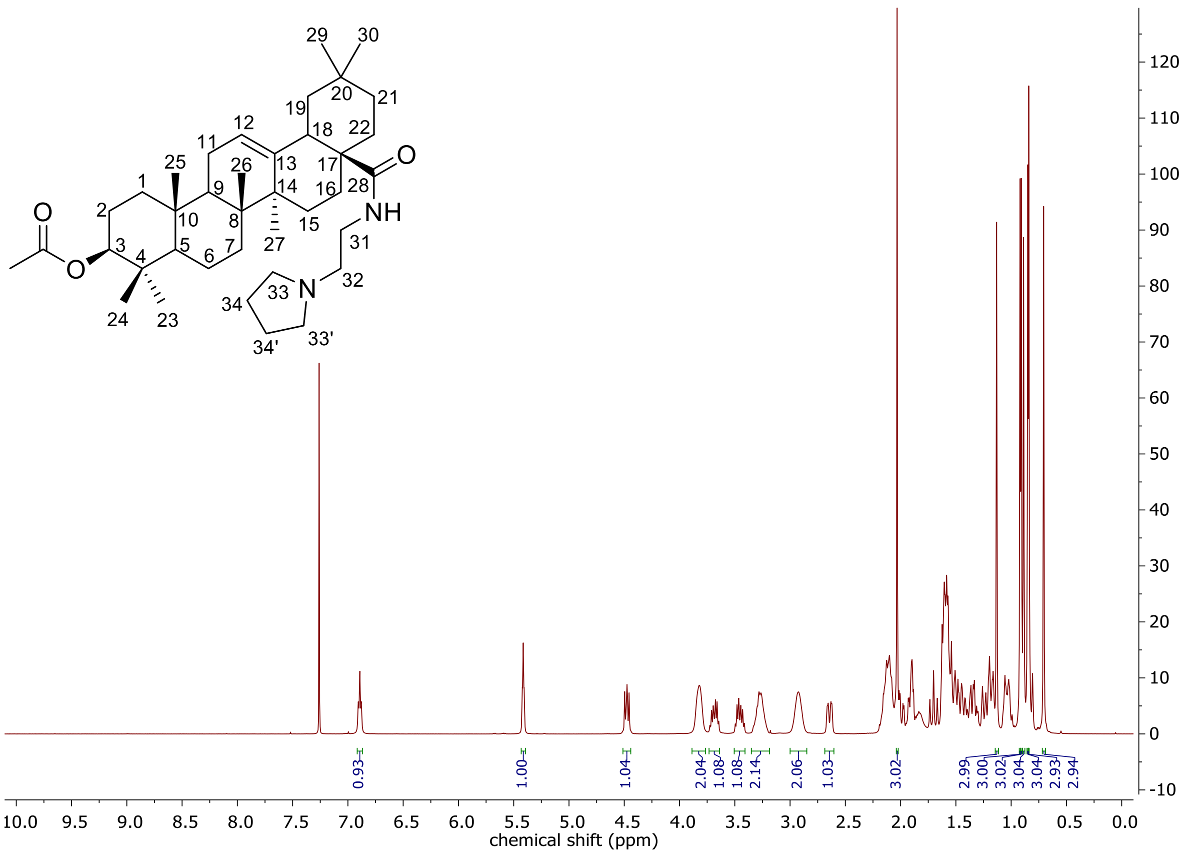

^{13}C APT NMR (101 MHz, $CDCl_3$):

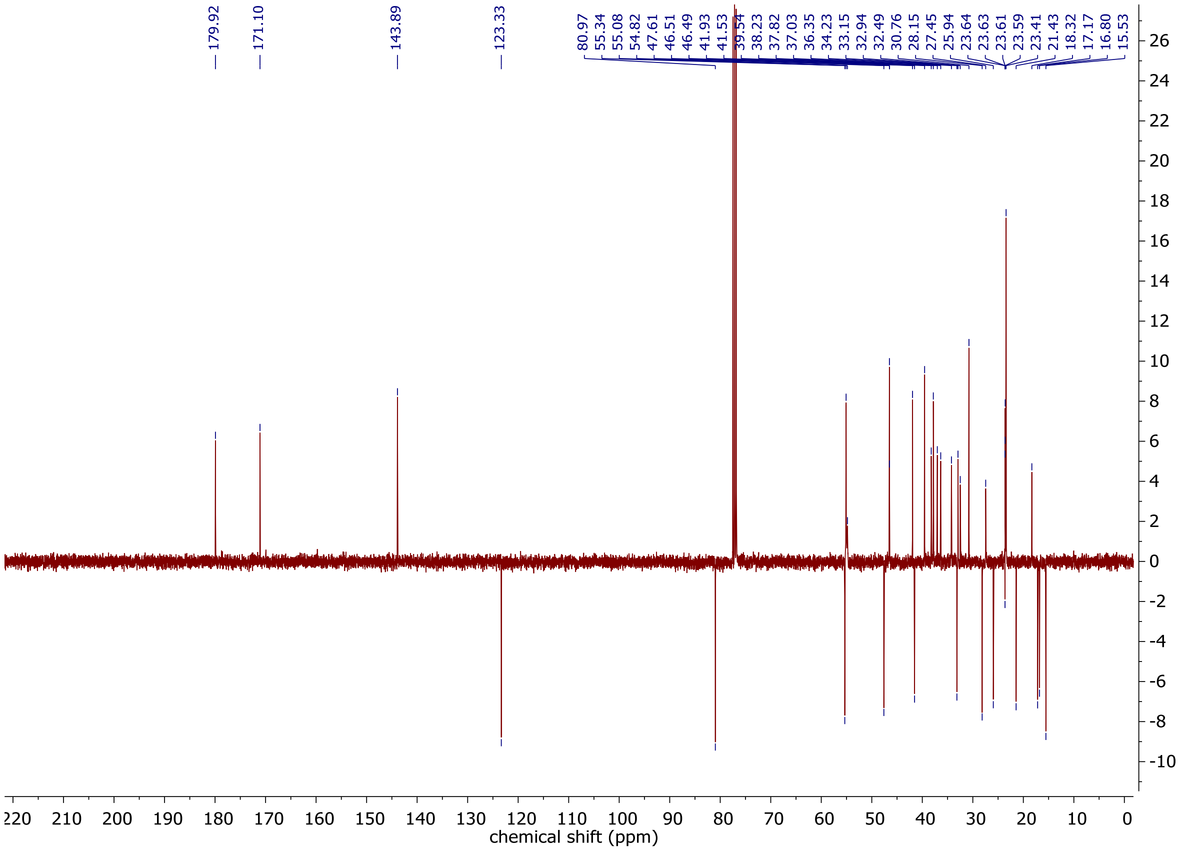

Compound 33:

^{1}H NMR (400 MHz, $CDCl_3$):

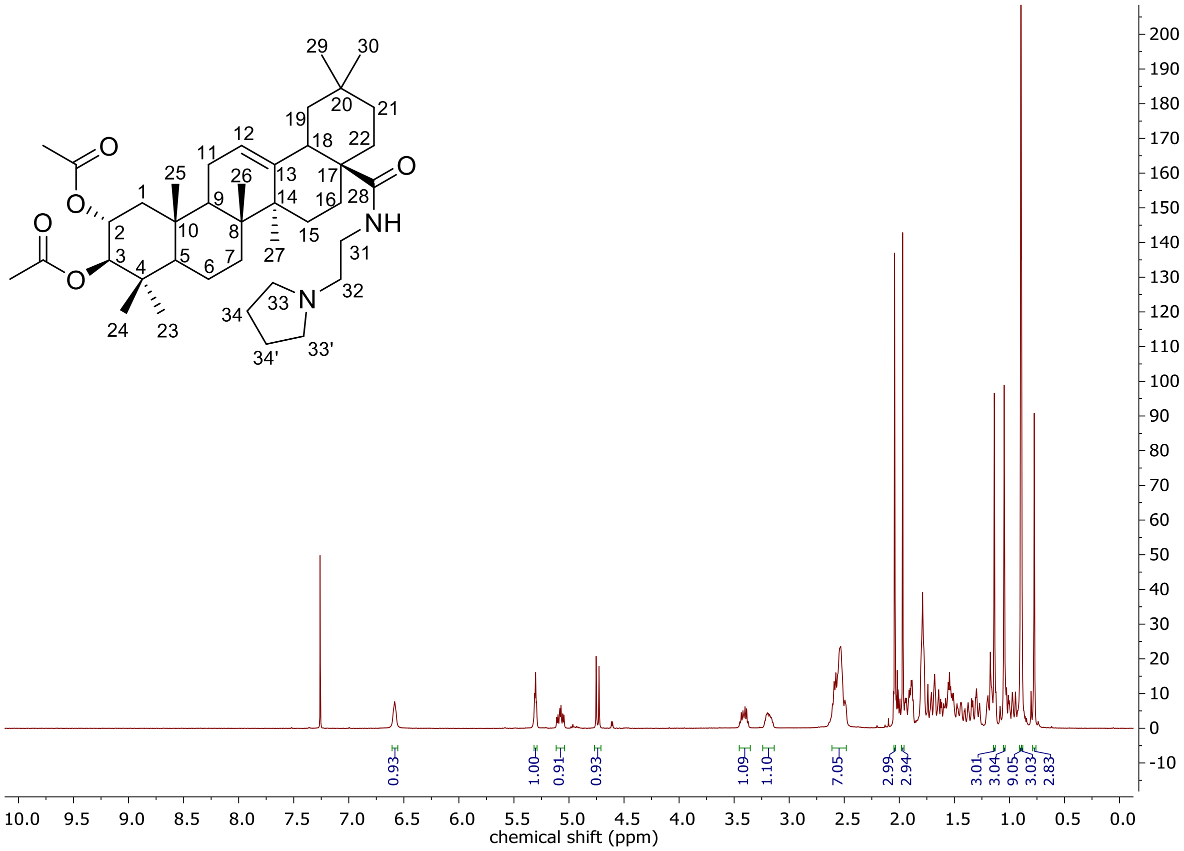

^{13}C APT NMR (101 MHz, $CDCl_3$):

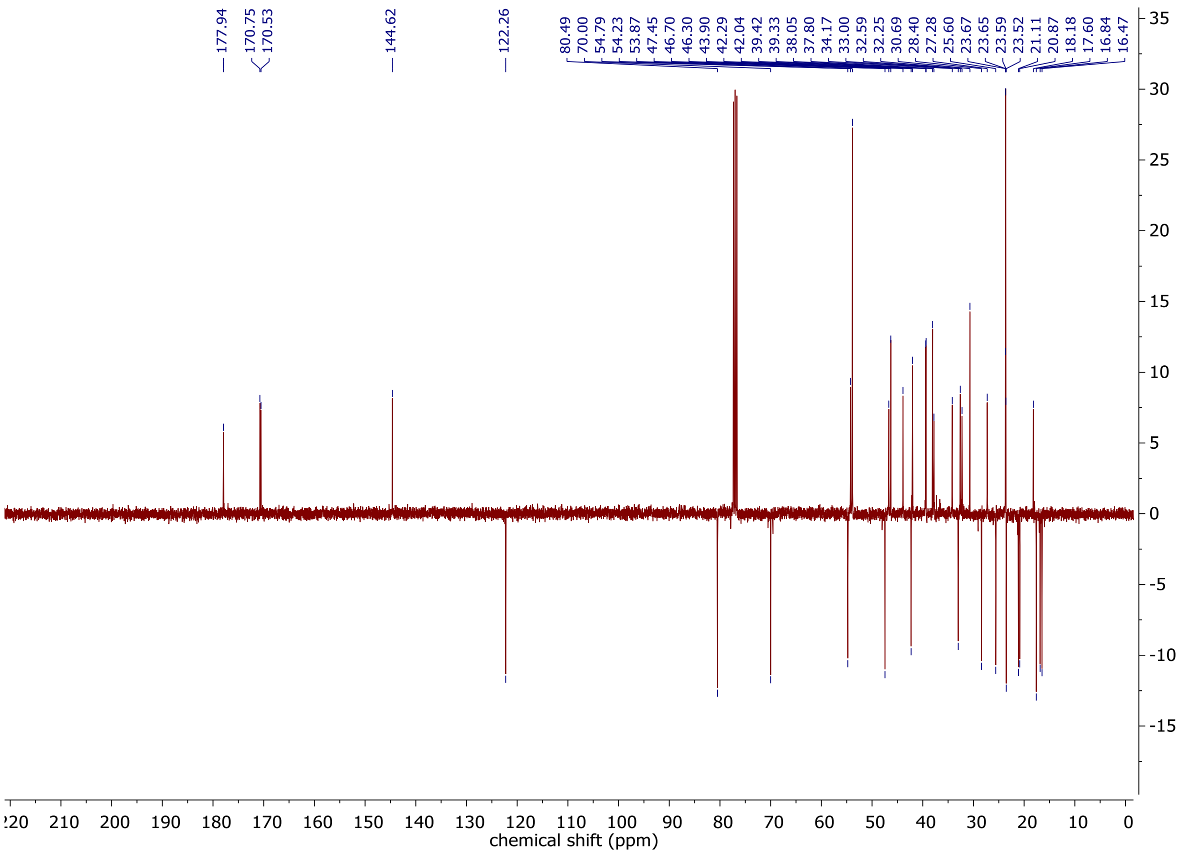

Compound 37:

^{1}H NMR (500 MHz, $CDCl_3$):

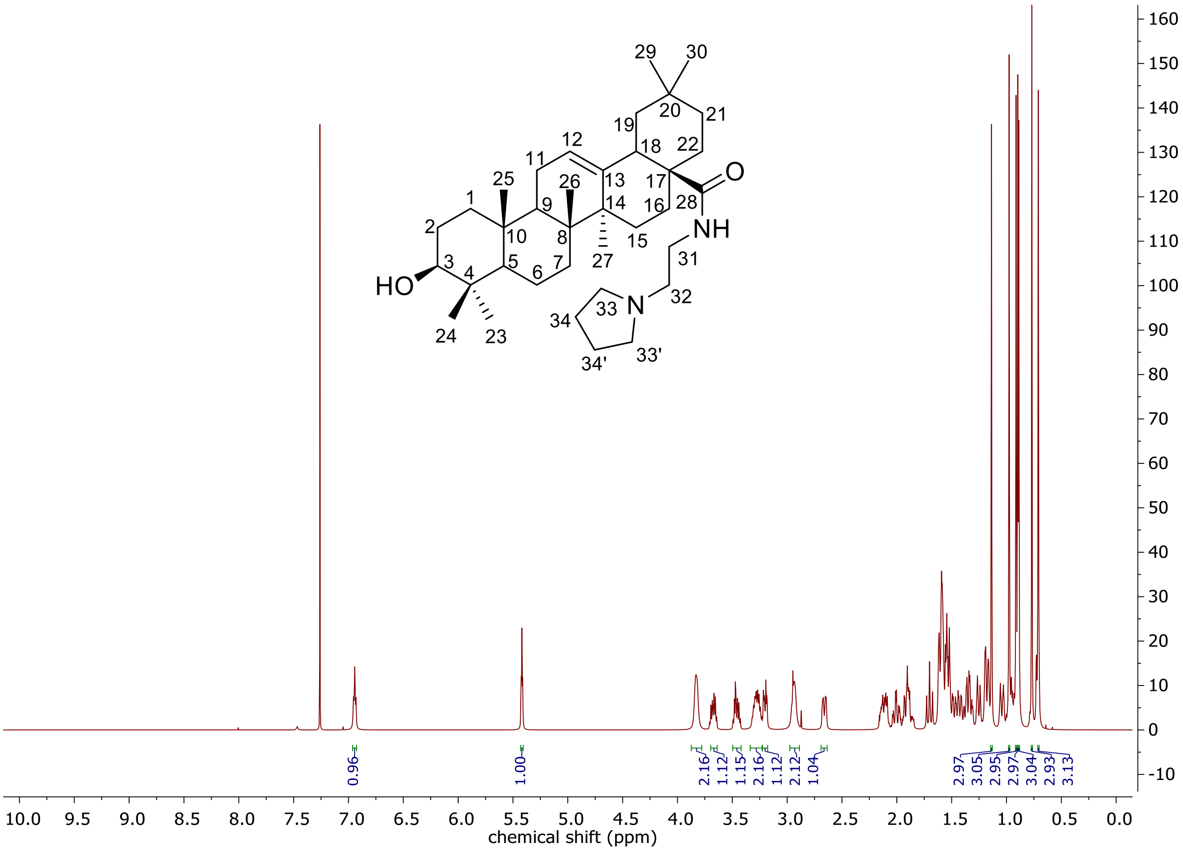

^{13}C APT NMR (126 MHz, $CDCl_3$):

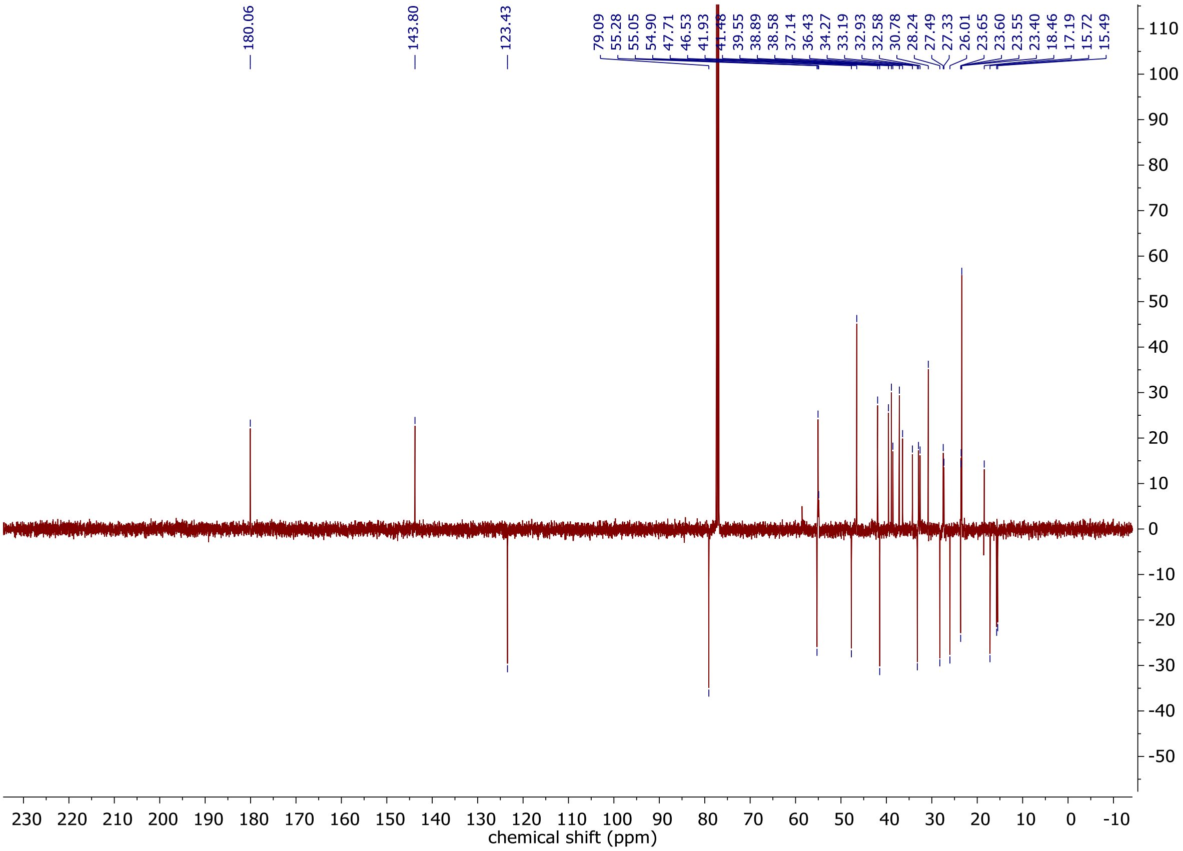

Compound 38:

^{1}H NMR (400 MHz, $CDCl_3$):

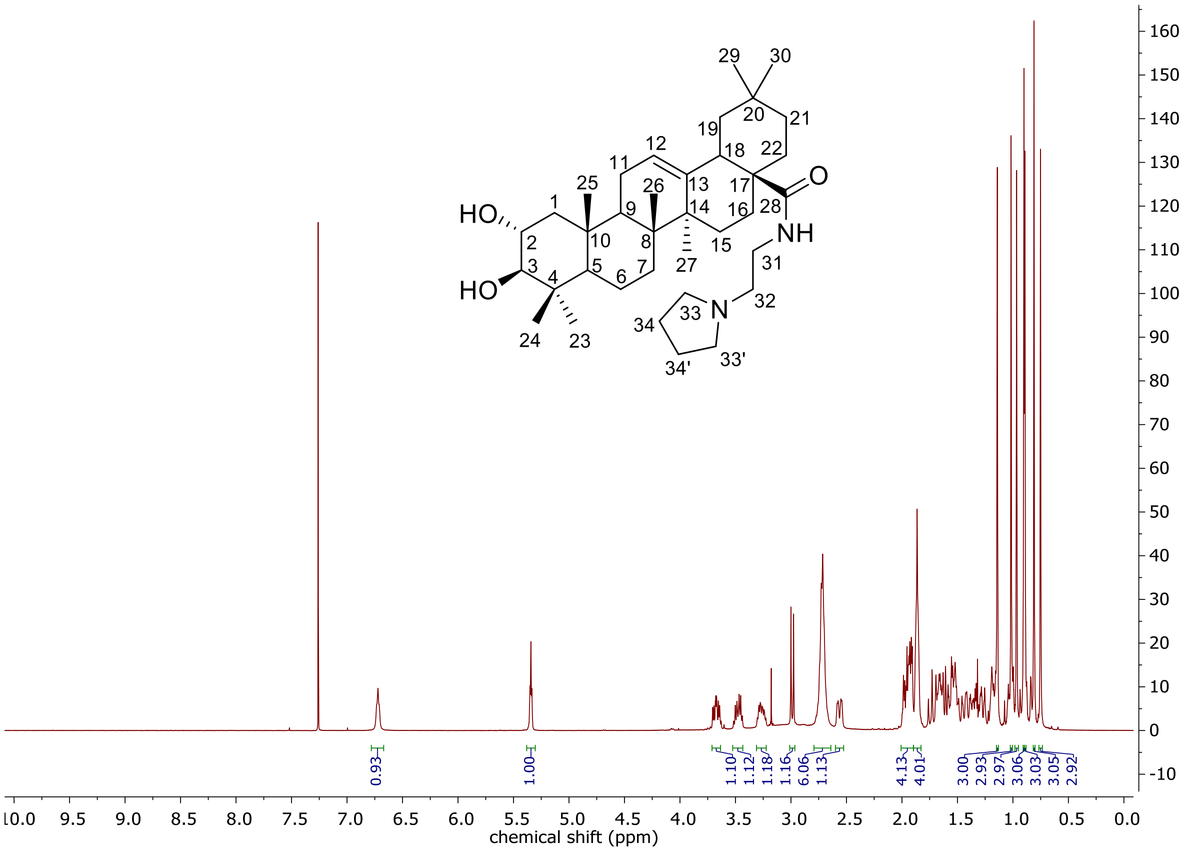

^{13}C APT NMR (101 MHz, $CDCl_3$):

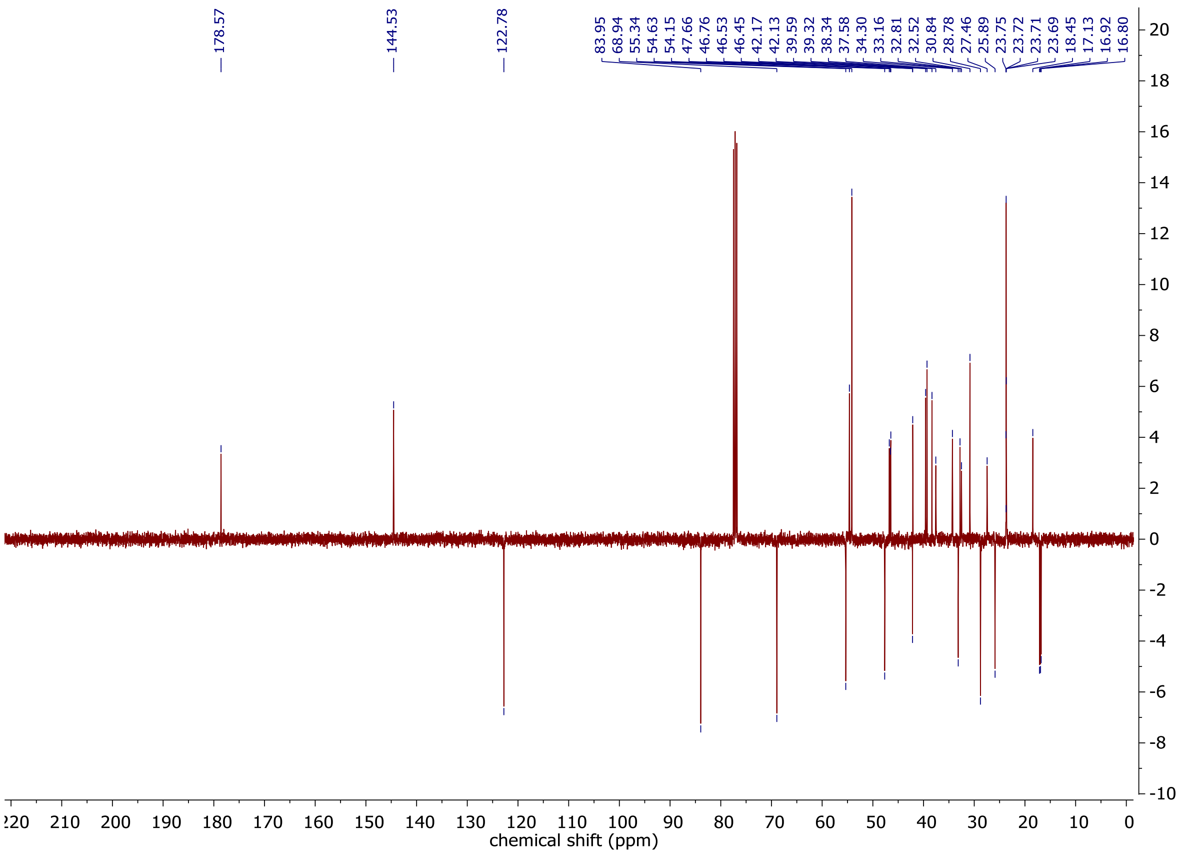

Compound 42:

^{1}H NMR (500 MHz, $CDCl_3$):

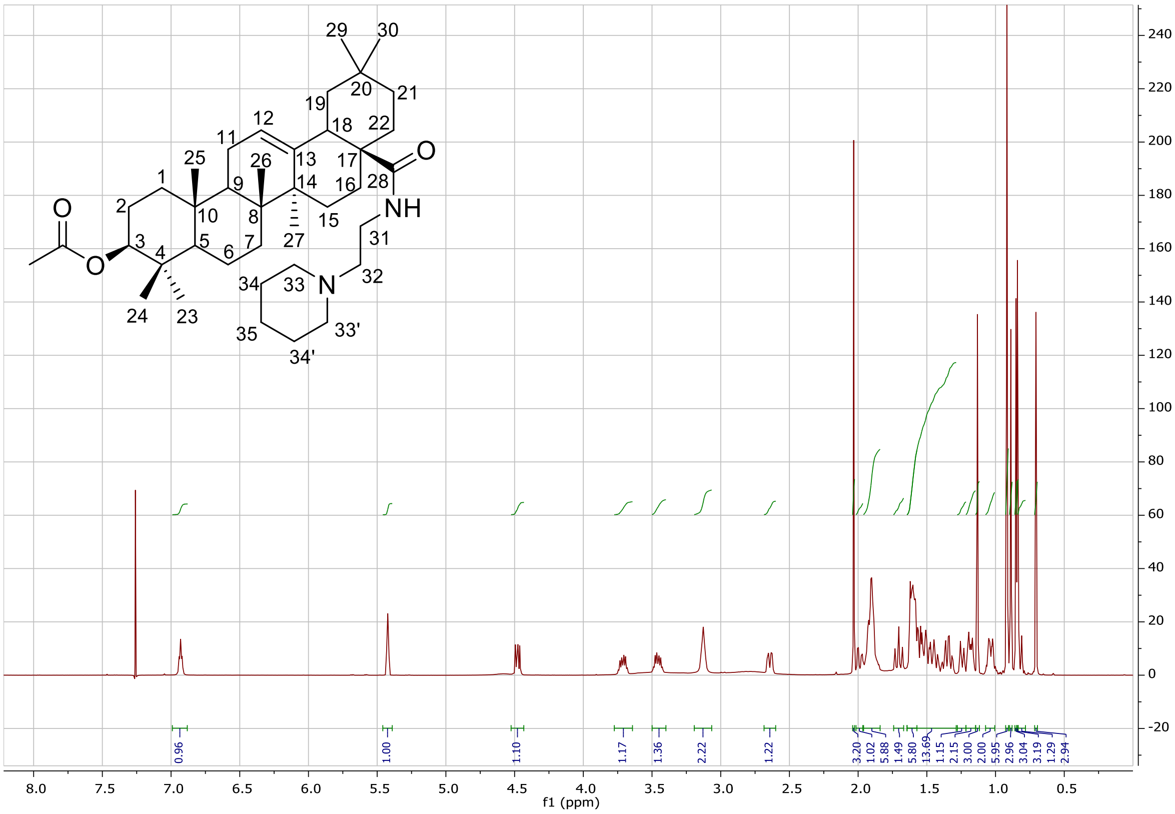

^{13}C APT NMR (126 MHz, $CDCl_3$):

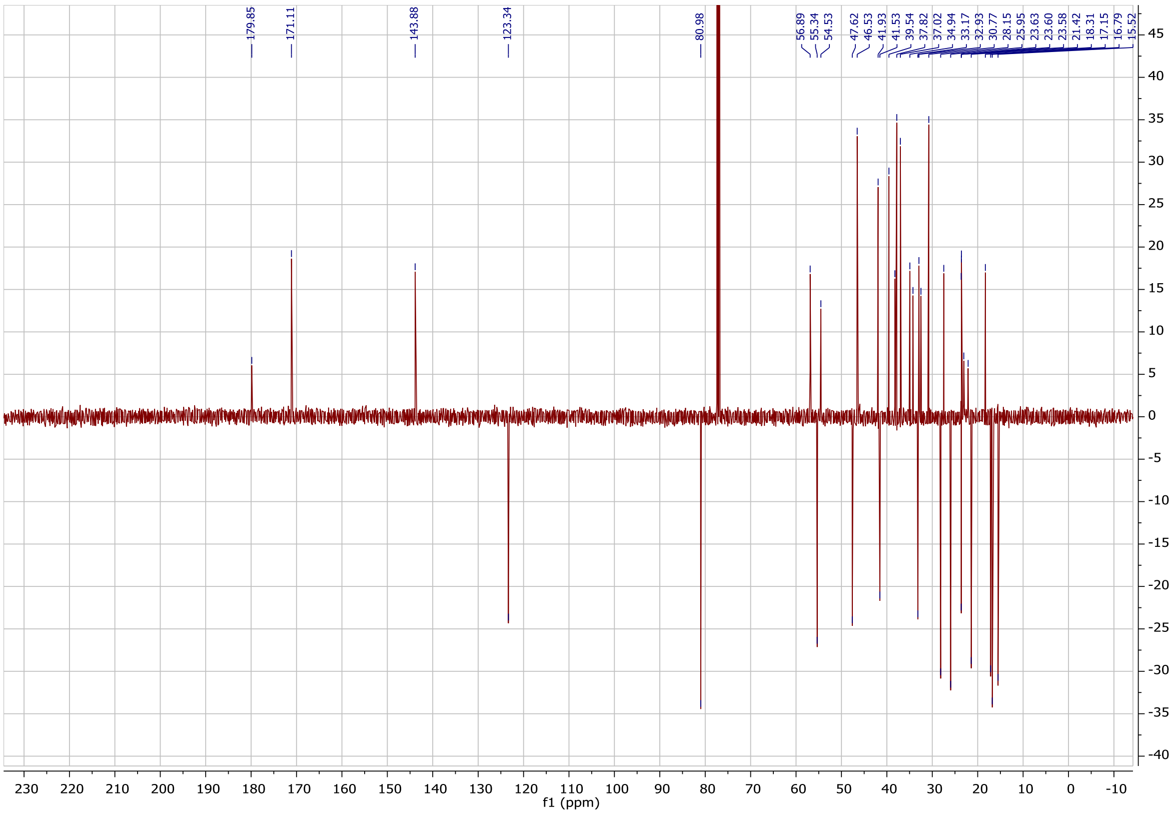

Compound 43:

^{1}H NMR (500 MHz, CDCl$_3$):

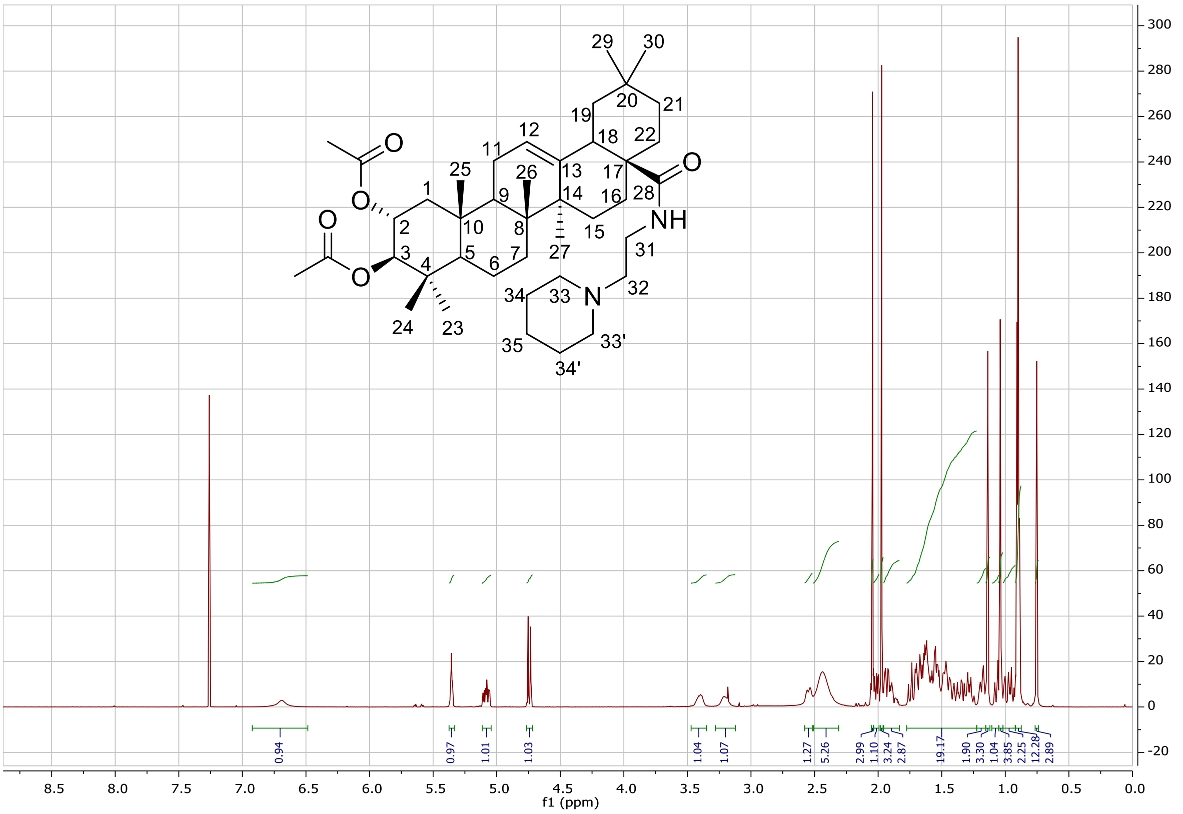

^{13}C APT NMR (126 MHz, CDCl$_3$):

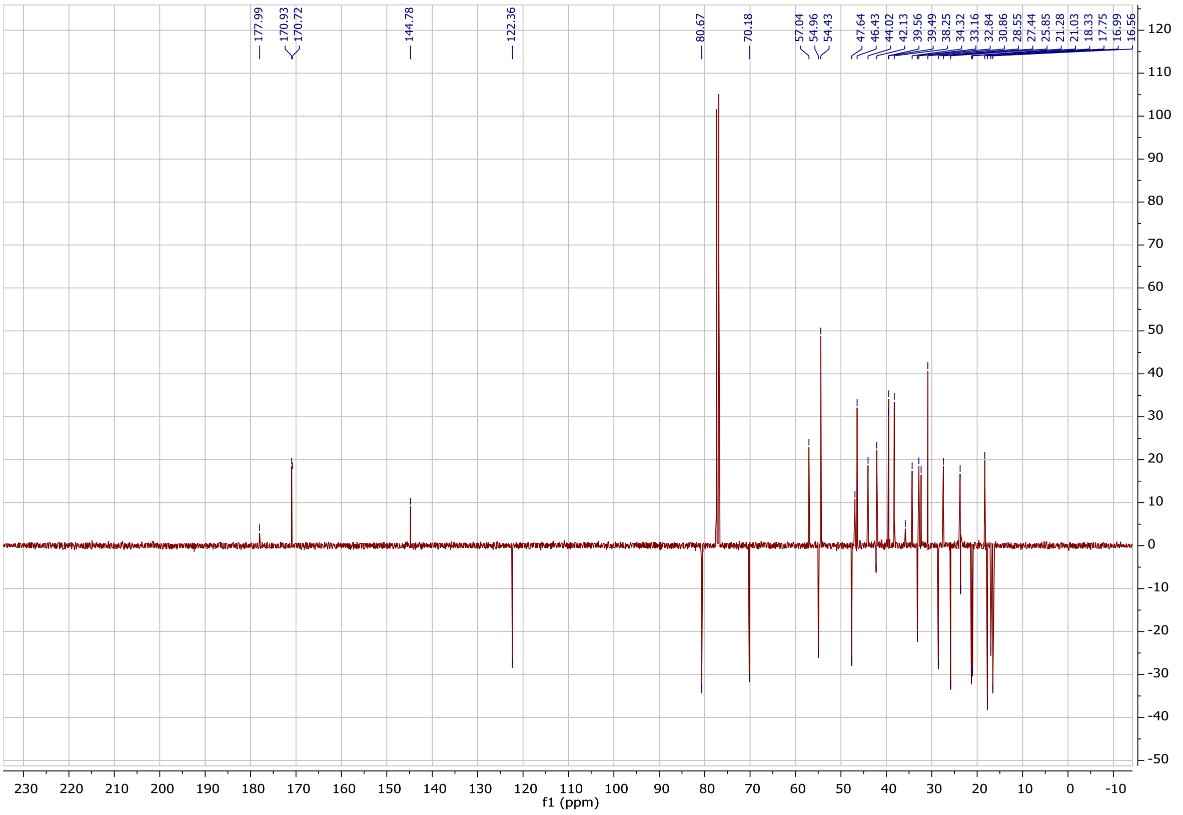

Compound 45:

^{1}H NMR (500 MHz, $CDCl_3$):

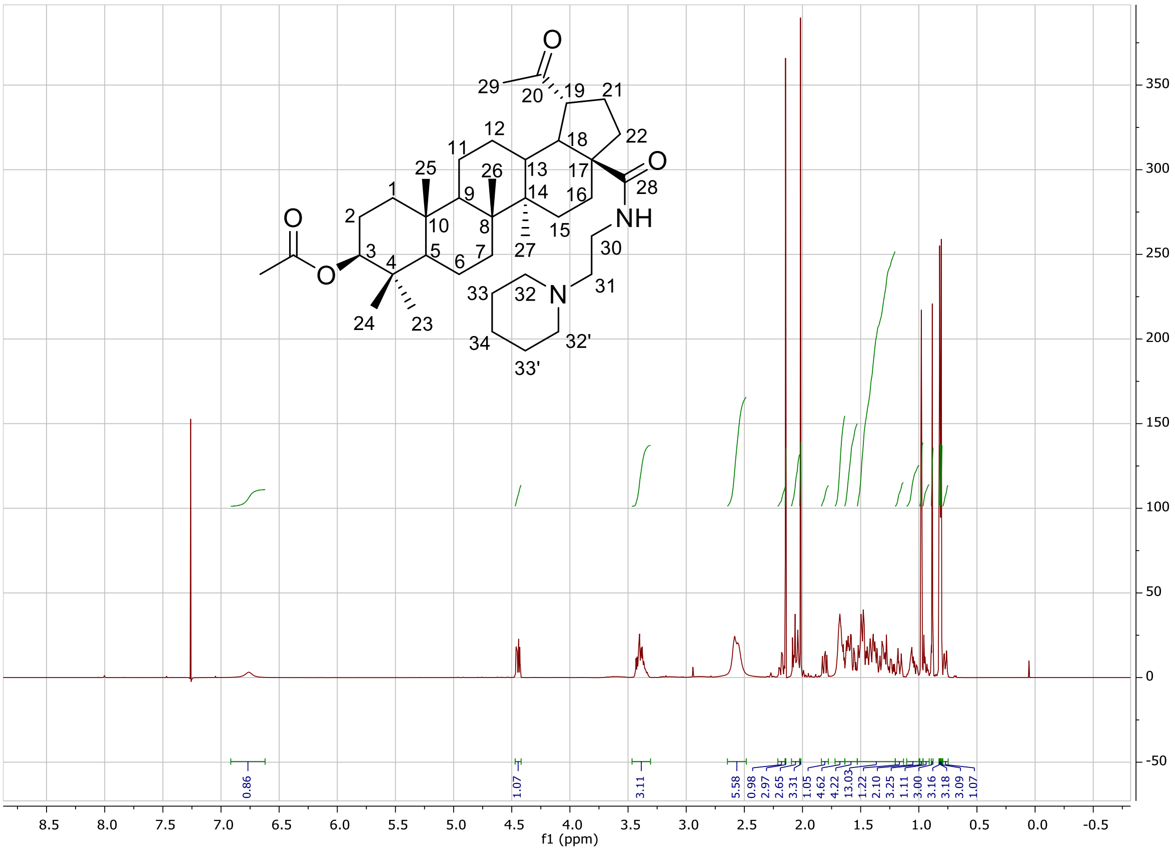

^{13}C APT NMR (126 MHz, $CDCl_3$):

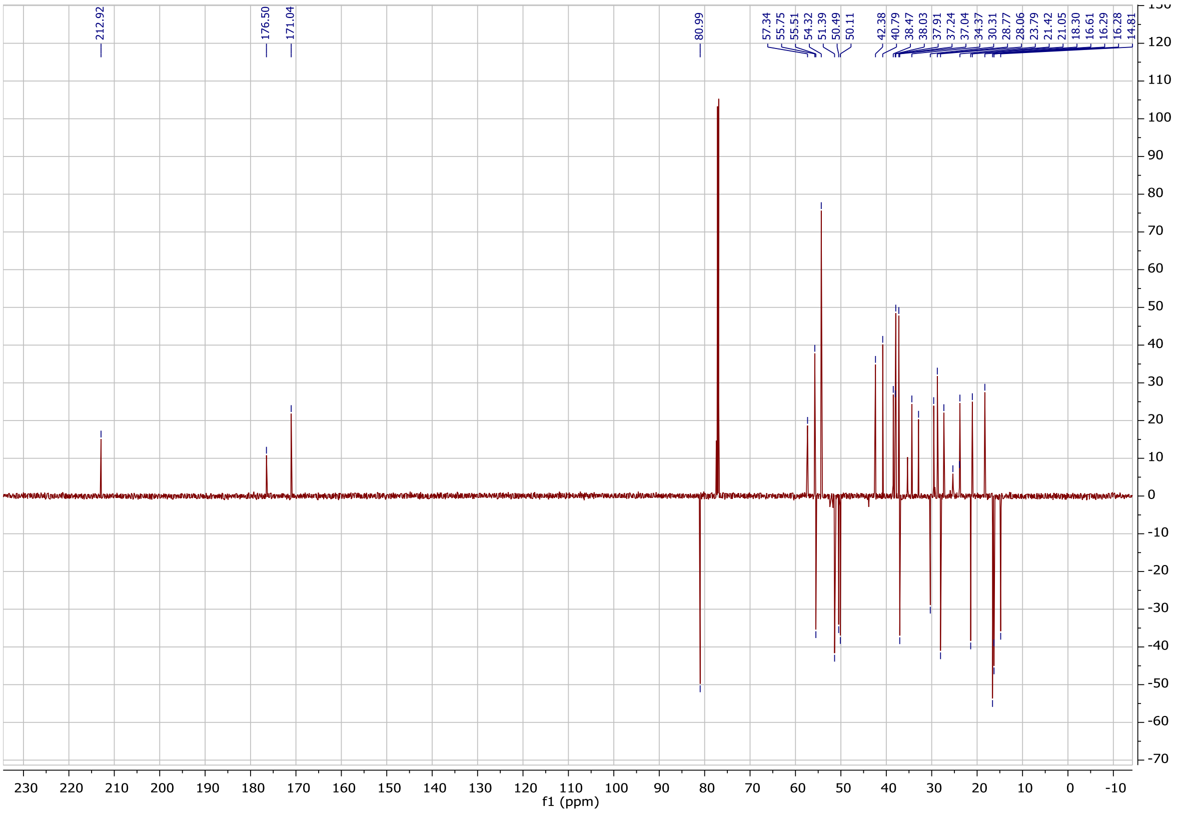

Compound 47:

^{1}H NMR (500 MHz, $CDCl_3$):

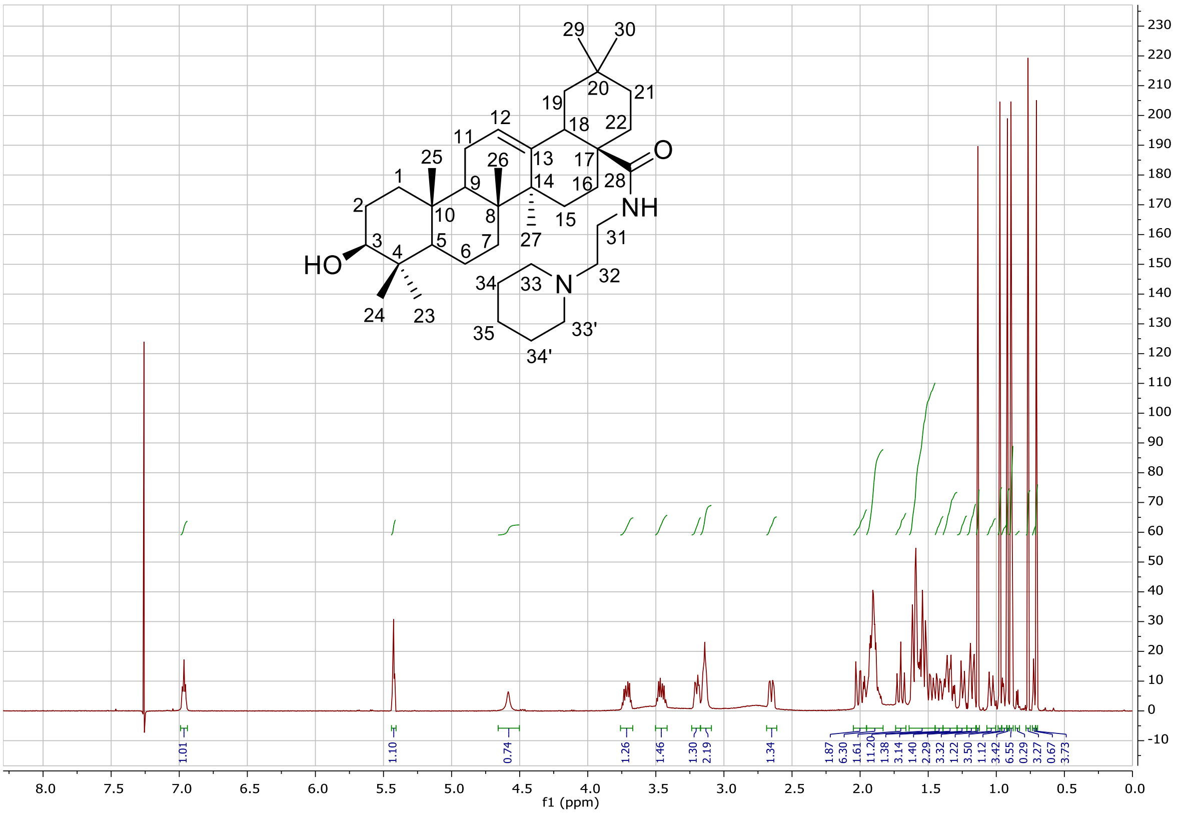

^{13}C APT NMR (126 MHz, $CDCl_3$):

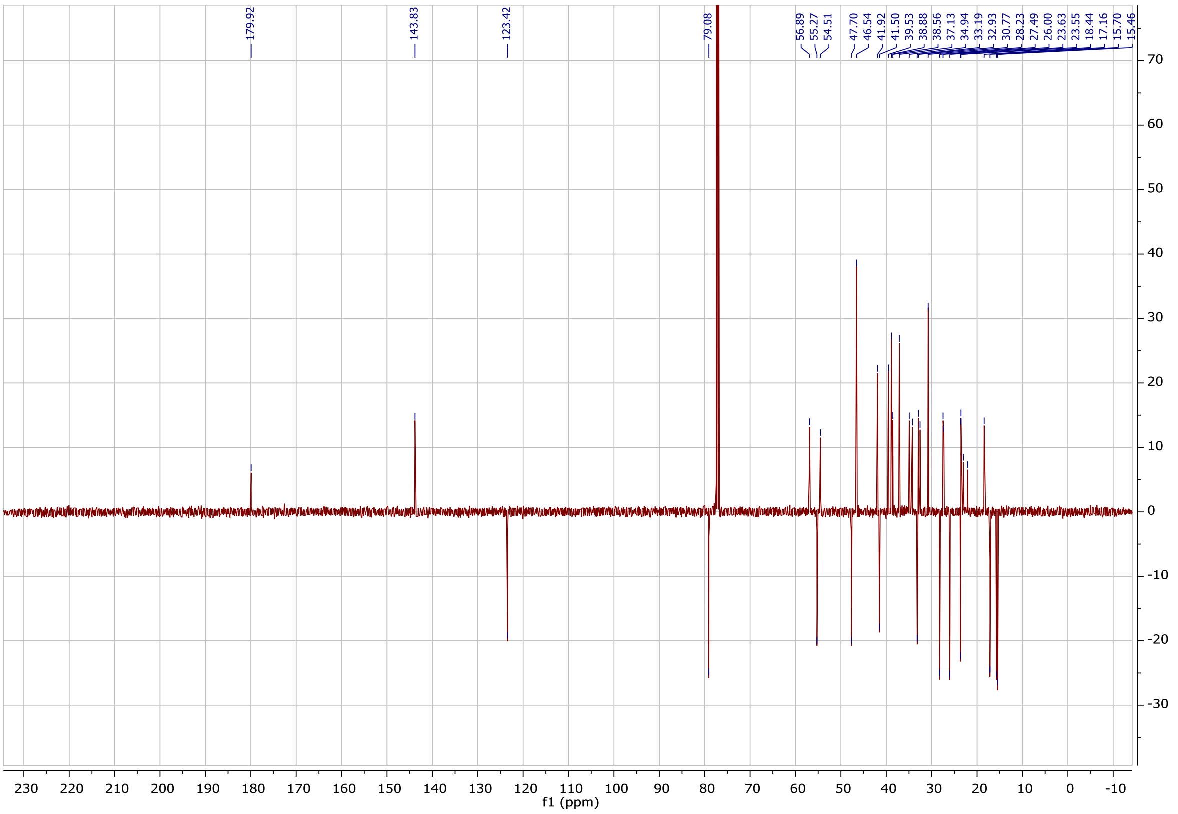

Compound 48:

^{1}H NMR (500 MHz, $CDCl_3$):

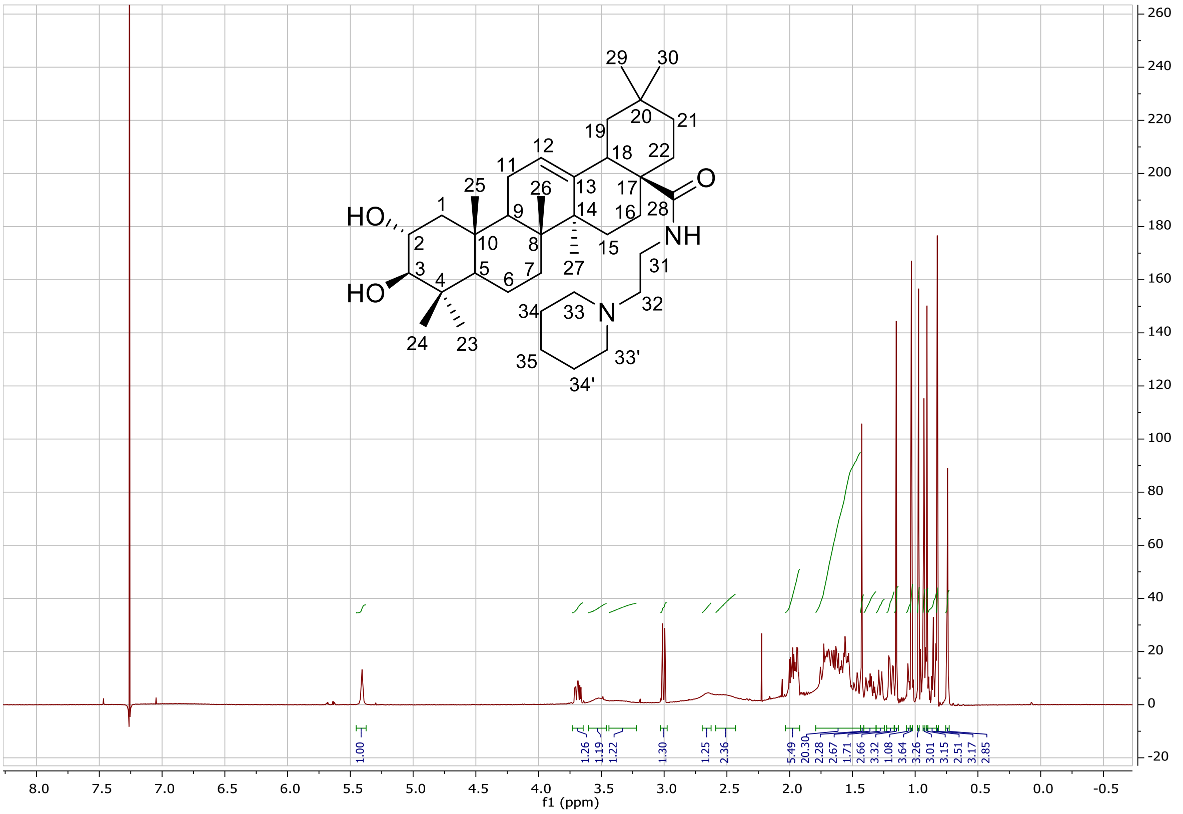

^{13}C APT NMR (126 MHz, $CDCl_3$):

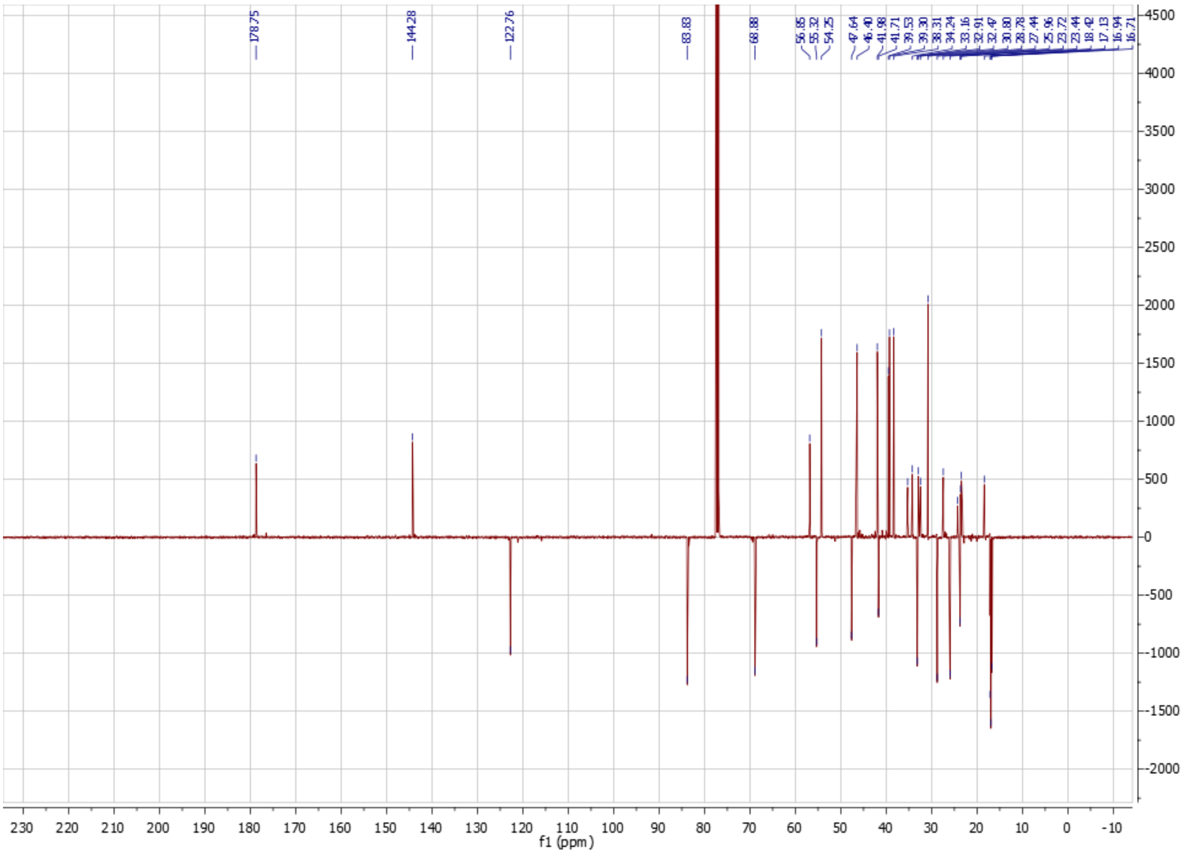

Compound 50:

^{1}H NMR (500 MHz, $CDCl_3$):

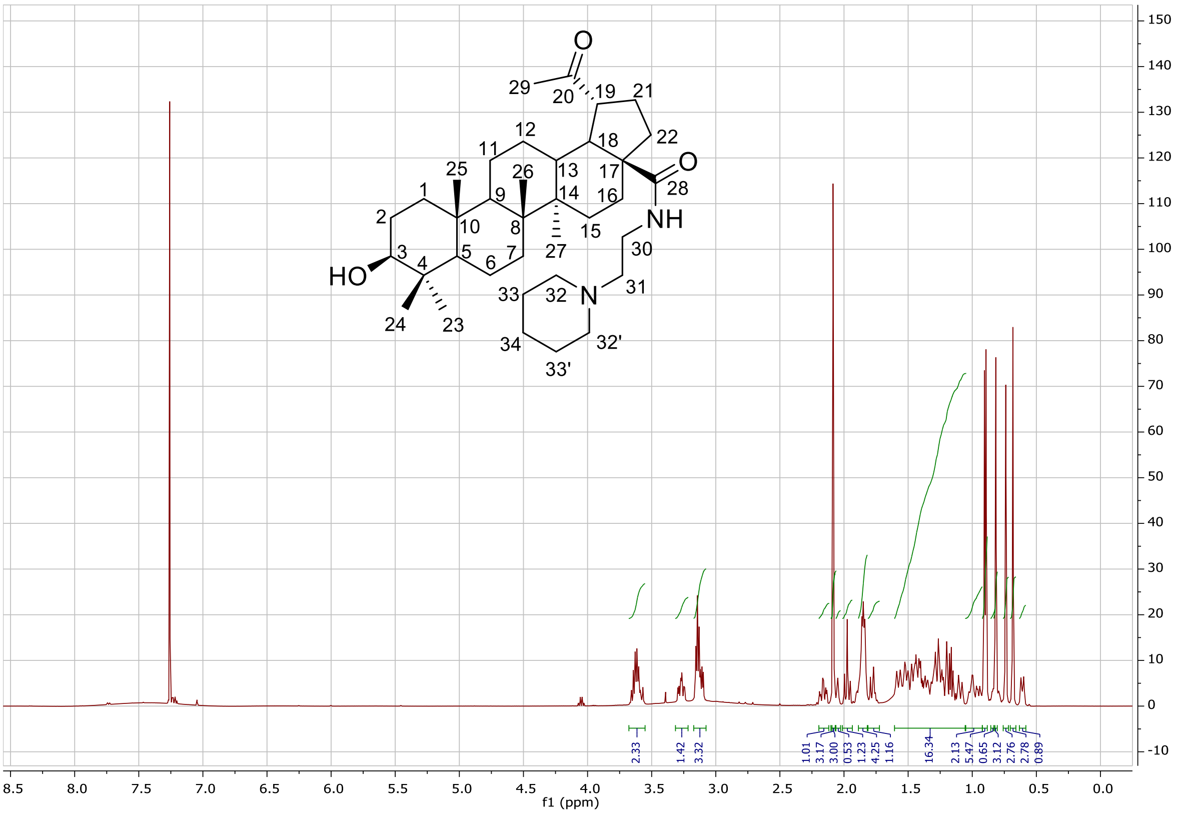

^{13}C APT NMR (126 MHz, $CDCl_3$):

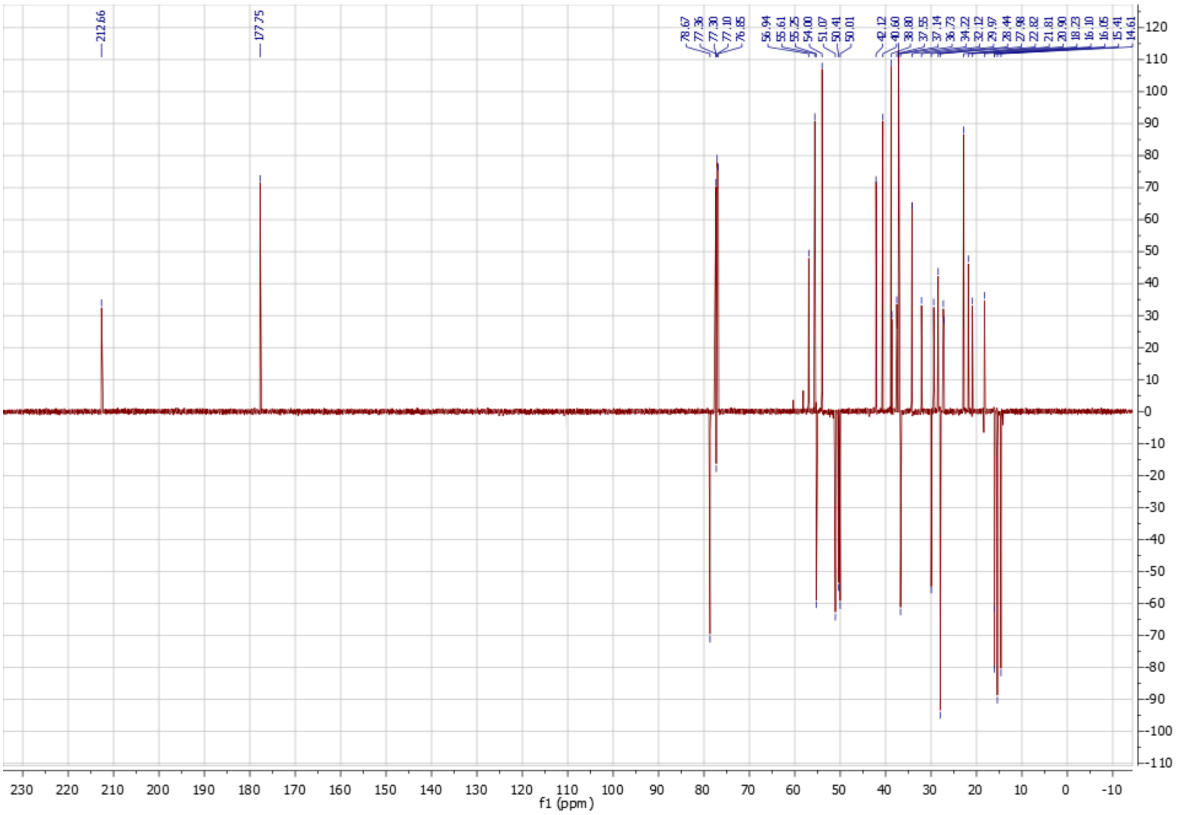

3 Enzymatic studies

3.1 Preparation of the solutions

Preparation of 50 mM Tris-HCl buffer solutions: Tris(hydroxymethyl)-aminomethane (606 mg) was dissolved in bidestilled water (100 mL) and adjusted with HCl to a pH of 8.0 ± 0.1. Buffers were freshly prepared and stored in the refrigerator. AChE solution 2.005 U/mL: the enzyme (271 U/mg, 0.037 mg) was dissolved in freshly prepared buffer pH 8.0 (5 mL) containing NaN_3 (0.98 mg). BChE solution 2.040 U/mL: the enzyme (7.54 U/mg, 1.353 mg) was dissolved in freshly prepared buffer pH 8.0 (5 mL) containing NaN_3 (0.98 mg). DTNB solution 3 mM: DTNB (23.8 mg) was dissolved in freshly prepared buffer pH 8.0 (20 mL) containing NaCl (116.8 mg) and $MgCl_2$ (38.0 mg). ATChI solution 15 mM: ATChI (43.4 mg) was dissolved in bidestilled water (10 mL). All solutions were stored in Eppendorf caps in the refrigerator or in the freezer, if necessary. The pure compounds were initially dissolved in DMSO, galantamine hydrobromide as standard for AChE and BChE was dissolved in bidistilled water. The final concentrations for the enzymatic assay were obtained by diluting the stock solution with bidistilled water. No inhibition was detected by residual DMSO (< 0.5%).

3.2 Cholinesterase Assay

A mixture of the DTNB solution (125 µL), enzyme solution (25 µL) and compounds solutions (25 µL, 3 different concentrations and once blank water) was prepared and incubated at 30 °C for 20 min. The substrate (25 µL, 4 different concentrations) was added to start the enzymatic reaction. The absorbance data (λ = 415 nm) was recorded under a controlled temperature of 30 °C for 30 min at 1 min intervals. The substrate concentrations in the test were as follows: [ATChI] = 0.9375 mM, 0.625 mM, 0.325 mM, 0.1875 mM. The relative inhibition was determined as the quotient of the slopes (compound divided by blank) of the linear ranges. The used substrate concentration was 0.625 mM. The absorbance data was recorded under a controlled temperature of 30 °C for 10 min.

Table 1. Grade of inhibition (in%) of galantamine hydrobromide (**GH** as standard) and compounds **11**–**50** determined by Ellman's assay using acetylcholinesterase (AChE, electric eel) and butyrylcholinesterase (BChE, equine serum).

	Grade of inhibition in% (c = 10 μM)			Grade of inhibition in% (c = 30 μM)	
Compound	**AChE**	**BChE**	Compound	**AChE**	**BChE**
**GH**	89.02 ± 0.12	57.79 ± 0.52	**GH**	95.86 ± 0.17	80.25 ± 0.33
**11**	31.54 ± 0.37	37.86 ± 1.00	**12**	31.69 ± 0.04	57.54 ± 0.93
**13**	31.91 ± 0.32	40.83 ± 0.21	**18**	39.15 ± 0.16	68.83 ± 1.57
**14**	56.17 ± 0.64	42.70 ± 1.33	**26**	33.53 ± 0.44	77.74 ± 0.59
**15**	44.71 ± 0.24	29.55 ± 2.97	**28**	13.47 ± 0.55	79.01 ± 0.29
**16**	28.54 ± 0.51	64.10 ± 1.04	**29**	22.12 ± 4.01	56.68 ± 0.01
**17**	88.61 ± 0.22	49.10 ± 0.47	**30**	28.83 ± 0.80	89.76 ± 0.48
**19**	62.95 ± 0.61	36.70 ± 5.34	**33**	23.47 ± 4.59	33.01 ± 2.91
**20**	37.09 ± 0.97	12.98 ± 0.35	**35**	19.97 ± 0.13	94.60 ± 0.02
**21**	10.78 ± 0.12	36.50 ± 0.30	**36**	31.45 ± 0.60	85.66 ± 0.10
**24**	13.10 ± 1.32	67.75 ± 0.69	**38**	23.33 ± 0.22	77.49 ± 0.10
**25**	no inhibition	88.49 ± 0.58	**39**	58.81 ± 0.80	60.48 ± 0.05
**27**	5.54 ± 0.07	67.10 ± 0.07	**40**	33.06 ± 0.45	89.15 ± 0.44
**31**	14.54 ± 0.36	50.49 ± 2.23	**42**	45.57 ± 0.25	53.09 ± 0.14
**32**	7.29 ± 0.50	65.73 ± 0.14	**43**	23.30 ± 0.10	53.47 ± 3.47
**34**	44.15 ± 0.32	71.56 ± 0.54	**44**	22.78 ± 0.85	33.83 ± 0.43
**37**	10.04 ± 1.70	74.50 ± 0.93	**46**	21.74 ± 0.92	75.72 ± 0.15
**45**	no inhibition	83.44 ± 1.00	**47**	38.65 ± 0.29	74.89 ± 0.04
**48**	9.32 ± 0.29	69.30 ± 1.12			
**49**	82.72 ± 0.09	66.02 ± 0.70		n.sol.	
**50**	52.14 ± 1.55	88.37 ± 0.85		**22/23/41**	

n.sol. stands for non-soluble under the conditions of the assay; mean ± SE.

Table 2. Significant results of the AChE inhibition assay. Inhibitory constants [K_i (competitive inhibition) and K_i' (uncompetitive inhibition) in μM], determined using Ellman's assay employing acetylcholinesterase (AChE, electric eel) with galantamine hydrobromide (**GH**) as standard.

Compound	K_i in μM / K_i' in μM	Type of inhibition
**GH**	0.37 ± 0.14	competitive
**15**	3.06 ± 0.38 / 6.83 ± 0.17	mixed-type
**17**	8.53 ± 0.34 / 18.24 ± 0.06	mixed-type
**20**	7.18 ± 0.02 / 9.71 ± 0.03	mixed-type
**30**	15.50 ± 3.91 / 74.05 ± 4.81	mixed-type
**40**	27.95 ± 2.99 / 23.63 ± 0.39	mixed-type
**49**	1.00 ± 0.09 / 1.42 ± 0.08	mixed-type

mean ± SE

4 Molecular modelling

For the modelling the structure of human butyrylcholinesterase with the pdb-code 4BDS in complex with tacrine[2] was used. The protein structure was prepared by addition of protons using the "3d-protonate" option in MOE version 2016.08.[3] The 3d-structures of both ligands were constructed and energy was optimized using the MMFF94 force field.[4]

50 docking runs for each ligand were performed with GOLD using the ChemPLP scoring functions.[5] For all other options in GOLD standard settings were applied. A radius of 20 Å was applied to define the active site for docking using the coordinates of the Oε-atoms of E197 in BChE as origin. The side chains were considered of F326 and Y329 to be flexible. The most favoured docking position were finally energy optimized using the AMBER14:EHT force field embedded in MOE with the born salvation option for the treatment of electrostatics by fixing all backbone atoms of the protein. From these optimized complexes the resulting interaction energy of each ligand with BChE were calculated.

5 References

[1] K.-K. Bai, Z. Yu, F.-L. Chen, F. Li, W.-Y. Li, Y.-H. Guo, *Bioorg. Med. Chem. Lett.* **2012**, *22*, 2488–2493.

[2] F. Nachon, E. Carletti, C. Ronco, M. Trovaslet, Y. Nicolet, L. Jean, P.-Y Renard, *Biochem. J.* **2013**, *453*, 393-399.

[3] Chemical Computing Group Inc., Molecular Operating Environment (MOE), 1010 Sherbrooke St. West, Suite #910, Montreal, QC Canada, H3A 2R7, **2016.**

[4] T. A. Halgren, B. L. Bush, *Abstr. Pap. Am. Chem. Soc., 212th ACS Nat. Meeting, Orlando, FL, COMP-002* **1996**.

[5] G. Jones, P. Willett, R. C. Glen, A. R. Leach, R. Taylor, *J. Mol. Biol.* **1997**, *267*, 727-748.
